# Recent advances in low-temperature ceramic fuel cells: material design and applications

**DOI:** 10.1039/d6sc00598e

**Published:** 2026-03-24

**Authors:** Ying Zhang, Rui Guo, Yu Shen, Tianmin He

**Affiliations:** a Key Laboratory of Physics and Technology for Advanced Batteries, Ministry of Education, College of Physics, Jilin University Changchun 130012 China hetm@jlu.edu.cn; b College of Police Equipment and Technology, China People's Police University Langfang 065000 China; c School of Materials Science and Engineering, Changchun University of Science and Technology Changchun 130022 China shenyu@cust.edu.cn

## Abstract

Ceramic fuel cells (CFCs) are highly efficient and clean electrochemical energy conversion devices, featuring a wide range of available fuels (hydrogen, methane, ethanol and biomass gas) and the absence of the need for precious metal catalysts. They will play an important role in the future development of sustainable energy. Compared with high-temperature CFCs, low-temperature CFCs (LT-CFCs) have the advantages of a broader selection of materials, lower material cost, shorter start-up time, and enhanced thermal cycling durability. However, as the operating temperature decreases, the ionic conductivity of the electrolyte and the catalytic activity of the electrodes (cathode and anode) also significantly decrease, leading to a sharp decline in CFC performance. To address these issues, researchers have made significant efforts in the design and development of LT-CFC materials, including the design concept, crystal structure and properties, composition, microstructure and performance optimization of the materials. In this review, we systematically summarize the research progress in the design and development of key materials (electrodes and electrolytes) for LT-CFCs over the last decade, especially focusing on the new materials designed by various strategies, the rationale behind chosen solutions and the applications of these materials in LT-CFCs, specifically including machine learning, density functional theory calculations, high-entropy strategies, defect engineering, mechanical mixing, impregnation strategy, self-assembly, and surface reconstruction. Some potential challenges and prospects of key materials for LT-CFCs in the future are suggested.

## Abstract 2

Schematic diagram of ceramic fuel cells, functions of key materials and design methods.

## Graphical abstract

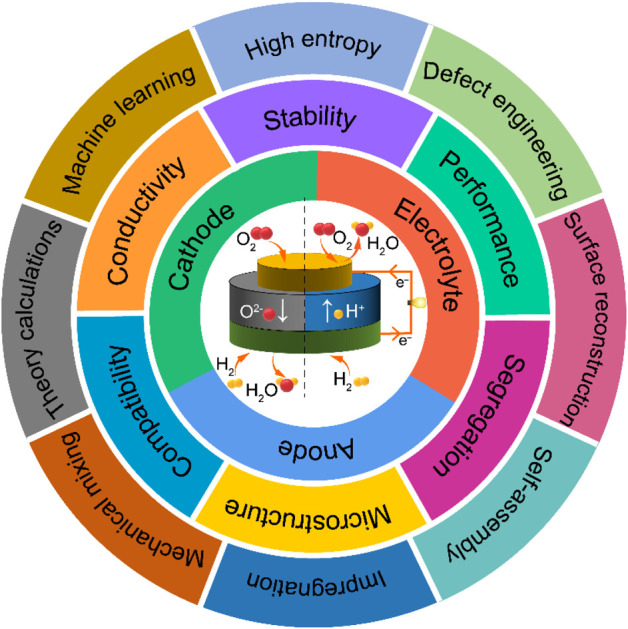

## 1. Introduction

The issue of global climate change is becoming increasingly severe, and all countries are actively seeking solutions to reduce greenhouse gas emissions and achieve sustainable development.^1–3^ Ceramic fuel cells (CFCs) perfectly meet this global strategic demand with their high energy conversion efficiency and environmental friendliness. Therefore, developing efficient, flexible and environmentally friendly energy solutions has become an urgent task.^4–14^ CFCs, also known as solid oxide fuel cells (SOFCs), are all-solid-state chemical power generation devices that directly convert the chemical energy stored in fuels and oxidants into electrical energy. They have the characteristics of high efficiency, no pollution and wide adaptability to various fuels. The main components of a CFC consist of an electrolyte, a cathode (air electrode or oxygen electrode) and an anode (fuel electrode), which are the most critical components of CFCs, so their performance (such as charge and non-charge transport) directly determines the performance of CFCs.^4–6,8–11^

**Fig. 1:**
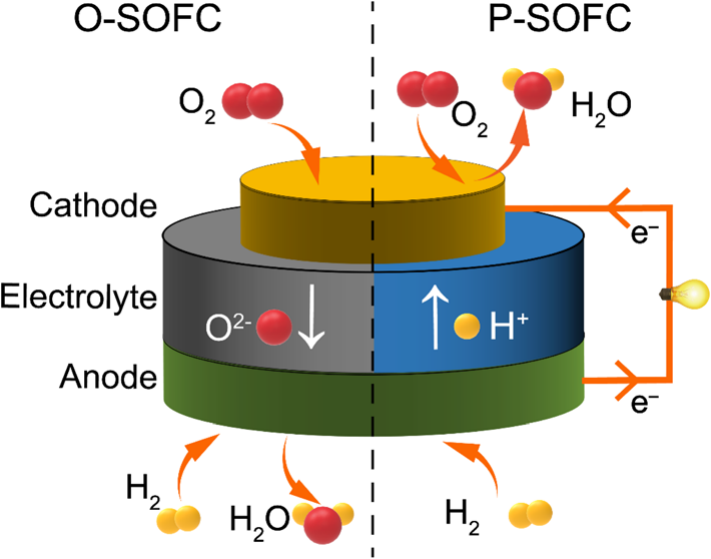
Schematic diagram of a ceramic fuel cell.

The working principle of CFCs is similar to that of other fuel cells.^13^ The electrolyte of CFCs mainly serves to transfer ions (such as oxygen ions or protons) and isolate the fuel gas from the oxidant gas. It needs to have high ionic conductivity, ultra-low electronic conductivity and good chemical and structural stability. The anode is the site where the fuel undergoes oxidation, and the cathode is the site where the oxidant is reduced. Both electrodes have catalytic functions that accelerate the electrochemical reactions of the electrodes. For oxygen-ion conducting CFCs (O–CFCs or O–SOFCs), the specific working process of the electrode is as shown in Fig. 1 (on the left): on the cathode side, the surface of the cathode adsorbs oxygen, due to the catalytic effect of the cathode, and the oxygen (O_2_) gains electrons and becomes oxygen ions (O^2−^). Under the effect of oxygen partial pressure, the O^2−^ moves from the cathode through the electrolyte to the interface between the electrolyte and the anode. On the surface of the anode, fuel gas such as hydrogen is adsorbed and undergoes an oxidation reaction to form H^+^, which then reacts with the O^2−^ transported by the electrolyte to generate water and release electrons. The released electrons are transferred from the anode to the cathode through an external circuit and generate an electric current. Thus, electrical energy is continuously outputted.

For proton-conducting CFCs (PCFCs) as shown in Fig. 1 (on the right), the fuel gas (such as hydrogen) undergoes a hydrogen oxidation reaction at the anode, generating protons and electrons. The generated protons migrate to the cathode through the dense proton-conducting electrolyte between the anode and the cathode. The electrons are transferred through the external circuit to the cathode, where they undergo an oxygen reduction reaction (ORR) with oxygen and protons to form water. From the comparison between O–CFCs and PCFCs, it can be seen that PCFCs generate water on the cathode side, avoiding the dilution of fuel gas and thereby improving fuel utilization.

CFCs can generally be classified into three types based on their operating temperatures and the nature of the electrolytes: high-temperature CFCs (800–1000 °C, HT-CFCs), intermediate-temperature CFCs (600–800 °C, IT-CFCs), and low-temperature CFCs (400–600 °C, LT-CFCs).^4–6,11,13^ Due to the fact that the ceramic electrolyte and electrode used in CFCs require a thermal activation process, a relatively high operating temperature is needed to achieve high ionic conductivity (rapid charge transfer) and electrode reaction kinetics. The CFCs operate in high-temperature environments, which put forward higher requirements for the selection of cell materials, thereby increasing the manufacturing cost of the system. Therefore, lowering the operating temperature of CFCs can expand the range of material choices, effectively reduce the manufacturing and operating costs and improve overall system dependability and lifespan of CFCs, thus promoting their commercial development.

However, as the operating temperature drops, the ion mobility in the ceramic electrolyte and the reaction kinetics of the electrodes will significantly decrease, leading to a decline in the ionic conductivity of the ceramic electrolyte and the electrochemical activity of the CFC electrode. This, in turn, significantly increases the polarization resistances of the electrolyte and the electrode, thereby reducing the CFC performance. There are still huge challenges in achieving rapid ionic conduction at low operating temperatures while maintaining the high reaction kinetics and stability of the electrode. Therefore, designing and developing electrolytes with high ionic conductivity at low operating temperatures and electrode materials with high catalytic activity are crucial for improving the performance of LT-CFCs.^4–11^

Faced with these challenges, researchers have proposed many methods to design and develop new CFC materials (electrolyte, cathode and anode), to improve the performance of materials and LT-CFCs.^4–11^ Especially in recent years, strategies such as machine learning, theoretical calculation and high entropy have developed, which have effectively promoted the improvement of CFC material performance and their applications in LT-CFCs. For example, Wang *et al.* introduced a machine learning-based approach to expedite the screening and design of electrode materials, leading to the successful creation of high-performance air electrodes. This study establishes a new framework for developing air electrodes in protonic ceramic cells and designing multi-conductive oxides.^15^ Lei *et al.* employed density functional theory (DFT) to systematically investigate, for the first time, the enhancement mechanism of proton migration at the interface of yttrium-doped barium zirconate matrix proton conductors induced by molten carbonate, and clarified the microscopic process by which carbonate ions reduce the proton migration barrier. This work provides theoretical support for the design of high-performance composite electrolytes for LT-CFCs.^16^ Liu *et al.* developed a high-entropy Pr_1/6_La_1/6_Nd_1/6_Ba_1/6_Sr_1/6_Ca_1/6_CoO_3−*δ*_ bifunctional air electrode for proton ceramic cells and a peak power density (PPD) of 1.21 W cm^−2^ was reached for the fuel cell, achieving a simultaneous improvement in the dual-function catalytic activity and structural stability.^17^ Such examples represent only a fraction of numerous outstanding achievements, yet their design principles offer invaluable insights for the development of new LT-CFC materials.

In recent years, several high-quality reviews have summarized progress in LT-CFCs from diverse perspectives,^3,5,6,8,18^ including thin-film CFCs, individual component materials, and specialized fabrication techniques for CFC-related materials. However, most of these reviews focus primarily on the organization and induction of previous studies, with insufficient attention devoted to emerging materials design strategies such as machine learning (ML), DFT calculations, and high-entropy approaches. Therefore, a comprehensive review that systematically covers materials design and applications across the entire LT-SOFC system is urgently needed.

In contrast, this review not only outlines the state-of-the-art advances in the field but also provides in-depth experimental and theoretical analyses to address the remaining critical challenges. The precise tuning mechanism provides novel insights and original perspectives on materials design and performance optimization.

In this review paper, we focus on new advances in design and application of key materials of LT-CFCs below 600 °C in the recent decade, including cathode, electrolyte and anode materials, and look forward to the challenges in the design of LT-CFC electrolytes and electrode materials in the future. Based on a comprehensive overview of the current development status of LT-CFC materials, we have identified eight design strategies aimed at reducing the operating temperature of CFCs, enhancing their performance, and improving their long-term stability. Through in-depth analysis of these design strategies, we explore the purpose and characteristics of different design strategies in the development and design of LT-CFC materials, providing effective guidance for the further design of LT-CFCs. This review is divided into four major sections: (i) the origin, advantages, and necessity of LT-CFC development are briefly introduced in the Introduction section. (ii) Overview of progress in key materials for LT-CFCs. (iii) The design strategies and application of key materials of LT-CFCs are addressed and discussed, including ML, DFT calculations, high-entropy strategies, defect engineering, mechanical mixing, impregnation strategy, self-assembly, and surface reconstruction. (iv) The challenges and prospects of key materials of LT-CFCs in the future are summarized.

## 2. Overview of progress in key materials for LT-CFCs

Reducing the operating temperature of CFCs to below 600 °C is a great challenge, as it requires the electrolytes to have high ionic conductivity and the electrodes to have high catalytic activity and stability at lower temperatures. This section briefly presents an overview of significant advancements in key materials for LT-CFCs over the past decade, covering cathodes, electrolytes, anodes, and innovative material design strategies. Given the extensive research in this field, we have selectively highlighted representative examples that demonstrate exceptional performance at low temperatures to illustrate state-of-the-art developments. For more comprehensive information, readers are referred to previous authoritative review articles.^3,5,6,18–20^

### 2.1 Cathode materials

Perovskite oxides have emerged as highly promising cathode candidates for LT-CFCs, owing to their exceptional electrocatalytic activity, flexible compositional tunability, and robust structural stability. The commonly reported perovskite phase structures can be classified into three categories: single-phase perovskite (ABO_3−*δ*_), double perovskite (AA′BB′O_6−*δ*_), and Ruddlesden–Popper (R–P) perovskite (A_*n*+1_B_*n*_O_3*n*+1+*δ*_). Depending on their distinct phase structures and ionic configurations, these materials exhibit substantial differences in oxygen ion, electronic, and proton conductivities. Accordingly, both single-phase materials and multiphase composites have been extensively investigated for cathode applications. To satisfy the practical application requirements of cathode materials for LT-CFCs, three critical performance metrics must be prioritized: electrocatalytic activity, long-term operational durability, and thermomechanical compatibility with the electrolyte. In particular, lowering the operating temperature below 600 °C imposes more stringent demands on the electrocatalytic activity of cathode materials, as the kinetics of the ORR at the cathode become drastically sluggish at reduced temperatures. This section summarizes the research progress and key challenges of perovskite-based cathode materials, with a specific focus on the three core aspects mentioned above: catalytic activity, stability, and thermomechanical compatibility.

#### 2.1.1 Catalytic activity

Owing to the fact that the activation energy required for oxygen ion migration is considerably higher than that for proton migration under low-temperature conditions, enhancing the proton conductivity of perovskite oxides serves as a highly effective approach to boost their electrocatalytic activity at reduced operating temperatures. Proton conductivity can be readily introduced into conventional cathode materials with an intrinsic nature of mixed oxygen ion-electronic conductors (MIECs) *via* rational engineering strategies, most typically elemental doping. This endows the cathode with a higher density of electrochemically active sites, and more critically, extends the three-phase boundary (TPB) for the ORR from the narrow electrode–electrolyte interface to the full bulk of the cathode. Rational regulation of perovskite oxides, including the tailoring of the chemical composition, microstructure and morphology, and crystal phase structure, enables the simultaneous optimization of electronic, oxygen ion and proton conductivities as well as surface catalytic properties, thereby yielding a dramatic enhancement in ORR electrocatalytic performance.

Conventional perovskite materials, La_0.6_Sr_0.4_Co_0.2_Fe_0.8_O_3−*δ*_ (LSCF) and Ba_0.5_Sr_0.5_Co_0.8_Fe_0.2_O_3−*δ*_ (BSCF), possess exceptional mixed oxygen ion and electronic conductivity, and have been extensively investigated as pioneering cathode candidates. However, their inferior hydration capability at low temperatures severely hinders the development of proton conductivity, resulting in unsatisfactory electrocatalytic performance.^21^ Accordingly, extensive research efforts have been devoted to enhancing the proton conductivity of these conventional cathode materials. In 2014, Poetzsch *et al.* reported Ba_0.5_Sr_0.5_Fe_0.8_Zn_0.2_O_3−*δ*_ (BSFZ) synthesized *via* the substitution of Co in BSCF with Zn, and first demonstrated the triple H^+^/O^2−^/e^−^ conducting properties of this material, though its proton conductivity remained to be further improved.^22^ Zohourian *et al.* systematically investigated the (Ba,Sr,La)(Fe,Co,Zn,Y)O_3−*δ*_ perovskite system, and found that Ba_0.95_La_0.05_Fe_0.8_Zn_0.2_O_3−*δ*_ (BLFZ) exhibited the highest proton uptake capacity.^23^ In addition to A-site and B-site doping in single-phase perovskites, anion-site (O-site) doping has also been proven to be an effective strategy to boost the electrocatalytic activity of electrode materials. Ni *et al.* doped F^−^ and Cl^−^ into the O-site of the BSCF lattice, which significantly reduced the formation energy of proton defects and enhanced proton migration kinetics.^24^ Shang *et al.* demonstrated that BaCo_0.4_Fe_0.4_Zr_0.2_O_3−*δ*_ (BCFZ), formed by Zr doping into BaCo_0.4_Fe_0.6_O_3−*δ*_, delivered markedly enhanced hydration capability and electrocatalytic activity.^25^ To further improve the proton conductivity of BCFZ, Duan *et al.* rationally designed and synthesized a series of BaCo_0.4_Fe_0.4_Zr_0.2−*x*_Y_*x*_O_3−*δ*_ (BCFZY_*x*_) materials *via* partial substitution of high-valence Zr^4+^ with low-valence Y^3+^. Among them, BaCo_0.4_Fe_0.4_Zr_0.1_Y_0.1_O_3−*δ*_ (BCFZY) exhibited the optimal electrocatalytic activity.^26^ As a representative benchmark single-phase perovskite, extensive modification studies based on the BCFZY parent material have been reported. For example, Liang *et al.* demonstrated that B-site doping of Ni^2+^ and Mg^2+^ into BCFZY can effectively enhance the oxygen ion and proton conductivity of the material.^27,28^ Xu *et al.* found that Zn^2+^ doping into BCFZY also markedly boosted the ORR and hydration kinetics of the material.^29^

In addition to single-phase perovskites, double perovskites have also been widely exploited as cathode materials for LT-CFCs, owing to their abundant oxygen vacancies and superior oxide ion transport capability. Among them, the most widely investigated double perovskite electrode material is PrBa_0.5_Sr_0.5_Co_1.5_Fe_0.5_O_5+*δ*_ (PBSCF).^30^ Choi *et al.* demonstrated that PBSCF exhibits exceptional electrocatalytic activity in humidified atmospheres, along with excellent hydration capability and good chemical compatibility with electrolyte materials.^31^ Thermogravimetric (TG) measurements conducted in dry and humid air confirmed the excellent hydration capability of PBSCF, which is attributed to its low hydration enthalpy.^32^ Seong *et al.* investigated the migration kinetics of proton defects during hydration *via* the isotope exchange diffusion profile (IEDP) technique. The results revealed that its proton surface exchange coefficient and bulk diffusion coefficient are considerably higher than those of most state-of-the-art triple-conducting cathode materials.^33^ Besides Co-based double perovskites, Fe-based double perovskite materials have also been extensively explored as cathode materials. For instance, the cobalt-free Fe-based double perovskite SmBaFe_1.9_Zn_0.1_O_5+*δ*_ (SBFZ) was fabricated *via* the introduction of Zn^2+^ into SmBaFe_2_O_5+*δ*_, which induces lattice distortion and drives the spin-state transition of Fe^3+^ from the high-spin to the low-spin state, thus enhancing Fe–O covalency, promoting oxygen vacancy ordering, boosting proton hydration, and ultimately improving ORR kinetics.^34^

R–P type perovskites have also been employed as cathode materials for LT-CFCs. This class of perovskites consists of alternating perovskite layers and rock-salt layers, and this unique layered structure enables anisotropic transport of oxygen ions and protons, which effectively accelerates ORR kinetics at reduced temperatures. Grimaud *et al.* conducted the first systematic investigation on the hydration and proton transport properties of Pr_2−*x*_Sr_*x*_NiO_4+*δ*_, and confirmed its remarkable hydration capability with proton concentration increasing with increasing water partial pressure, laying a fundamental foundation for the application of R–P perovskites in CFCs.^35^ Yang *et al.* compared the electrochemical performance of La_1.2_Sr_0.8_NiO_4+*δ*_ and Pr_1.2_Sr_0.8_NiO_4+*δ*_, and found that La_1.2_Sr_0.8_NiO_4+*δ*_ delivered superior electrochemical performance. This was attributed to the lower electronegativity of La^3+^ compared with Pr^3+^.^36^ Miao *et al.* doped Fe ions into La_1.2_Sr_0.8_NiO_4+*δ*_ to fabricate La_1.2_Sr_0.8_Ni_0.6_Fe_0.4_O_4+*δ*_, where Fe doping markedly enhanced the oxygen transport properties.^37^ Matvejeff *et al.* reported that Sr_3_Fe_2_O_3−*δ*_ can form hydrated derivatives in humid air, and Lu *et al.* pioneered the application of Sr_3_Fe_2_O_3−*δ*_ as a cathode for CFCs.^38,39^ Theoretical calculations revealed that this material exhibits low proton formation and migration energies, yet it suffers from insufficient chemical stability in humidified atmospheres. Yu *et al.* fabricated Sr_2.8_Fe_1.8_Nb_0.2_O_7−*δ*_ (D-SFN) *via* the introduction of A-site deficiency and B-site Nb doping. The doping of high-valence Nb ions suppressed the formation of the Sr_3_Fe_2_(OH)_12_ secondary phase, while Sr vacancies introduced additional abundant oxygen vacancies into the perovskite lattice, resulting in a significant enhancement in both electrocatalytic activity and operational stability.^40^

Despite the encouraging electrocatalytic performance of some single-phase cathode materials, it remains challenging to simultaneously achieve high electronic, oxygen ion, and proton conductivities in a single-phase material. Fabricating multiphase composite electrode materials has been recognized as an effective strategy to further boost the electrocatalytic activity of cathodes. Initially, researchers predominantly adopted the mechanical mixing strategy, where two or more perovskite materials were blended *via* mechanical ball milling to enhance the electrocatalytic activity of the resulting composites. For instance, conventional cathode materials (LSCF and BSCF) were composited with proton-conducting electrolytes to boost the low-temperature electrocatalytic activity of the composite electrodes, with representative systems including LSCF-BaCe_0.9_Yb_0.1_O_3−*δ*_, LSCF-BaCe_0.9_Y_0.1_O_3−*δ*_, La_0.6_Sr_0.4_Co_*x*_Fe_1−*x*_O_3−*δ*_-BaZr_0.8_Yb_0.2_O_3−*δ*_, BSCF-BaZr_0.1_Ce_0.7_Y_0.2_O_3−*δ*_ (BZCY), and BSCF-BaZr_0.1_Ce_0.7_Y_0.1_Yb_0.1_O_3−*δ*_ (BZCYYb).^41–45^ Notably, composite electrodes fabricated *via* mechanical mixing generally suffer from several intrinsic drawbacks, including inhomogeneous phase distribution, a high thermal expansion coefficient (TEC), and limited interfacial contact area between different phases, which severely restrict the further improvement of electrocatalytic activity. Compared with the conventional mechanical mixing method, the infiltration method enables the uniform dispersion of nanocatalysts onto a porous perovskite scaffold to form composite cathodes. These surface nanoparticles exhibit high electrocatalytic activity and stability, while significantly increasing the specific surface area of the porous scaffold and thus greatly enriching the electrochemically active sites for the electrode reaction. Duan *et al.* infiltrated BCFZY into a porous BaCe_0.6_Zr_0.3_Y_0.1_O_3−*δ*_ scaffold, and the obtained composite cathode delivered significantly enhanced electrocatalytic activity.^26^ Similarly, infiltration of Pr_0.5_Ba_0.5_CoO_3−*δ*_ onto the LSCF surface also effectively boosted the ORR activity of the electrode.^46^ Lee *et al.* infiltrated NiO nanoparticles onto the surface of the BCFZY electrode, achieving enhanced electrocatalytic activity. The NiO nanoparticles can promote the adsorption and rapid dissociation of oxygen molecules, while increasing the number of electrochemically active sites, thus significantly reducing the polarization resistance of the electrode.^47^ However, it is worth noting that the infiltration strategy still cannot guarantee the uniform distribution of nanoparticles on the perovskite scaffold, and the repeated and tedious infiltration procedures significantly increase the time and economic cost of material fabrication. The *in situ* exsolution strategy can effectively address the aforementioned limitations. Nanoparticles exsolved *in situ* from the perovskite parent lattice can be uniformly distributed on the scaffold and form a tight metallurgical bonding with the support. This unique anchoring effect can effectively inhibit the agglomeration of nanoparticles, while significantly enhancing the coking resistance of the electrode. Therefore, developing composite electrode materials with nanoparticles that can be stably exsolved from the perovskite substrate in humid air is a highly promising research direction. For instance, BaCoO_3−*δ*_ (BCO) nanoparticles can be *in situ* exsolved on the surface of PrBa_0.8_Ca_0.2_Co_2_O_5+*δ*_ (PBCC) *via* cation exchange in humid air. The as-formed BCO–PBCC composite cathode exhibits exceptional electrochemical performance, which is attributed to the rapid water dissociation on the BCO nanoparticles and the efficient oxygen desorption on the PBCC host.^48^ A similar BCO exsolution phenomenon was also observed by Niu *et al.* in LSCF-coated Pr_1−*x*_Ba_*x*_CoO_3−*δ*_ electrodes.^46^ Similarly, the double perovskite PrSrCo_1.8_Nb_0.2_O_6−*δ*_ can *in situ* exsolve SrCo_0.5_Nb_0.5_O_3−*δ*_ (SCN) nanoparticles in air. This process enhances the surface oxygen exchange kinetics, lowers the dissociation energy barriers of H_2_O and O_2_, and thus significantly boosts the ORR electrocatalytic activity.^49^

#### 2.1.2 Stability

Operational stability is one of the critical performance metrics for evaluating cathode candidates for LT-CFCs. Specifically, cathode materials must exhibit excellent chemical stability in humid air and CO_2_-containing atmospheres. Meanwhile, the A-site of perovskite oxides commonly accommodates alkaline earth metal cations such as Ba^2+^ and Sr^2+^. These cations are prone to surface segregation under high-temperature operating conditions, which severely deteriorates the structural stability of the perovskite lattice. Accordingly, suppressing cation segregation *via* rational modification strategies has been proven to be an effective approach to improve the long-term stability of perovskite cathode materials. Among these strategies, high-valence cation doping stands out as a facile and efficient method: the incorporation of high-valence cations such as Zr^4+^, Hf^4+^, and Nb^5+^ into the cathode lattice can markedly enhance the structural and operational stability of the cathode. Tsvetkov *et al.* systematically investigated the effects of Hf^4+^, Zr^4+^, Ti^4+^, Nb^5+^, and Al^3+^ doping on the stability of the perovskite La_0.8_Sr_0.2_CoO_3−*δ*_. The results demonstrated that these high-valence cations significantly improved the stability of the material.^50^

Recently, high-entropy perovskites (HEPs) have been developed as a promising class of cathode materials, where the high configurational entropy of the perovskite lattice is leveraged to enhance thermodynamic stability. Compared with conventional perovskites, HEPs exhibit severe lattice distortion originating from the substitution of multiple elements with distinct ionic radii.^51^ Such lattice distortion can effectively suppress the segregation of metal cations, thus boosting the structural stability of the perovskite framework. Yang *et al.* synthesized BaCo_0.2_Zn_0.2_Ga_0.2_Zr_0.2_Y_0.2_O_3−*δ*_ (BCZGZY) as a cathode for CFCs. Compared with the parent BaCoO_3−*δ*_, high-entropy BCZGZY not only delivers significantly enhanced structural stability, but also exhibits a reduced TEC and improved interfacial adhesion between the electrode and electrolyte.^52^ Nevertheless, B-site high-entropy engineering, while effectively enhancing the stability of electrode materials, inevitably compromises the electrocatalytic activity of the material. In contrast, A-site high-entropy engineering can improve the structural stability of perovskites while preserving their intrinsic electrocatalytic activity. Li *et al.* adopted A-site high-entropy engineering to synthesize Pr_0.2_Nd_0.2_Sm_0.2_Ba_0.2_Sr_0.2_CoO_3−*δ*_ (PNSBSC) using Sm_0.6_Sr_0.4_CoO_3−*δ*_ as the parent material. Compared with the parent material, PNSBSC exhibited significantly improved stability, which was further verified in large-area single cells.^53^

In addition to the high-entropy strategy, the nanoparticle infiltration approach has also been proven to effectively enhance the operational stability of electrode materials. Pei *et al.* coated the nanocatalyst Pr_0.1_Ce_0.9_O_2+*δ*_ (PCO) onto the surface of the highly active perovskite PrBaCo_2_O_5+*δ*_ (PBC) to fabricate the PCO–PBC composite electrode. This composite electrode exhibited significantly enhanced long-term durability, as the PCO coating on the PBC surface effectively suppressed the segregation of Ba cations.^54^ To enhance the tolerance of BaCe_0.5_Pr_0.3_Y_0.2_O_3−*δ*_ against H_2_O and CO_2_, PrNi_0.5_Co_0.5_O_3_ was infiltrated onto its surface. The PrNi_0.5_Co_0.5_O_3_ coating can effectively prevent the chemical reaction between alkaline earth metal cations and H_2_O/CO_2_, and markedly improve the stability of the composite electrode.^55^

#### 2.1.3 Thermal compatibility

The thermodynamic instability of CFCs is primarily rooted in the large internal strain gradient induced by the mismatch in the TEC between the electrolyte and cathode materials.^56^ The TEC of conventional ceramic electrolytes typically lies in the range of 10 × 10^−6^ K^−1^–12 × 10^−6^ K^−1^, while cobalt-based perovskite oxides with high electrocatalytic activity have a TEC as high as 18 × 10^−6^ K^−1^–25 × 10^−6^ K^−1^. The intrinsically elevated TEC of these materials is directly correlated with the spin-state transition of Co ions and the low bonding energy of Co–O bonds.^57^ On this basis, two main pathways are currently adopted to optimize the interfacial thermodynamic compatibility: the first is elemental doping modification, which regulates the TEC by suppressing the oxidation state/spin-state transition of metal ions during the polarization process; the second is the fabrication of composite electrodes, where the overall TEC of the system is taken as the weighted average of the TEC of each component through physical compounding of multiple constituents, thereby alleviating the internal strain gradient and improving the thermodynamic stability of the cell.^58^

Numerous studies have demonstrated that incorporating high-valence metal cations such as Nb^5+^, Zr^4+^, Ta^5+^ and Mo^6+^ into the B-site of perovskite materials can markedly enhance the structural stability and reduce the TEC.^59^ Huang *et al.* found that increasing the Nb doping content in Ba_0.5_Sr_0.5_Co_0.8−*x*_Fe_0.2_Nb_*x*_O_3−*δ*_ reduced the TEC of the material from 21.74 × 10^−6^ K^−1^ to 18.74 × 10^−6^ K^−1^.^60^ Xu *et al.* doped high-valence Mo^6+^ into PBC, which also induced a reduction in the TEC.^61^ This modification effectively mitigates the thermal mismatch between the cathode and commonly used electrolytes. Similarly, partial substitution of Co ions at the B-site of perovskites with transition metal cations can also achieve an effective reduction in the TEC. Jin *et al.* fabricated the PrBaCo_2/3_Fe_2/3_Mn_1/2_O_5+*δ*_ electrode material *via* partial substitution of Co with Fe and Mn. Compared with the parent material, the reduced B-site Co content significantly lowered the TEC of the material.^62^ Strengthening the bonding energy between B-site metal cations and oxygen, or reducing the B-site Co content, can effectively reduce the TEC of perovskite materials.

Although high-valence metal cation doping can reduce the TEC, most doping modification strategies only achieve a slight reduction in the TEC of Co-based electrodes, failing to fundamentally resolve the thermodynamic mismatch between the electrode and electrolyte. Since the discovery of the negative thermal expansion (NTE) behavior of ZrW_2_O_8_ in 1996, the construction of composite cathodes using high electrocatalytically active materials and negative TEC materials has received extensive attention as a promising strategy for TEC reduction.^63^ Lin *et al.* composited the NTE material Y_2_Mo_3_O_12_ (YMO) with BSCF, successfully reducing the TEC of the composite electrode. This not only improved the thermal matching between the cathode and electrolyte, but also enhanced the output performance of the single cell. Simulations and experimental results confirmed that YMO can relieve the stress at the electrode–electrolyte contact interface and prevent cathode delamination.^64^ Wang *et al.* incorporated the NTE material NdMnO_3−*δ*_ (NM) into PBC, which drastically reduced the average TEC from 22.3 × 10^−6^ K^−1^ for pristine PBC to 12.2 × 10^−6^ K^−1^ for the PBC-NM composite.^65^ Similarly, Lu *et al.* composited NM with Ba_0.5_Sr_0.5_FeO_3−*δ*_ (BSF), achieving a significant reduction in the TEC of the BSF-NM composite electrode. When fabricated into single cells, the pristine BSF electrode exhibited obvious cracking and delamination, while the BSF-NM electrode maintained tight interfacial adhesion to the electrolyte. This further demonstrates that the reduced TEC markedly improved the thermal matching between the cathode and electrolyte.^66^

### 2.2 Electrolyte materials

Electrolyte materials serve as a core component of CFCs, fulfilling the critical functions of oxygen-ion/proton conduction and fuel-oxidant separation, which directly determine cell performance. The desired electrolyte must exhibit high ion conductivity, negligible electronic conductivity, outstanding thermochemical stability, and compatible thermal expansion with electrode materials. In the context of LT-CFCs, electrolyte research is actively advancing toward lower operating temperatures, enhanced ionic conductivity, and improved structural stability, aiming to reduce operational temperature, improve efficiency and lifespan, and promote the application of electrolyte materials in LT-CFCs. Key strategies include low-temperature adaptation of conventional electrolytes, performance breakthroughs in novel single-phase materials, and the development of composite and dual/triple-ion conducting electrolytes. This section briefly outlines recent progress and persistent challenges of electrolyte materials in three major categories: oxygen-ion conducting electrolytes, proton-conducting electrolytes, and composite/multi-ionic conducting electrolytes.

#### 2.2.1 Oxygen ion-conducting electrolytes

The research progress on electrolyte materials for LT-SOFCs in recent years has focused on enhancing ionic conductivity, chemical stability and interfacial compatibility at low temperatures. 8 mol %Y_2_O_3_-stabilized ZrO_2_ (YSZ) is the most commonly used oxide-ion-conducting electrolyte for traditional high-temperature CFCs. However, its conductivity sharply reduces from 0.1 S cm^−1^ at 1000 °C to 0.0011 S cm^−1^ at 600 °C.^67,68^ Therefore, when YSZ is used as the electrolyte, its thickness must be thin enough to minimize the ohmic resistance. Lee *et al.* reported a CFC comprising a 1.4 µm thick YSZ electrolyte film and a 400 nm Ce_0.9_Gd_0.1_O_2−*δ*_ (GDC) interlayer and a LSCF-YSZ composite cathode with a nanofibrous and porous structure fabricated by the magnetron sputtering method for all CFC components. The PPD reached 1.7 W cm^−2^ at 600 °C. This shows the feasibility of this method for CFC fabrication.^69^ For more information about electrolyte thin film CFCs, please see the review paper by Zhang *et al.*^8^

CeO_2_-based electrolytes have higher oxygen-ion conductivity at low temperatures compared with YSZ. However, they also face problems such as electronic conductivity caused by the reduction of Ce^4+^ to Ce^3+^ and insufficient ionic conductivity at low temperatures, especially the excessively high grain boundary resistance at low temperatures. Therefore, it is highly necessary to enhance the ion conductivity of existing CeO_2_-based materials or develop new electrolyte materials.^70^ Chen *et al.* reported a new ion conduction mechanism of GDC electrolyte with a nanocrystalline structure, and the ion conductivity reached 0.37 S cm^−1^ at 550 °C. The single cell using this nanocrystalline GDC electrolyte delivered a PPD of 591.8 mW cm^−2^ at 550 °C, which was 3.5 times higher compared to the cell using the dense GDC electrolyte after sintering at 1550 °C, showing potential for applications in LT-CFCs.^71^

Doping with low-valent metal cations can enhance the oxygen-ion conductivity of CeO_2_ materials. Compared to singly doped CeO_2_, co-doped CeO_2_ electrolytes lead to enhanced oxygen-ion conductivity with negligible electronic conductivity at relatively lower temperatures. This is due to the inhibition of the ordering of oxygen vacancies resulting in a lower activation energy for co-doping CeO_2_ than that for single-doping CeO_2_.^72,73^ The researchers reported a doubly-doped Gd_0.14_Pr_0.06_Ce_0.8_O_1.90_ electrolyte; its conductivity is 0.0301 S cm^−1^ at 600 °C, and this value is 0.0125 S cm^−1^ at the same temperature for the parent Gd_0.2_Ce_0.8_O_1.90_ material.^74^ In another study, researchers reported a triple-doped ceria electrolyte Ce_0.76_La_0.08_Pr_0.08_Sm_0.08_O_2−*δ*_, exhibiting an ionic conductivity of 0.043 S cm^−1^ at 600 °C with a low activation energy of 0.76 eV.^75^ Its electrical conductivity is also higher than that of the parent Ce_0.8_Sm_0.2_O_2−*δ*_ samples (5.00 × 10^−3^–1.37 × 10^−2^ S cm^−1^ at 600 °C).^76^ This shows the advantage in the conductivity enhancement of doped ceria using the co-doping strategy. It should be noted that while enhancing the oxygen-ion conductivity of the doped CeO_2_ electrolyte, inhibiting the reduction of Ce^4+^ to reduce electronic conductivity remains a challenging task. In addition, although these novel electrolyte materials exhibit high ionic conductivity at lower temperatures, their applications in LT-CFCs are still relatively limited.

LaGaO_3_-based electrolyte (*i.e.*, La_0.9_Sr_0.1_Ga_0.8_Mg_0.2_O_3−*δ*_, LSGM) is an important kind of oxygen-ion-conducting electrolyte. However, LSGM suffers from chemical stability problems during material preparation processing or long-term operation, for example, reacting with the Ni component in a Ni-based anode and volatility of Ga at high sintering temperatures. Therefore, a buffer layer of doped CeO_2_ has to be used to avoid the interface diffusion reaction.^77,78^ Wang *et al.* prepared a Sm_0.2_Ce_0.8_O_2−*δ*_/La_0.8_Sr_0.2_Ga_0.8_Mg_0.2_O_3−*δ*_/Sm_0.2_Ce_0.8_O_2−*δ*_ sandwiched electrolyte film using the tape casting process. The cell PPD was 260 mW cm^−2^ at 600 °C, showing superior cell performance to cells with a similar structure.^79^ Chen *et al.* successfully prepared a high-density GDC-LSGM composite electrolyte layer using plasma spraying technology. The continuously distributed LSGM effectively blocked the electron-conducting channels caused by cerium cation reduction from Ce^4+^ to Ce^3+^, thereby enhancing the open-circuit voltage (OCV) and cell performance. The corresponding CFC achieved an OCV of 1.03 V and a PPD of 371 mW cm^−2^ at 600 °C.^80^ Improving the chemical stability of LSGM electrolyte, especially reactions with nickel in CFC components remains a problem that cannot be ignored. Searching for novel anode materials may be an alternative solution to overcome the problem of Ni interface diffusion from a Ni-based anode.

#### 2.2.2 Proton-conducting electrolytes

Proton-conducting perovskite oxides have shown obvious advantages as an electrolyte for use in LT-CFCs due to their higher ionic conductivity and lower activation energy compared to typical oxygen-ion conducting electrolytes.^81,82^ BaCeO_3−*δ*_ and BaZrO_3−*δ*_-based perovskites are the two most commonly used proton-conducting electrolytes in low-temperature proton ceramic fuel cells (LT-PCFCs). The former exhibits high electrical conductivity but poor stability, while the latter has high stability but low electrical conductivity. Therefore, the research mainly focuses on doping modification, solid solution optimization, and the development of new proton-conducting electrolytes.^5,12,18^ The performance of electrolytes has been optimized through doping and non-stoichiometric regulation. Hyodo *et al.* investigated the properties of the Sc-doped BaZrO_3−*δ*_ (BaZr_1−*x*_Sc_*x*_O_3−*δ*_; *x* = 0.2 and 0.6) electrolytes. The 60%-Sc-doped BaZrO_3−*δ*_ (BaZr_0.4_Sc_0.6_O_3−*δ*_) sample was found to have a high proton conductivity of 0.01 S cm^−1^ at 396–534 °C. They revealed that high-level Sc doping in BaZrO_3−*δ*_ was the key to achieving high proton conductivity and chemical stability. The core function of high Sc doping is to simultaneously enhance proton concentration, bulk diffusivity, and grain-boundary conductivity and promote grain growth, providing a new strategy for designing high-performance proton-conducting electrolyte materials.^83^

The development of new proton-conducting electrolytes with improved performance is of great significance to LT-CFCs. Wang *et al.* developed new indium-doped BaHfO_3−*δ*_ (BaHf_1−*x*_In_*x*_O_3−*δ*_) electrolytes. The optimal composition BaHf_0.85_In_0.15_O_2.925_ exhibited the highest conductivity (0.33 S cm^−1^ at 550 °C) and cell PPD of 956 mW cm^−2^ at 550 °C.^84^ Guo and He successfully developed a high-entropy BaSn_0.16_Zr_0.24_Ce_0.35_Y_0.1_Yb_0.1_Dy_0.05_O_3−*δ*_ (BSZCYYbD) proton-conducting electrolyte and applied it to PCFCs for the first time, showing good electrical and electrochemical properties and structural stability.^85^ Current research on proton-conducting electrolytes still needs to address issues such as insufficient protonic conductivity at low temperatures, chemical instability in a CO_2_/H_2_O atmosphere, barium evaporation during sintering, and poor compatibility at the electrode–electrolyte interface.

#### 2.2.3 Composite and dual- and triple-conducting electrolytes

Composite electrolytes are a promising alternative electrolyte material, which can to some extent make up for the deficiencies of single-component electrolytes in terms of conductivity and stability.^86^ Zhang *et al.* reported Sm_0.2_Ce_0.8_O_2_–Na_2_CO_3_ (NSDC) nanocomposite electrolyte with different contents of residual carbonates. Their results demonstrated that the optimal 90%Sm_0.2_Ce_0.8_O_2_–10%Na_2_CO_3_ (NSDC9010) nanocomposite exhibited a protonic conductivity of 0.044 S cm^−1^ at 650 °C, which is much higher than that of the classical perovskite proton-conducting electrolytes. Based on the NSDC9010 electrolyte supported CFC, a PPD of 281.5 mW cm^−2^ was achieved at 600 °C.^87^

Dual ion-conducting electrolyte materials exhibit both oxygen ionic and protonic conductivity at the same time. In earlier research, Iwahara reported that BaCe_0.9_Nd_0.1_O_3−*δ*_ is a dual-ion conducting electrolyte and proposed possible applications.^88^ Researchers have developed dual-ion CFCs based on two carriers (oxygen ions and protons), where the two carriers will participate in electrochemical reactions in CFCs. Yang *et al.* reported a dual-ion conducting electrolyte, BZCYYb, whose conductivity was 0.013 S cm^−1^ at 500 °C, which has become one of the most commonly used proton-conducting electrolyte materials. The single cell using anode-supported thin-film BZCYYb electrolyte delivered a PPD of 732 mW cm^−2^ at 600 °C.^89^ The advantage of the dual-ion conductivity of the BZCYYb electrolyte was further substantiated by subsequent research.^90^

Inspired by the dual-ion conducting electrolyte, Ruan *et al.* reported a new (H^+^/O^2−^/Li^+^) triple-ion conducting electrolyte Li_4_Ti_5_O_12_ for LT-CFCs. The results showed that the ionic conductivities of Li_4_Ti_5_O_12_ are 0.05 and 0.45 S cm^−1^ at 450 and 550 °C, respectively. The corresponding single cell delivered a PPD of 600 mW cm^−2^ at 550 °C, which has obvious advantages compared to the single-ion conducting electrolyte.^91^ This work provided a possibility for designing new triple-conducting electrolyte materials for LT-CFCs. However, the precise measurement of conductivity of triple charges (H^+^/O^2−^/Li^+^) is still a challenging task for the design of triple-ion conducting electrolytes.

### 2.3 Anode materials

As the site for fuel oxidation in CFCs, the anode plays a critical role in determining cell performance and lifespan. It must simultaneously meet stringent requirements, including high electronic conductivity, favorable ionic transport capability, excellent catalytic activity, and long-term operational stability.^92,93^ Conventional Ni-YSZ anodes perform well in pure hydrogen fuel at intermediate-to-high temperatures (above 600 °C). However, when the operating temperature is reduced to 300–600 °C, their electrocatalytic activity for hydrogen oxidation, and especially for hydrocarbon fuels and ammonia, decreases drastically. They also suffer from severe challenges such as carbon deposition, sulfur poisoning, and nitridation, leading to significant polarization losses and rapid performance degradation.^94–96^

In recent years, extensive research has been conducted by researchers worldwide to optimize the performance of anode materials and develop novel alternatives. Currently, the most widely investigated anode materials include Ni-based ceramic anodes and perovskite-type anodes.

#### 2.3.1 Ni-based ceramic anodes

Ni-based composite anodes remain a research hotspot in recent years due to their combined high electrical conductivity and catalytic activity. Through strategies such as composite structure optimization, alloying modification, and promoter incorporation, researchers have significantly improved their resistance to carbon deposition, sulfur poisoning, and nitridation, thereby expanding their applicability across diverse fuel systems.

A direct strategy to lower the operating temperature of SOFCs is to employ electrolyte materials with higher ionic conductivity at low temperatures. Compared with conventional oxygen-ion conductors such as YSZ, proton conductors including doped BaCeO_3_ and BaZrO_3_ exhibit lower ionic conduction activation energies, thus exhibiting distinct advantages in the intermediate-to-low temperature range of 400–600 °C. Duan *et al.* used Ni-BZCYYb as the anode and BZCYYb as the electrolyte, achieving a maximum power density of 455 mW cm^−2^ at 500 °C with hydrogen fuel. This pioneering work has greatly contributed to the development of LT-PCFCs.^26^

Using DFT, Liu *et al.* designed and synthesized a Sm-doped CeO_2_-supported Ni–Ru bimetallic catalyst (SCNR). The SCNR catalyst reduces the C–H bond activation energy barrier *via* the Ni–Ru synergistic effect, and promotes H_2_O adsorption and dissociation through oxygen vacancies introduced by Sm doping, significantly enhancing the methane reforming efficiency and the anti-coking performance of the anode. Experiments show that at 600 °C and an extremely low steam-to-carbon ratio (5% H_2_O), the CH_4_-fueled SOFC with SCNR modification delivers outstanding electrochemical performance: a PPD of 361 mW cm^−2^, representing an approximately 89% improvement over the unmodified cell (190 mW cm^−2^). Meanwhile, the cell exhibits stable operation for more than 2250 h at 600 °C, demonstrating exceptional coking resistance and structural stability.^97^

Incorporating promoters such as alkali metals and alkaline earth metals into Ni-based anodes is an effective approach to improve their low-temperature performance and resistance to carbon deposition. Addressing the insufficient catalytic activity of anodes for LT-SOFCs, Lim *et al.* developed a cesium (Cs)-promoted Ni-GDC anode. CsNO_3_ was used as the promoter precursor and introduced into the Ni-GDC anode *via* solution impregnation or powder mixing, decomposing to Cs_2_O or CsOH under SOFC operating conditions. Studies reveal that the Cs promoter facilitates oxygen and water supply on the Ni-GDC surface, boosting anode performance by 76% at 350 °C in 3% humidified hydrogen. At 450 °C in 3% humidified methane fuel, a power density above 50 mW cm^−2^ can be maintained for 15 h without obvious carbon deposition. The mechanism is as follows: the Cs promoter partially oxidizes the Ni catalyst surface to form Ni/NiO interfaces, optimizing the binding energy of hydrogen and hydroxyl species and enhancing the hydrogen oxidation reaction (HOR) activity. Meanwhile, the strong basicity of Cs promotes partial oxidation and steam reforming of methane, suppressing carbon deposition.^98^

#### 2.3.2 Perovskite-type anodes

Perovskite oxides and their derivatives have attracted extensive attention in recent years due to their good structural stability, tunable electron-ion conductivity, and excellent carbon and sulfur tolerance. Catalytic activity and stability of perovskite-based anodes can be further improved through strategies such as A/B-site doping and *in situ* exsolution of metal nanoparticles.

Liang *et al.* doped Ru into the perovskite Pr_0.6_Sr_0.4_(Co_0.2_Fe_0.8_)_0.85_Ru_0.15_O_3−*δ*_ (PSCFR15), leading to the *in situ* exsolution of CoFeRu ternary alloy nanoparticles under a reducing atmosphere. It was found that the introduction of Ru promotes the exsolution of Co and Fe. Ru significantly weakens the binding strength of N atoms on the alloy surface, enabling easier desorption of N_2_ and thus preventing poisoning by nitrogen species, greatly enhancing ammonia decomposition efficiency and anti-deactivation capability. Ammonia decomposition tests show that r-PSCFR15 achieves an ammonia conversion rate of 79% at 550 °C, much higher than that of Ru-free r-PSCF (∼40%).^99^

Using a one-pot self-assembly method combined with *in situ* reduction, Gan *et al.* successfully prepared a La_0.9_Ce_0.1_Ni_0.7_Co_0.15_Fe_0.15_O_3−*δ*_-Sm_0.2_Ce_0.8_O_2_ (LCNCF-SDC) composite anode, which *in situ* reconstructs into a multiphase heterostructure of NiCoFe/CoO/LaO_*x*_/SDC under reducing conditions. Electrochemical measurements show that a single cell supported by a 500 µm-thick SDC-carbonate composite electrolyte delivers peak power densities of 0.94 W cm^−2^ and 0.58 W cm^−2^ at 600 °C and 550 °C, respectively, using methanol as fuel. The superior low-temperature performance and coking resistance are attributed to the synergistic catalysis of multiphase nanoparticles, abundant oxygen vacancies, and the strong interaction between the NiCoFe alloy and the SDC support, providing a new strategy for the design of anodes for direct hydrocarbon-fueled SOFCs.^100^

As demonstrated by the above examples, researchers have developed various material design strategies for different types of anode materials, including novel electrolyte composites, DFT guidance, *in situ* exsolution of nanoparticles, and self-assembly. Numerous review articles have summarized and reported anode materials.^101–103^ However, most of these reviews focus on materials with a single design strategy or a single structure, and do not include the guidance of recently emerging machine learning and density functional theory calculations in anode material design.^104,105^ Therefore, a more comprehensive review on anode design strategies for LT-CFCs is highly desirable.

### 2.4 Material design strategies

From the research progress of the above materials, it can be seen that challenges still exist. Therefore, there is an urgent need to continuously improve the performance of materials. The design objective of LT-CFC materials is to achieve high ionic/electronic conductivity, high catalytic activity, low interface impedance, thermochemical matching and long-term stability at low temperatures. With the passage of time, design concepts are constantly being updated. In recent years, it has been highlighted in the following aspects, but is not limited to these: ML, theoretical calculation, high entropy, exsolution, reconstruction, impregnation, defect engineering and self-assembly.

The ML technique promotes the development of new materials by accelerating material discovery, reducing experimental costs and optimizing material design, which has been applied to predict and discover the performance of materials. By combining ML, theoretical calculations and perovskite compositions, Zhai *et al.* developed an experimentally verified ML screening technique for cathode materials, which can rapidly and effectively screen out highly active cathode materials for reduced temperature CFCs from the vast perovskite compositions, showing the technical advantages of low cost and high development efficiency.^106^

To develop efficient and durable proton-conducting electrolytes, Liu *et al.* systematically investigated the microscopic mechanism of the enhanced proton conduction performance of 4f electrons in Pr-doped BaCeO_3_ by means of DFT and *ab initio* molecular dynamics (AIMD). Beyond decoding the fundamental mechanisms governing proton transport, they conducted a comprehensive assessment of the material's stability as a potential electrolyte for PCFCs. This investigation delivered profound atomic-level guidance for the rational design of next-generation low-temperature PCFC electrolytes.^107^

To solve the problems of intrinsic insufficient ORR activity and element segregation of traditional R–P oxides (such as Ln_2_NiO_4+*δ*_), Yin *et al.* designed a novel high-entropy R–P cathode (La_0.4_Pr_0.4_Nd_0.4_Ba_0.4_Sr_0.4_NiO_4+*x*_, LPNBSN) through the entropy-engineering strategy, achieving a synergistic enhancement in catalytic activity, proton transport capacity and long-term stability. This has set a new performance record for R–P cathodes in PCFCs and provides a new solution for the high performance and practical application of PCFCs.^108^

To address the core issues of carbon deposition and sulfur poisoning faced by CFCs when directly using natural gas, Wang *et al.* developed (Cu, Sm)CeO_2_ anode materials anchored with Cu nanoparticles through *in situ* exsolution for the direct application of natural gas in CFCs. They confirmed that this material exhibits high electrocatalytic activity, excellent anti-carbon deposition/anti-sulfur poisoning performance, and long-term stability, providing an efficient anode solution for LT-CFCs to directly utilize natural gas.^109^

Zhao *et al.* designed and fabricated a BaCe_0.4_Fe_0.4_Co_0.2_O_3−*δ*_ twin-perovskite nanocomposite cathode with triple conductivity by one-pot synthesis for the first time, which consisted of a proton-conducting phase of BaCe_0.85_Fe_0.15_O_3−*δ*_ (cubic) and mixed electron-ion conducting phase of BaCe_0.15_Fe_0.85_O_3−*δ*_ (orthorhombic) with a ratio of about 4 : 1. The cubic-orthorhombic twin-perovskite nanostructure is the core reason for its high performance. The design concept of twin-perovskite nanocomposite cathodes provided a new approach for independently regulating the conductivity of each phase and developing high-performance triple-conducting cathodes for PCFCs.^110^

Wang *et al.* prepared nano-spinel-modified perovskite oxide Nd_0.5_Ba_0.5_Mn_0.7_Co_0.15_Ni_0.15_O_3−*δ*_-(Co_*x*_Ni_*y*_)_3_O_4_ (CNO@NBMCN) by the reversible phase transformation-induced exsolution method for the first time and investigated its performance as an air electrode in protonic ceramic cells. It has achieved a dual breakthrough in high catalytic activity and long-term stability. CNO spinel nanoparticles are stably anchored in the NBMCN matrix in a high-temperature oxidizing atmosphere, solving the stability problem of traditional dissolution electrodes. Meanwhile, the intrinsic mechanism of high-temperature anchoring of nanoparticles was revealed through phase field simulation, providing a new paradigm for the design of heterogeneous air electrodes.^111^

Facing the problems of low catalytic performance and poor stability of direct ammonia ceramic fuel cell (DA-CFC) anodes, Zhang *et al.* developed a re-structured anode surface for DA-CFCs on the Fe-modified Ni-BZCYYb anodes through the solution impregnation technique, which significantly enhanced the catalytic activity, power output and durability of DA-CFCs. It provided important guidance for the design of high-efficiency DA-CFC electrocatalysts.^112^

## 3. Design and applications of key materials for LT-CFCs

### 3.1 Machine learning

In the development of new materials, the trial-and-error experimental screening process is the most traditional method, which is time-consuming and laborious and lacks precision. Although it is possible to use theoretical calculations to screen materials, the significant computational cost makes such calculations potentially prohibitive when screening large numbers of possible compounds. In comparison, ML is a new technology to accelerate the discovery of new materials. Only in the last few years, it has been used in the screening of CFC materials, such as electrodes and electrolytes; therefore, the applied number is still small.

#### 3.1.1 Design and applications of cathode materials

**Fig. 2:**
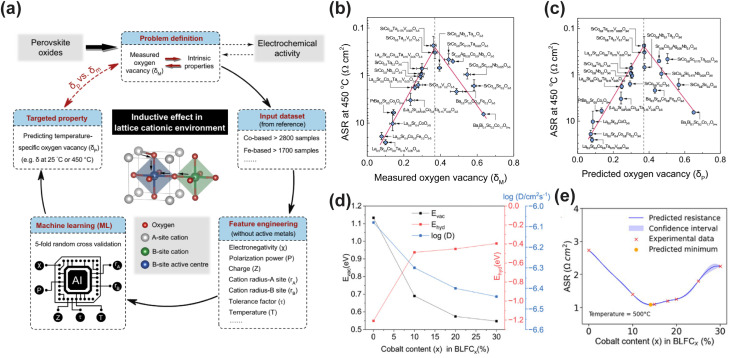
(a) The workflow of machine learning (ML) assisted prediction of the oxygen vacancy concentration of perovskite oxides. The relationship between ORR activity at 450 °C and (b) measured and (c) ML-predicted oxygen vacancy concentration. Reproduced with permission.^113^ Copyright 2024, Springer Nature. (d) Computational predictions of oxygen vacancy formation energy, hydration energy, and diffusivity *D* as a function of Co content. (e) Bayesian optimization of ASR at 500 °C. The blue line represents the Gaussian process regressor model, the shaded region shows the confidence interval, the red x marks experimental measurements, and the orange dot indicates the predicted minimum for the next iteration. Reproduced with permission.^116^ Copyright 2025, Wiley-VCH GmbH.

In recent years, ML-assisted material design has emerged as a powerful approach to accelerate the screening of high-performance CFCs, particularly cathode materials for LT-CFCs. Zhai *et al.* innovatively introduced the ionic Lewis acid strength (ISA) as an effective physical descriptor to quantify the ability of A-site and B-site cations in perovskite oxides to accept electron pairs, thereby establishing an intrinsic correlation with their ORR activity.^106^ The researchers first constructed a dataset containing the area-specific resistance (ASR) of 85 known perovskite materials. After comparing multiple regression models, they found that the artificial neural network (ANN) model performed best in predicting ASR, achieving a mean square error (MSE) of 0.0131 Ω cm^2^ on the test set. Feature importance analysis revealed that B-site ISA (BISA) was the most critical descriptor influencing ORR activity, while the combination of A-site and B-site ISA (AISA + BISA) provided a more comprehensive capture of the activity trend: lower A-site ISA combined with higher B-site ISA synergistically enhanced ORR kinetics. Based on this model, the researchers screened four highly promising cathode materials from 6871 theoretical compositions, such as Sr_0.9_Cs_0.1_Co_0.9_Nb_0.1_O_3_ (SCCN). Experimental results validated the ML predictions. The synthesized materials formed pure perovskite phases and exhibited outstanding electrochemical performance. Notably, the SCCN cathode demonstrated exceptional performance, with an ASR as low as 0.035 Ω cm^2^ at 600 °C. Moreover, an anode-supported single cell based on the SDC electrolyte and the SCCN cathode achieved PPDs of 1.52 W cm^−2^ at 600 °C and 1.19 W cm^−2^ at 550 °C, significantly surpassing most reported low-temperature cathode materials and highlighting its great potential for LT-CFC applications. Similarly, Li *et al.* proposed an ML-based method for predicting the oxygen vacancy concentration in perovskite oxides at different temperatures, guiding the design of LT-CFC cathode materials.^113^ By extracting intrinsic properties of A-site and B-site cations, including the tolerance factor, electronegativity, polarization power, charge, and cation sizes for A-site (*r*_A_) and B-site cations (*r*_B_) as input features, they achieved high-precision predictions of oxygen vacancy concentrations for 235 cobalt-based and 200 iron-based perovskites (Fig. 2a).^114,115^ The study revealed a volcano-type relationship between oxygen vacancy concentration and ORR activity, with the optimal oxygen vacancy concentration increasing with temperature (Fig. 2b and c). Based on these insights, they designed and synthesized two novel perovskite materials SrCo_0.8_Ta_0.15_V_0.05_O_3−*δ*_ (SCTV) and SrCo_0.8_Ta_0.16_W_0.04_O_3−*δ*_ (SCTW). At 450 °C, the ASR values of SCTV and SCTW were 0.165 and 0.265 Ω cm^2^, respectively, significantly outperforming conventional materials such as BSCF and LSCF. When SCTV was applied in anode-supported single cells, the cell PPDs of 1.36, 0.85, and 0.45 W cm^−2^ were achieved at 500, 450, and 400 °C, respectively, demonstrating excellent low-temperature electrocatalytic activity.

ML-assisted material design strategies have been applied for the development of high-performance cathodes for PCFCs, showing strong potential in addressing complex compositional combinations and multi-property trade-offs. Tahir *et al.* proposed an innovative integrated computational and experimental validation strategy to optimize Co-substituted Ba_0.95_La_0.05_FeO_3−*δ*_ (BLF) cathode materials.^116^ Their approach combined a ML potential (M3GNet) with Bayesian optimization (BO) to systematically investigate the effect of Co content on BLF cathode performance.^117^ First, M3GNet was used to screen the thermodynamic stability of Ba_0.95_La_0.05_Fe_1−*x*_Co_*x*_O_3−*δ*_ across the full composition range (*x* = 0 to 100%) and to predict key performance descriptors, including oxygen vacancy formation energy, hydration energy, and the oxygen ion diffusion coefficient. The calculation results indicated that increasing Co content increased the oxygen vacancy concentration but reduced hydration capability and oxygen diffusivity, indicating a clear performance trade-off (Fig. 2d). Based on these results and X-ray diffraction (XRD) validation, the experimental study was narrowed to the single-phase perovskite region with Co content *x* ≤ 30%. To further optimize the Co content for minimizing ASR, a Bayesian optimization strategy was employed, guided by a Gaussian process regressor and the Expected Improvement acquisition function, efficiently navigating the nonlinear relationship between ASR and composition with limited experimental data. Ultimately, BLFC15 (15% Co) was identified as the optimal composition, reducing ASR by 58% compared to undoped BLF at 500 °C (Fig. 2e). In both symmetric cells and anode-supported single cells based on a BZCYYb electrolyte, BLFC15 exhibited excellent electrochemical performance and stability. The single cell achieved a PPD of approximately 640 m W cm^−2^ at 600 °C and remained stable during a 50-h operation at 500 °C, indicating promising practical applicability. It is noteworthy that moderate Co doping increases oxygen vacancy concentration and electronic conductivity, and optimizes the ORR pathway by adjusting the O 2p and Co 3d band centers. However, excessive Co doping reduces hydration capacity and proton conductivity, ultimately degrading performance. Tang *et al.* constructed a predictive model using the eXtreme Gradient Boosting (XGBoost) algorithm, employing hydrated proton concentration (HPC) as a key descriptor to systematically evaluate the proton conductivity of 
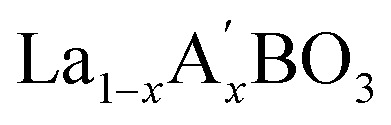
 (A′ = Na, K, Ca, Mg, Ba, Cu, Pr, Sc; B = Co, Mn, Fe) perovskite oxides.^23,118^ The model was trained on a dataset of 784 samples and optimized with 10-fold cross validation, showing high prediction accuracy. SHAP (SHapley Additive exPlanations) analysis revealed the influence of factors such as temperature, water pressure, ionic radius, dopant fraction, and elemental melting point on HPC. Through model prediction, La_0.8_Ba_0.2_CoO_3_ (LBC) was identified as the most promising air electrode material. Experimental validation confirmed that LBC exhibits high HPC at 600 °C. In single cell applications, a PCFC with an LBC electrode achieved PPDs of 1.00 and 0.56 W cm^−2^ at 600 and 500 °C, respectively. Furthermore, the polarization resistance of the LBC electrode at 600 °C was as low as 0.03 Ω cm^2^, significantly lower than that of state-of-the-art air electrode materials reported in the literature.

ML strategies have also been applied to the design and optimization of multi-element perovskites. Zhang *et al.* utilized an ML approach to design and optimize LnBaCo_2_O_5+*δ*_ (Ln = lanthanides) perovskites.^119^ The authors constructed a dataset comprising over 50 LnBaCo_2_O_5+*δ*_ (Ln = Pr, La, Ga, Nd, Ce, Sm) samples, systematically tuning their configuration entropy (*S*_config_) by doping the Ln site with 2 to 4 elements. The dataset was rigorously characterized across 20 performance metrics relevant to oxygen electrocatalysis. Contrary to the common assumption that increasing *S*_config_ alone enhances performance, a poor correlation was observed between *S*_config_ and electrochemical activity. Using Bayesian-optimized symbolic regression (BOSR) combined with a mixture of experts (MoE) ensemble model, multiple material “genes”, including the ionic radius, electronegativity, and *S*_config_, are linked to ORR catalytic activity.^120^ Over 177 000 potential LnBaCo_2_O_5+*δ*_ compositions were screened with high throughput, leading to the synthesis and validation of the three top candidate materials: (Pr_0.05_La_0.4_Nd_0.2_Sm_0.1_Y_0.25_)BaCo_2_O_5+*δ*_ (PLNSY), (Pr_0.25_La_0.35_Gd_0.05_Nd_0.05_Sm_0.05_Y_0.25_)BaCo_2_O_5+*δ*_ (PLGNSY), and (Pr_0.25_La_0.2_Gd_0.1_Nd_0.1_Ce_0.1_Sm_0.05_Y_0.2_)BaCo_2_O_5+*δ*_ (PLGNCSY). These materials exhibited higher oxygen vacancy concentrations, more disordered charge transport channels, and significantly lower polarization resistance than the control material PBC. In terms of electrochemical performance, a PCFC with a PLNSY electrode achieved an ASR of only 0.26 Ω cm^2^ at 550 °C and a PPD of approximately 1080 mW cm^−2^ at 600 °C. Moreover, PLNSY maintained an ASR of approximately 0.28 Ω cm^2^ after 150 h of operation in wet air at 600 °C, with a degradation rate of only 0.029% per 100 h, demonstrating excellent stability. Further analysis revealed that material durability is primarily governed by the first ionization energy, relative atomic mass, and ionic Lewis acid strength, rather than entropy alone.

In summary, the ML strategy integrates multiple descriptors to achieve rapid prediction of the catalytic activity and durability of electrode materials, providing a solid path for accelerating the development of commercially viable cathode materials.

#### 3.1.2 Design and applications of electrolyte materials

Lowering the operating temperature of CFCs is paramount for reducing system costs, broadening material selection, and enhancing long-term durability. However, this temperature reduction exponentially decreases the ionic conductivity of conventional oxygen-ion conducting electrolytes (*e.g.*, YSZ and SDC), which exhibit relatively high activation energies for ion transport, leading to prohibitive ohmic losses.^121^ Proton conducting electrolytes (PCEs) offer a compelling solution to this impasse. Due to the smaller size and lower migration barrier of protons compared to oxygen ions, PCEs typically exhibit lower activation energies for conduction. This fundamental characteristic creates a synergistic relationship: the reduced thermal energy at low operating temperatures (400–600 °C) is still sufficient to thermally activate proton transport in PCEs, enabling them to maintain high conductivity where oxygen-ion conductors fail. This “win–win” scenario, where low-temperature operation enables the practical use of PCEs and PCEs in turn enabling high-performance LT-CFCs, is a central driver of current research. Nevertheless, discovering PCEs that simultaneously achieve high proton conductivity, robust chemical stability (against CO_2_ and H_2_O), and good sinterability *via* traditional trial-and-error methods remains a formidable bottleneck. ML has introduced a paradigm shift in addressing this challenge, progressively evolving from a supplementary screening tool into a central driver of rational PCE design.^122–129^ Initially, ML served primarily in high-throughput virtual screening, enabling rapid mapping of vast compositional spaces to identify promising candidates and reducing reliance on purely empirical approaches.^128^ While effective for initial triage, these early strategies often offered limited atomic-scale insight into the fundamental ion transport mechanisms. Yamazaki's group provided a clear illustration of this progress. The team trained a gradient boosting regressor on a reliable dataset of 761 hydration-related data points, combining experimental results from 22 newly synthesized perovskites and 43 literature-reported compounds to construct data-driven structure–property maps that capture the physicochemical fundamentals of hydration. To enhance prediction accuracy for extrapolating to compositions beyond the well-studied BZCY perovskites, the researchers adopted knowledge-based target variable engineering where proton concentration was normalized by dopant content and expressed as log(*C*_h_/*C*_dopant_). This model enabled efficient screening of 8613 hypothetical perovskites, leading to the identification of SrSn_0.8_Sc_0.2_O_3−*δ*_ as an unconventional Sc-doped SrSnO_3_-based perovskite with no prior reports of proton incorporation or conduction. Experimental characterization verified the material's exceptional performance: it achieves a peak bulk proton conductivity of 1.4 × 10^−3^ S cm^−1^ at 380 °C, a value that surpasses most A^3+^B^3+^O_3_ perovskites, exhibits favorable hydration thermodynamics (Δ*H*_hyd_ = −76 ± 6 kJ mol^−1^), maintains a stable single-phase rhombohedral structure, and demonstrates proton-dominated conduction validated *via* isotope exchange experiments. While direct chemical stability tests (*e.g.*, against CO_2_ or H_2_S) were not reported, the elemental composition and inherent perovskite structure of the material suggest promising resistance to corrosive side reactions, which complements its conductivity window spanning 300–500 °C that aligns well with LT-CFC operational requirements. These combined attributes solidify its potential as a viable PCE for LT-CFCs. Extending this physics-informed ML framework beyond perovskites, the same group addressed the underexplored non-perovskite PCE landscape *via* an interpretable, defect-chemistry-trained approach. They constructed a dataset of over 3500 oxide configurations integrating structural descriptors (*e.g.*, cation coordination and bond valence sums) and critical defect chemistry parameters (dopant formation energy and oxygen vacancy concentration). These parameters are pivotal to proton conduction but were frequently overlooked in early ML models. A random forest (RF) model was trained on this dataset to co-predict the proton migration barrier and water incorporation capacity, two interdependent metrics that resolve the trade-off between proton mobility and concentration limiting non-perovskite development. The model exhibited remarkable effectiveness by identifying Pb-doped Bi_12_SiO_20_ (Bi_10.8_Pb_1.2_SiO_20−*δ*_) as a top candidate, which was experimentally validated in the first synthesis trial. This unprecedented sillenite-type PCE is composed solely of Group 14 (Si) and 15 (Bi and Pb) cations and delivers breakthrough performance. It features an ultra-low proton conduction activation energy of 0.42 eV, the lowest reported for non-perovskites and comparable to that of state-of-the-art perovskites. Proton-dominated transport is confirmed by a 1.2-fold conductivity reduction under D_2_O relative to H_2_O at 485 °C, and fast 3D proton migration along loosely bonded BiO_5_ polyhedra bypasses rigid SiO_4_ tetrahedra to underpin this performance. For LT-CFCs (300–600 °C), Pb-doped Bi_12_SiO_20_ holds substantial application potential. Its low activation energy translates to target conductivity in the range of ∼10^−3^ S cm^−1^. The absence of alkali earth and rare earth cations minimizes leaching in humid environments, a critical advantage over conventional perovskite electrolytes. Its mechanical strength of ∼80–120 MPa further mitigates cracking risks during fabrication and thermal cycling. Collectively, these studies demonstrate the ability of ML to unlock both perovskite and unconventional non-perovskite PCEs tailored to CFC requirements. A more profound advancement is now emerging through the deep integration of ML with computational physics, particularly *via* the development of accurate machine learning force fields (MLFFs).^129^ Trained on high-fidelity data derived from DFT, MLFFs overcome the long-standing compromise between quantum-mechanical accuracy and the spatiotemporal scale of classical molecular dynamics (MD) simulations. This convergence unlocks a new era of *in silico* microscopy, allowing researchers not only to predict but also to visually dissect and understand complex ion transport phenomena under realistic operating conditions. This transition from predictive screening to mechanistic simulation marks a cornerstone of next-generation PCE design.

This paradigm, which repositions ML as a precision instrument for simulation, is powerfully exemplified by the work of Yamazaki *et al.* in addressing the persistent challenge of proton trapping.^129^

**Fig. 3:**
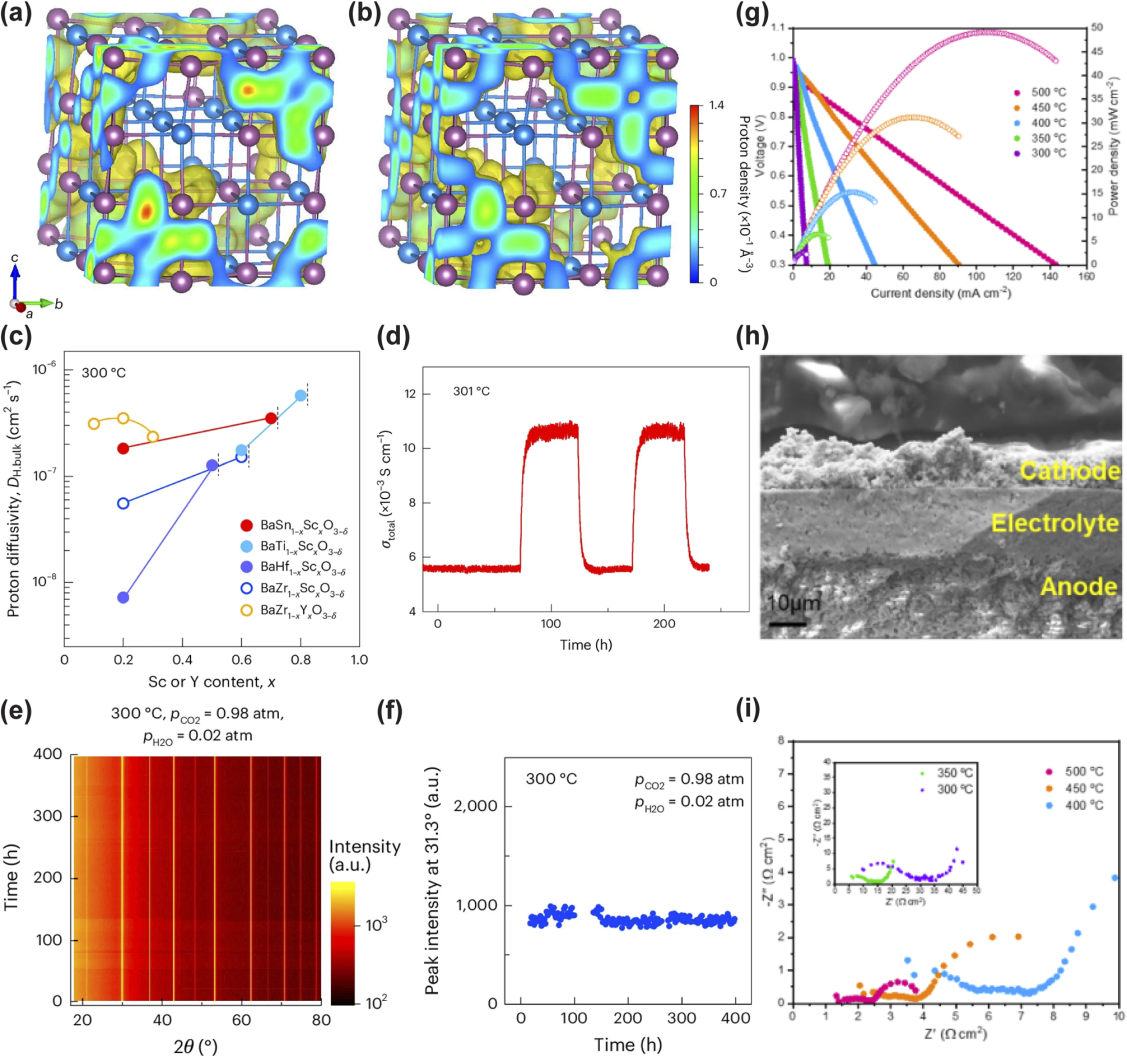
(a and b) Proton trajectories in 60 at% Sc-doped barium stannate at 227 °C (a) and 527 °C (b). (c) Bulk proton conductivity of Sc-doped barium stannates and barium titanates at 300 °C as a function of dopant content. (d) H–D isotope effect on total conductivity of BaSn_0.3_Sc_0.7_O_3−*δ*_ at 300 °C. (e and f) Chemical stability of hydrated BaSn_0.3_Sc_0.7_O_3−*δ*_ under concentrated humidified CO_2_: (e) time-dependent *in situ* X-ray diffraction patterns; (f) variation of secondary BaSc_2_O_4_ phase content with exposure time. (g–i) Electrochemical performance and microstructure of an anode-supported fuel cell with Sc-doped barium stannate electrolyte: (g) *I*–*V*–*P* curves at 300–500 °C; (h) cross-sectional SEM image near the electrolyte film; (i) electrochemical impedance spectra at open-circuit voltage. Reproduced with permission.^129^ Copyright 2025, Springer Nature.

Their study directly confronted the dual limitations of traditional methods: the prohibitive computational cost of direct *ab initio* MD for simulating long-range ion transport and the insufficient accuracy of classical force fields in modeling dopant–proton interactions. The key innovation was the construction of an MLFF using the Allegro architecture, trained on a carefully curated dataset of DFT calculations encompassing various atomic configurations of the Sc-doped perovskite system. This MLFF accurately captured complex interatomic interactions, particularly the critical proton-Sc associations, at a computational cost an order of magnitude lower than that of direct DFT. Deployed in large-scale MD simulations, it enabled the unprecedented tracking of proton trajectories over nanosecond timescales in a 3 × 3 × 3 supercell of BaSn_0.3_Sc_0.7_O_3−*δ*_ (BSS70). The simulations revealed the atomic-scale mechanism by which protons, while still associated with Sc dopants, migrate rapidly through a continuous ScO_6_ network without becoming deeply trapped, a dynamic process previously beyond the reach of standalone computational techniques (Fig. 3a and b).

Guided by these atomic-scale insights, the designed BSS70 electrolyte exhibited a high total proton conductivity exceeding 0.01 S cm^−1^ at 300 °C under a humidified atmosphere (pH_2_O = 0.02 atm) (Fig. 3c). Furthermore, reversible proton insertion and extraction was confirmed over 261 hours *via* H/D isotope exchange, showing a stable isotope effect of 1.95 (Fig. 3d). This performance was complemented by exceptional operational stability, demonstrated through its resilience over 398 hours under highly concentrated humidified CO_2_ (*p*CO_2_ = 0.98 atm) without carbonate formation and the maintained stability of Sn^4+^ under strongly reducing conditions (Fig. 3e and f). When the BSS70 electrolyte incorporating 5 at% NiO was integrated into an anode-supported fuel cell as an 18 µm-thin film, the resulting device exhibited an OCV of ∼1 V at 300 °C (Fig. 3g and h). The total cell resistance was 101.3 Ω cm^2^, with the electrolyte contributing only 5.1 Ω cm^2^, which corresponds to just 5% of the total resistance (Fig. 3i). This result underscores that cell performance was primarily limited by electrode kinetics rather than ionic transport. This body of work illustrates a complete ML-driven design cycle, wherein simulation-derived mechanistic understanding directly guides material synthesis, and subsequent experimental validation confirms the performance predictions.

In summary, the integration of ML into PCE research represents a fundamental shift in the materials development methodology. By bridging high-fidelity atomic-scale simulation with rational design principles, ML enables the simultaneous optimization of proton transport properties and operational stability that are essential for practical device implementation. This approach not only accelerates the discovery of high-performance PCEs but also provides crucial insights into the underlying mechanisms governing their behavior. As demonstrated through its application in addressing key challenges for LT-CFCs, ML has established itself as an indispensable tool for advancing next-generation electrochemical energy systems.

#### 3.1.3 Design and applications of anode materials

When a CFC operates on hydrocarbon fuels (*e.g.*, natural gas and biogas), the conventional nickel-yttria-stabilized zirconia (Ni-YSZ) anode is highly susceptible to carbon deposition.^130,131^ This carbon formation event results in the coverage of active TPB, pore occlusion, and may even trigger metal dusting corrosion and irreversible microstructural distortion, ultimately leading to cell performance degradation or even structural failure. Uncovering the evolution mechanism of the microstructure of CFC anode materials after long-term operation is useful for designing more durable anode materials. Through ML-assisted 3D microstructural analysis, researchers can analyse the three-dimensional microstructure evolution of the anode after long-term operation and reveal the carbon deposition mechanism of Ni-based ceramic anodes in methane.^132–134^

**Fig. 4:**
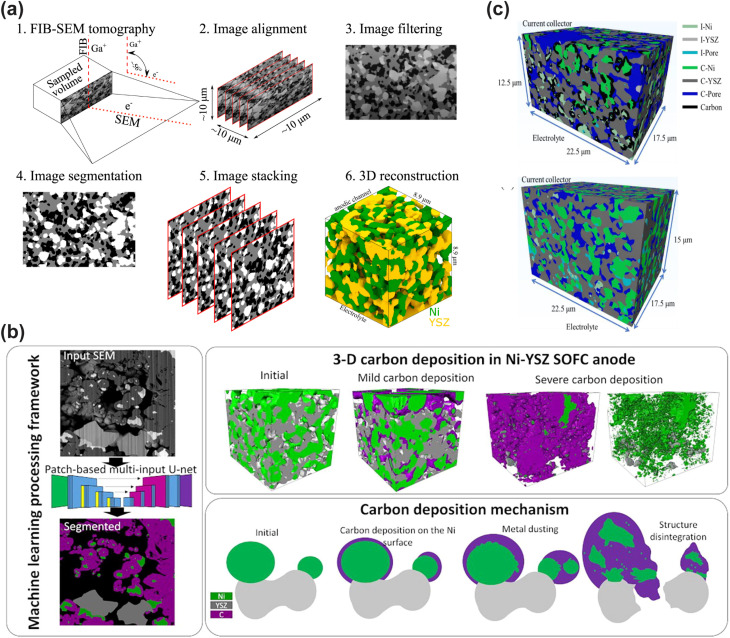
(a) Focused ion beam scanning electron microscopy application in SOFC research. Reproduced with permission.^132^ Copyright 2023, Elsevier. (b) The machine learning processing framework, 3D carbon deposition in the Ni-YSZ SOFC anode and carbon deposition mechanism. Reproduced with permission.^133^ Copyright 2023, Elsevier. (c) 3D-microstructures with carbon deposition. Ni30 after 1 h at open circuit and Ni30 after 12 h at *i* = 0.2 A cm^2^. ‘‘I” and ‘‘C” in the legend represent isolated and connected phases. Reproduced with permission.^134^ Copyright 2023, Elsevier.

The microstructure evolution mechanism of long-term operation of CFCs is a core challenge in designing durable energy systems. However, traditional research has mostly focused on the characterization of electrochemical performance, and a few studies involving microstructure analysis only rely on averaging parameters such as the phase volume fraction and curvature, making it difficult to capture the dynamic changes of three-dimensional microscopic characteristics of nickel (Ni), YSZ, and pore phases. Pawłowski *et al.* combined the 3D microstructure data obtained by focused ion beam scanning electron microscopy (FIB-SEM) with the persistent homology (PH) method in topological data analysis (TDA) to generate persistent diagrams (PDs) and persistent images (PIs) (Fig. 4a). Topologically invariant features of the structure at multiple scales, such as connected components, ring structures and cavities, can be extracted to more comprehensively describe the morphological changes during the material aging process. Persistent homology can automatically identify the “birth” and “death” processes of structural features without relying on manual annotation, revealing the persistence of features at different scales. This method is particularly suitable for analyzing multiphase and non-uniform composite materials, such as Ni-YSZ-pore three-phase structures.^132^

Traditionally, when using FIB-SEM for three-dimensional microstructure reconstruction, it is usually necessary to fill the sample with resin to enhance the image contrast. However, the low contrast between carbon and resin makes phase separation extremely difficult. In the samples without resin filling, the bottom surface of the open pores is visible in the cross-sectional image, further increasing the difficulty of segmentation. Sciazko *et al.* developed a machine learning image processing framework based on the U-net convolutional neural network to achieve precise and quantitative reconstruction of the three-dimensional structure of carbon deposits, and successfully realized multiphase segmentation and three-dimensional reconstruction of unfilled resin samples.^133^ Under mild conditions (steam-to-carbon ratio = 0.05, 0.5 hours), carbon formed thin layers on the Ni surface, leading to reversible performance degradation. Under severe conditions (steam-to-carbon ratio = 0, 3 hours), carbon deposition caused Ni powdering (metal dust corrosion), YSZ network deformation, and irreversible electrode damage (Fig. 4b).

Previous studies have mostly focused on carbon deposition behavior under open-circuit conditions. However, in actual operation, the anode is in a polarized state, with a continuous flow of oxygen ions, which alters the local reaction environment and affects the distribution and morphology of carbon deposition. However, due to the complex structure of Ni/YSZ anodes, it is difficult for traditional characterization methods to precisely distinguish the spatial distribution of carbon deposits and their local relationship with the TPB on a three-dimensional scale. Cui *et al.* reconstructed the 3D microstructure of Ni/YSZ after carbon deposition using FIB-SEM combined with machine learning segmentation techniques.^134^ They confirmed that carbon deposition in SOFC anodes under dry methane is not a uniform process but is finely regulated by local electrochemical environments. Polarization does not simply uniformly suppress carbon deposition but redistributes it, creating “clean zones” around active TPBs and forming “carbon-deposited zones” in adjacent regions (Fig. 4c). These findings highlight that tailoring the anode microstructure-particularly optimizing Ni particle size, TPB density, and ionic conduction phase connectivity is a viable strategy to leverage local electrochemical cleaning effects and mitigate overall degradation caused by carbon deposition, thereby enhancing the durability of SOFCs when operating on hydrocarbon fuels.

### 3.2 Density functional theory calculations

In addition to exploring the reaction mechanism in CFCs through experimental data, DFT calculations performed at the atomic level can also provide new insights into the reaction mechanism. A system composed of multiple atoms is essentially regarded as a system composed of electrons and nuclei in DFT calculations. By utilizing the theory of the interaction between nuclei and electrons, various physical and chemical properties of electrode materials can be predicted. Although it is unrealistic to accurately simulate the complex electrochemical processes on the surface of real oxides, DFT calculations can still provide valuable information. The combination of DFT calculations and experimental studies can generate new insights into the electrochemical processes in CFCs in a more comprehensive way.

#### 3.2.1 Design and applications of cathode materials

**Fig. 5:**
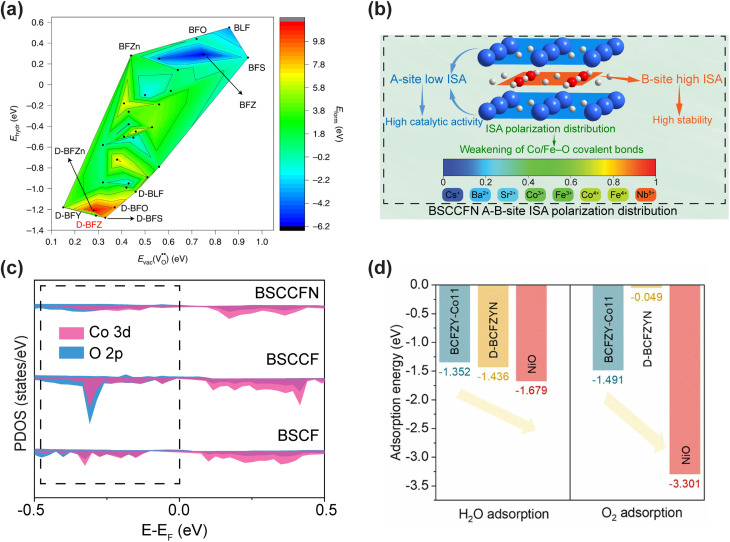
(a) Plot of computed substitutional defect formation energy, oxygen vacancy formation energy and hydration energy for BFO and its derivative materials. Reproduced with permission.^135^ Copyright 2022, Springer Nature. (b) Schematic illustration of BSCCFN A-B-site ionic Lewis acid strength (ISA) polarization distribution. (c) The PDOS of the O 2p and Co 3d orbitals for BSCF, BSCCF, and BSCCFN. Reproduced with permission.^136^ Copyright 2026, Elsevier. (d) H_2_O and O_2_ adsorption energies for the surface on the BCFZY-Co11, D-BCFZYN perovskites, and NiO. Reproduced with permission.^137^ Copyright 2023, Elsevier.

In recent years, the emergence of computational methods combining DFT and molecular orbital theory has provided a powerful tool for the rational design of CFC cathodes with excellent ORR activity and protonic conduction properties. Through a systematic investigation of A-site and B-site co-substitution in BaFeO_3_-based perovskites, Wang *et al.* discovered that introducing p-type defects, such as K, Na, Li, or A-site vacancies, effectively lowers the oxygen vacancy formation energy and hydration energy, thereby promoting oxygen ion and proton migration, while concurrently compromising the structural stability of the perovskite.^135^ To resolve this trade-off, B-site doping with elements like Zr, Y, and Sn was employed to enhance metal–oxygen bond strength and improve material stability (Fig. 5a). Guided by this theoretical framework, the optimal material Ba_0.875_Fe_0.875_Zr_0.125_O_3−*δ*_ (D-BFZ) was successfully synthesized. Experimental results demonstrated that a PCFC employing the D-BFZ cathode achieved high PPDs of 0.67 and 1.28 W cm^−2^ at 500 and 600 °C, respectively. Furthermore, D-BFZ exhibited excellent stability exceeding 200 h under high-temperature and high-humidity conditions, alongside a simple synthesis process that avoids non-commercial techniques such as complex nanostructuring or pulsed laser deposition.

Developing high-performance air electrodes that can be applied to both O–SOFCs and PCFCs at low temperatures is a major challenge in the field of solid-state electrochemistry. Conventional doping strategies often focus on enhancing either oxygen ion or proton conductivity individually, whereas simultaneously boosting triple-conducting (H^+^/O^2−^/e^−^) capability requires more sophisticated design principles. Our group recently adopted a similar A-site and B-site co-substitution strategy for cathode design, centered on creating a polarized distribution of ISA within the perovskite lattice, utilizing DFT calculations as a key guiding tool.^136^ Using BSCF as the parent material, the cubic perovskite cathode Ba_0.4_Sr_0.5_Cs_0.1_Co_0.7_Fe_0.2_Nb_0.1_O_3−*δ*_ (BSCCFN) was synthesized through A-site doping with low-ISA Cs^+^ and B-site doping with high-ISA Nb^5+^ (Fig. 5b). DFT calculations provided fundamental insights into the modification mechanism. Computation results revealed that the co-doping strategy elongates the Co/Fe–O bond lengths and reduces the hybridization between O 2p and Co/Fe 3d orbitals near the Fermi level, thereby weakening the covalency of the Co/Fe–O bonds (Fig. 5c). This electronic structure modulation significantly reduced the energy barriers for key processes: the oxygen vacancy formation energy of BSCCFN drastically decreased to −1.66 eV compared to 0.82 eV for pristine BSCF, while its hydration energy was reduced to −3.72 eV *versus* 9.2 eV for BSCF. Moreover, the calculated proton migration barrier for BSCCFN along a specific path (O1 to O10) was only 0.17 eV, substantially lower than the 4.79 eV for BSCF, indicating markedly enhanced proton mobility. Bader charge analysis further confirmed that electron redistribution primarily occurred around the B-site cations (Co/Fe), underscoring the role of ISA polarization in modulating B-site chemistry. These DFT-predicted performance improvements directly translated to exceptional low-temperature electrochemical performance in single cells. When employed as a cathode in a SOFC, the BSCCFN-based single cell achieved high PPDs of 1.12, 0.83, and 0.48 W cm^−2^ at 600, 550, and 500 °C, respectively. In a PCFC configuration, PPDs reached 0.84, 0.57, and 0.29 W cm^−2^ at the corresponding temperatures. These performance metrics significantly surpassed those of the unmodified BSCF electrode. The accelerated reaction kinetics were further corroborated by a reduced ASR in symmetric cells within this temperature range, attributable to the facilitated oxygen vacancy generation and enhanced dual oxygen-ion/proton conduction initially revealed by DFT.

Beyond single-phase cathodes, DFT is also frequently employed to guide the design of composite cathode materials. Liang *et al.* designed a nanocomposite air electrode, Ba_0.95_(Co_0.4_Fe_0.4_Zr_0.1_Y_0.1_)_0.95_Ni_0.05_O_3−*δ*_ (BCFZYN), comprising a major A-site deficient perovskite phase (D-BCFZYN) and exsolved NiO nanoparticles, and utilized DFT calculations to unveil the intrinsic synergistic mechanism behind its outstanding activity and durability.^137^ The study showed that the NiO (111) surface exhibits a very strong adsorption energy for O_2_ (−3.301 eV), significantly lower than that on the perovskite surface. This property makes NiO an excellent site for enhancing oxygen adsorption during the ORR (Fig. 5d). Concurrently, the major D-BCFZYN perovskite phase primarily facilitates oxygen ion and proton conduction. Compared to the control sample Ba_27_Co_11_Fe_10_Zr_3_Y_3_O_72_ (BCFZY-Co11, –1.447 eV), D-BCFZYN possesses a higher O-p band center (−1.327 eV), indicating superior potential ORR activity.^138^ Furthermore, the theoretical overpotential for the ORR on D-BCFZYN (0.565 eV) was lower than that on BCFZY-Co11 (1.283 eV). Therefore, a synergistic mechanism was proposed for the ORR process, wherein the NiO nanoparticles enhance O_2_ adsorption, and the major D-BCFZYN phase facilitates the conduction of protons and oxygen ions. The practical application of this electrode material in PCFCs was also evaluated. A single cell utilizing the BCFZYN cathode achieved a PPD of 936 mW cm^−2^ at 600 °C, with PPDs of 663, 427, and 252 mW cm^−2^ at 550, 500, and 450 °C, respectively. The cell also demonstrated operational stability for 400 h, highlighting the robustness of the nanocomposite structure. Zhao *et al.* developed an innovative “reverse atomic capture” strategy, introducing a tungsten source ((NH_4_)_10_W_12_O_41_·5H_2_O) to capture segregated Ba^2+^and Sr^2+^ ions on the surface of PBSCF, forming a heterostructured (Ba/Sr)(Co/Fe/W)O_3−*δ*_(BSCFW)@PBSCF composite.^139^ DFT was used to systematically compare the oxygen vacancy formation energy, hydration energy, and proton migration barrier between PBSCF and BSCFW. The results demonstrated that BSCFW exhibits a lower oxygen vacancy formation energy (0.23 eV), a more negative hydration energy (−1.83 eV), and a lower proton migration barrier (0.71 eV), significantly outperforming the pristine PBSCF material, thereby theoretically revealing the origin of its superior proton-involved ORR kinetics. Experimental validation showed that the 2 wt% W-modified 2W-PBSCF cathode achieved a PPD of 0.90 W cm^−2^ at 600 °C, maintaining 0.57 W cm^−2^ at 550 °C. The single cell operated stably for 240 h at 550 °C and 0.7 V without significant degradation, demonstrating exceptional durability.

DFT-driven material design and performance validation hold immense potential for developing high-performance, low-cost cathodes for low-temperature CFCs, providing useful guidance for the future rational optimization of CFC cathode materials.

#### 3.2.2 Design and applications of electrolyte materials

Achieving high ionic conductivity and robust stability at reduced temperatures is the central challenge for LT-CFC electrolytes. These macroscopic properties are governed by atomic-scale phenomena such as defect chemistry and ion migration, which are difficult to probe experimentally. DFT calculations have thus become indispensable, enabling quantitative prediction of key descriptors at the atomic level, including oxygen vacancy formation energy, hydration energetics, and proton migration barriers. By establishing fundamental structure–property relationships, DFT guides the rational design of novel electrolytes and transforms materials development from empirical trial-and-error into a theory-driven process.^140–147^ For instance, while BaZr_*x*_Ce_0.8−*x*_Y_0.1_Yb_0.1_O_3−*δ*_ ranks as a state-of-the-art PCE, it suffers from rapid degradation in CO_2_-containing environments. Liu's research group addressed this challenge through a series of systematic studies that employed DFT calculations to elucidate the underlying mechanisms and guide material optimization, including exploring Hf substitution, investigating the effects of typical lanthanide element doping and developing donor–acceptor synergistic regulation strategies, all aimed at resolving this critical stability-conductivity dilemma.^148–151^ Specifically, the proton conductivity and stability of BaHf_*x*_Ce_0.8−*x*_Y_0.1_Yb_0.1_O_3−*δ*_ (BHCYYb) were comprehensively investigated by Murphy *et al.*, who calculated proton concentrations and jump rates in BaHfO_3_ and BaZrO_3_ to resolve this matter.^151^ DFT calculations revealed that Hf substitution enhanced stability due to the higher Gibbs free energy for the reaction of BaHfO_3_ with CO_2_. At low Hf concentrations (≤30%), the material maintains high proton conductivity by optimizing defect chemistry to balance a higher proton concentration in BHCYYb against slower proton jump rates with the former compensating for the latter. The full cell incorporating the BHCYYb-3511 electrolyte (≈10 µm) demonstrated outstanding performance, achieving a PPD of 1.1 W cm^−2^ at 600 °C and high round-trip efficiencies of 78% for steam electrolysis at 1 A cm^−2^. More importantly, its superior stability was unequivocally confirmed by a mere 7.6% degradation after 700 hours of CO_2_–H_2_O co-electrolysis, a performance markedly superior to the 9.9% degradation observed for its BZCYYb-3511 counterpart, thereby providing strong experimental validation for the DFT predictions. Extending this methodology, Liu *et al.* performed comparative DFT assessments of BaHf_0.1_Ce_0.7_R_0.2_O_3−*δ*_ (BHCR172, where R = Yb, Er, Y, Gd, Sm) by calculating CO_2_/H_2_O adsorption energies and degradation reaction Gibbs free energy.^148^ DFT calculations showed that Yb doping enhances CO_2_/H_2_O tolerance. Owing to reduced surface adsorption and higher reaction Gibbs free energy, Yb-doped samples exhibit 3.28 eV CO_2_ adsorption energy compared to 3.51 eV in Y-doped materials. The BHCYb172-based cell (≈10 µm) demonstrated high performance with a PPD of 1.74 W cm^−2^ at 600 °C and an electrolysis current density of 2.0 A cm^−2^ at 1.3 V. It also exhibited exceptional stability, outperforming BHCY172 in a 30% CO_2_ atmosphere over 1000 hours of operation.

Building on this concept, Luo *et al.* implemented donor–acceptor synergistic regulation of oxygen vacancy content as the core modification strategy.^149^ DFT calculations uncovered the underlying mechanism, which showed that donor–acceptor co-doping modulates oxygen vacancy concentrations to reduce the adsorption energies of H_2_O and CO_2_. Specifically, for the BaNb_0.05_Ce_0.7_Yb_0.25_O_3−*δ*_, BaTa_0.05_Ce_0.7_Yb_0.25_O_3−*δ*_, and BZCYYb systems, the DFT-calculated Gibbs free energies (Δ*G*) for the chemical adsorption of H_2_O on the Nb-, Ta-, and Zr-doped surfaces at 500 °C were determined to be −0.112, −0.162, and −0.586 eV, respectively. This reduction in adsorption energy effectively inhibits surface contamination induced by the adsorption of H_2_O and CO_2_. The full cell incorporating the optimized BaNb_0.05_Ce_0.7_Yb_0.25_O_3−*δ*_ electrolyte (≈10 µm) demonstrated a PPD of 1.12 W cm^−2^ at 600 °C in fuel cell mode and a current density of 2.24 A cm^−2^ at 1.3 V in electrolysis mode. Moreover, long-term durability tests confirmed its outstanding stability, exhibiting negligible degradation in resistance over 500 hours under 30% H_2_O at 500 °C, markedly outperforming the continuously degrading BZCYYb electrolyte under identical conditions.

However, these conventional DFT studies typically focused on a limited set of candidate compositions guided by chemical intuition or prior experimental results, which inherently constrained the exploration speed and the potential for serendipitous discovery beyond established material systems. To overcome this limitation, high-throughput DFT screening has emerged as a transformative paradigm, shifting the research methodology from hypothesis-driven single-point analysis to data-driven comprehensive exploration.

**Fig. 6:**
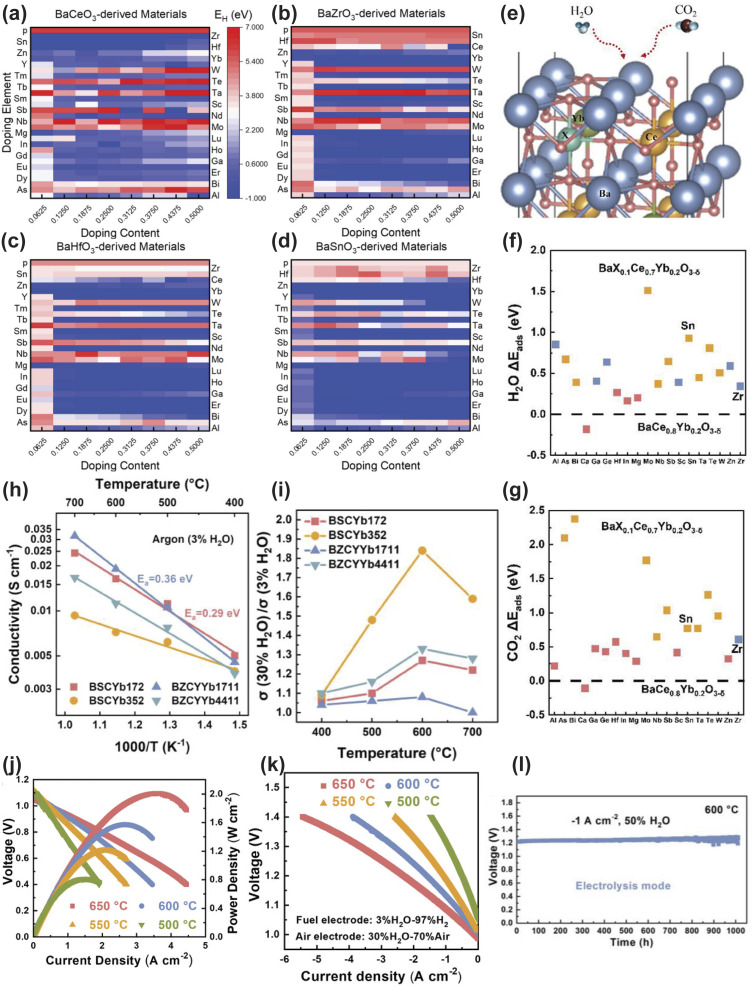
(a–d) Hydration energy (*E*_H_) of BaCeO_3_-, BaZrO_3_-, BaHfO_3_-, and BaSnO_3_-derived materials from high-throughput calculations. (e) Bulk model of BaX_0.1_Ce_0.7_Yb_0.2_O_3−*δ*_ (BXCYb172). (f) H_2_O adsorption energy (Δ*E*_ads_) of candidate electrolytes at 0 K. (g) CO_2_ adsorption energy (Δ*E*_ads_) of candidate electrolytes at 0 K. (h) Conductivity of BSCYb172, BSCYb352, BZCYYb, and BZCYYb4411 as a function of temperature. (i) Conductivity change of electrolytes when switching from 3% H_2_O to 30% H_2_O in Ar. (j) *I*–*V*–*P* curves of the Ni-BSCYb/BSCYb172/BPHYC single cell in fuel cell mode. (k) *I*–*V* curves of the single cell in electrolysis mode. (l) Long-term stability of the single cell in electrolysis mode at 600 °C. Reproduced with permission.^152^ Copyright 2024, Wiley-VCH GmbH.

The core distinction lies in its scale and automation. In 2024, Luo *et al.* constructed a computational model of a bulk perovskite structure with a supercell size of 
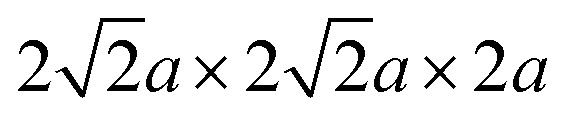
, containing 16 A-site and 16 B-site cations and systematically introduced 29 different dopant elements into BaCeO_3_, BaZrO_3_, BaHfO_3_ and BaSnO_3_ host lattices at a resolution of 6.25%.^152^ This approach enabled the screening of 932 unique perovskite compositions by calculating four key descriptors: oxygen vacancy formation energy (*E*_v_), hydration energy (*E*_H_) and H_2_O/CO_2_ adsorption energetics, as depicted in Fig. 6a–g. This automated, large-scale screening protocol, which would be impractically time-consuming *via* traditional DFT workflows, efficiently identified BaSn_*x*_Ce_0.8−*x*_Yb_0.2_O_3−*δ*_ (BSCYb) as a superior electrolyte system.

The optimal composition, BaSn_0.1_Ce_0.7_Yb_0.2_O_3−*δ*_ (BSCYb172), demonstrated a superior proton conductivity of 0.035 S cm^−1^ at 500 °C in wet argon (3% H_2_O), outperforming the benchmark BZCYYb (0.025 S cm^−1^) under identical conditions (Fig. 6h). Remarkably, its conductivity exhibited a strong positive dependence on steam pressure, increasing by 84% for BSCYb352 when the H_2_O concentration increased from 3% to 30% at 600 °C, highlighting its exceptional suitability for steam-rich electrolysis operations (Fig. 6i). In fuel cell mode, the cell with a BSCYb electrolyte (≈10 µm) achieved an outstanding PPD of 1.57 W cm^−2^ at 600 °C. In electrolysis mode, it delivered a high current density of 2.62 A cm^−2^ at 1.3 V and 600 °C, while also demonstrating exceptional durability for over 1000 hours under 50% H_2_O (Fig. 6j–l). This work not only exemplifies the transition from conventional DFT to high-throughput computational paradigms but also establishes a robust structure–property mapping methodology for the accelerated discovery of next-generation PCEs.

DFT unraveled atomic-scale mechanisms and guided material design, while experiments validated predictions and provided feedback for further optimization. This complementarity between them enabled tasks that conventional experimental techniques alone cannot achieve, such as quantifying adsorption energies, resolving defect dynamics and decoding dopant effects. This theoretical framework continues to empower the development of durable and high-performance electrolytes, paving the way for next-generation LT-CFCs with improved efficiency and operational longevity. Recently, Zou *et al.* conducted a comprehensive mechanistic study on mixed OH^−^/H^+^ conduction in SrZr_0.8_Y_0.2_O_3−*δ*_(SZYO20) and CaZr_0.8_Y_0.2_O_3−*δ*_(CZYO20).^153^ Their work addressed the critical challenge of clarifying the conduction mechanism in ceramic oxides at near-ambient temperatures (NATs), where conventional high-temperature PCEs exhibit poor conductivity and ambiguous charge carrier behavior. DFT calculations revealed that OH^−^ diffusion relies on oxygen vacancies and transient hydrogen bond formation, with migration energy barriers of 0.17–0.18 eV influenced by lattice free volume and defect association. Proton hopping and OH^−^ rotational diffusion were observed *via* AIMD simulations at 400 K, which confirmed that hydrogen bonding stabilized migration pathways. Neutron diffraction analysis combined with solid state nuclear magnetic resonance (NMR) measurements and fuel cell testing established that SZYO20 achieves a conductivity of 0.01 S cm^−1^ at 90 °C in a hydrated atmosphere, with a 70% OH^−^ transference number. This work deciphered the NAT ionic conduction mechanism, thereby enabling the design of durable ceramic electrolytes for LT-CFCs and thus overcoming the CO_2_-compatibility limitations. Based on the mixed OH^−^/H^+^ conductor SrZr_0.8_Y_0.2_O_3−*δ*_ (SZYO20), feasibility in both fuel cell and electrolysis applications was demonstrated. In fuel cell mode, an H_2_/air cell with a 1.8 mm-thick SZYO20 electrolyte achieved an OCV of 1.07 V at 20 °C, with a peak power density of 0.34 mW cm^−2^. When operated with ammonia fuel (35 wt% NH_3_ H_2_O + 3 M KOH), the direct ammonia fuel cell (DAFC) reached 30 mW cm^−2^ at 90 °C and showed stable operation for over 20 h, with the additional advantage of CO_2_ tolerance in air. In electrolysis mode, OH^−^ ion transport was directly confirmed *via* isotope experiments: at 2 V applied voltage, H_2_^18^O and D_2_O electrolysis using a dense CZYO20 pellet demonstrated effective migration of ^18^O and formation of HDO, proving the feasibility of low-temperature water splitting for hydrogen production.

#### 3.2.3 Design and applications of anode materials

Hydrogen is a commonly used fuel for fuel cells. Although it offers the advantage of zero carbon emissions, significant technical challenges in storage and transportation hinder its widespread commercialization. Ammonia, as a zero-carbon emission and hydrogen-rich carrier, has the characteristics of easy liquefaction for storage and convenient transportation. In recent years, it has become an alternative fuel that has attracted much attention in the field of fuel cells. Based on this, direct ammonia ceramic fuel cells (DA-CFCs) have become one of the research hotspots in current energy conversion technologies.^154,155^ However, the complete electro-oxidation of ammonia at the SOFC anode involves multiple proton–electron transfer steps and the formation of the N≡N triple bond, with a complex reaction pathway and significant kinetic barriers. Macroscopic electrochemical tests cannot clarify the microscopic reaction mechanism and rate-determining step. DFT calculations can construct surface reaction networks, revealing the reaction pathways at the atomic scale by calculating the adsorption energies and reaction energy barriers of each elementary step, and correlating the electronic structure of the catalyst (such as the d-band center) with its activity, providing a theoretical basis for the rational design of electrode materials.

Elmutasim *et al.* used DFT calculations to reveal the reaction mechanism of ammonia cracking and the hydrogen oxidation reaction on the Ni/YSZ (111) anode surface.^156^ By calculating the adsorption energy, transition state, and activation energy barriers of elementary reactions, DFT determined that nitrogen recombination and desorption are the rate controlling steps of NH_3_ decomposition, and pointed out the key role of the water formation step in H_2_ oxidation. These theoretical findings are directly correlated with electrochemical impedance spectroscopy (EIS) data, confirming that charge transfer resistance is the main source of polarization loss. The synergistic effect of DFT and experiments provides necessary mechanistic understanding for optimizing Ni/YSZ anodes.

**Fig. 7:**
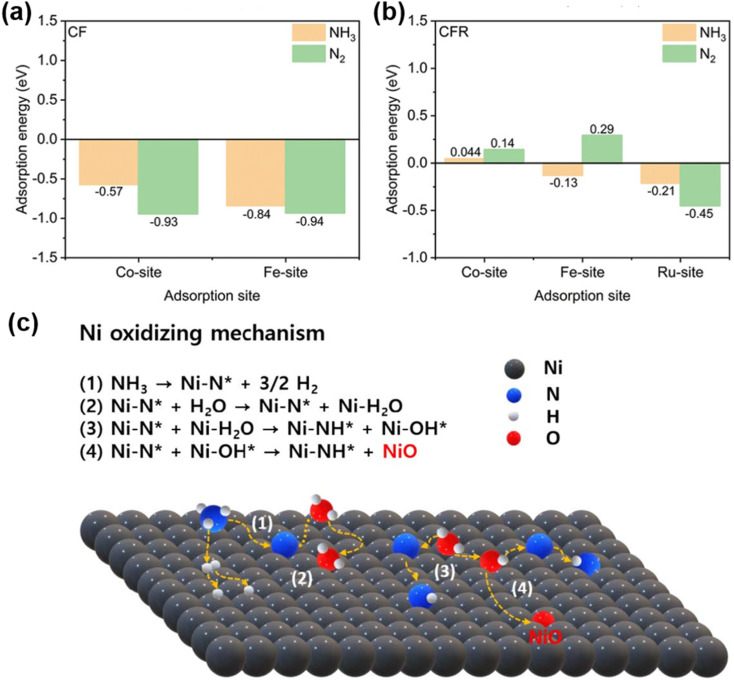
E(NH_3_) and E(N_2_) calculated for (a) CF (011) and (b) CFR (011). Reproduced with permission.^99^ Copyright 2024, Wiley-VCH GmbH. (c) Schematic diagram of the degradation of the Ni catalyst of a direct ammonia solid oxide fuel cell (DA-SOFC). Reproduced with permission.^157^ Copyright 2024, Elsevier.

Liang *et al.* investigated the role of Ru modification in CoFe alloy nanoparticles precipitated from the perovskite anode catalyst layer using DFT.^99^ By comparing the adsorption energies of NH_3_ and N_2_ on CoFe (011) and CoFeRu (011) surfaces (Fig. 7a and b), DFT revealed that Ru doping weakened nitrogen adsorption, thereby promoting N_2_ desorption. This theoretical insight explains the enhanced catalytic activity and durability observed in cells using modified anode layers. DFT thus guides the rational design of alloy catalysts by linking electronic structure modification with macroscopic performance improvement.

Lee *et al.* used DFT to reveal a previously unexplored degradation mechanism in Ni/YSZ anodes under wet ammonia conditions.^157^ Calculations show that the pre adsorbed nitrogen species (N*) generated by the decomposition of NH_3_ significantly reduce the energy barrier for H_2_O decomposition, leading to accelerated oxidation of nickel (Fig. 7c). This pathway was validated through X-ray photoelectron spectroscopy (XPS) and XRD analysis, which showed an increase in NiO formation under a wet NH_3_ atmosphere. The dynamic model based on DFT further quantified the rapid formation of oxidative species, providing an atomic level explanation for the degradation trend observed in the experiment.

### 3.3 High-entropy strategy

The investigation on high-entropy materials has expanded to ceramic systems. Five or more principal elements in near equimolar concentrations produce maximum configuration entropy, which were proposed in 2004 as innovative material designs with promising applications. A high-entropy ceramic is a solid solution of inorganic compounds with equal or near-equal atomic ratios of multiprincipal elements proposed in 2015, where the entropy stabilization was demonstrated in a mixture of oxides with more robust systems. Recently, new high-entropy materials have been developed as the electrolytes, cathodes and anodes of CFCs.

#### 3.3.1 Design and applications of cathode materials

Although traditional cobalt-based perovskite electrodes exhibit excellent mixed conductivity, they commonly suffer from severe surface segregation of Sr/Ba elements and phase separation under actual operating conditions of CFCs, leading to sluggish oxygen exchange kinetics and a sharp decline in durability.^158,159^ To address this challenge, the high-entropy strategy has been introduced into cathode material design. He *et al.* innovatively employed a wet-chemical solution infiltration method to construct a Ba/Sr-free perovskite coating with high B-site configurational entropy, PrNi_0.2_Mn_0.2_Co_0.2_Fe_0.2_Cu_0.2_O_3−*δ*_ (PNMCFC), on the surface of the classical PBC electrode backbone.^160^ This design ingeniously combines the stabilizing effect of high entropy with the physical barrier function of a conformal coating. The high-entropy character of PNMCFC confers superior thermodynamic stability, while this dense and continuous nanoscale coating achieves complete encapsulation of the PBC backbone, effectively isolating it from environmental moisture and thereby suppressing H_2_O-induced Ba segregation. Electrochemical performance was also significantly enhanced; the symmetric cell with the PNMCFC-PBC composite electrode exhibited an ASR as low as 0.72 Ω cm^2^ at 550 °C, substantially lower than that of the bare PBC electrode (1.16 Ω cm^2^). The corresponding single cell demonstrated outstanding power output, achieving PPDs of 0.64, 0.94, 1.30, and 1.72 W cm^−2^ at 450, 500, 550, and 600 °C, respectively. The microscopic mechanism behind this performance enhancement was elucidated through various characterization studies and theoretical calculations. XPS results confirmed that the PNMCFC coating effectively suppresses the high-temperature H_2_O-induced surface segregation of Ba from PBC, maintaining the stoichiometric stability of the electrode surface. Theoretical calculations reveal that the PNMCFC coating significantly increases the energy for Ba segregation from the PBC bulk to the surface from 0.48 eV to 2.36 eV, making Ba migration energetically highly unfavourable. Concurrently, the PNMCFC coating modulates the O 2p-band center at the interface, bringing it closer to the Fermi level, which enhances surface oxygen reaction kinetics.

Sun *et al.* successfully synthesized a triple-conducting perovskite oxide, BaCo_0.2_Fe_0.2_Zr_0.2_Sn_0.2_Pr_0.2_O_3−*δ*_ (BCFZSP), *via* a B-site high-entropy strategy.^161^ This material incorporates five transition metal cations in equimolar ratios at the B-site, forming a stable single-phase cubic perovskite structure that maintains structural stability even under atmospheres with *p*(H_2_O) as high as 50% atm, demonstrating the inherent excellent durability of high-entropy materials. The high-entropy design not only improved structural stability but also significantly enhanced the material's hydration capability and proton transport kinetics. Although the oxygen vacancy concentration of BCFZSP was lower than that of the classic triple-conducting reference material BCFZY, its proton concentration reached 6.92 mol% at 500 °C under 10% atm *p*(H_2_O), far exceeding the 1.34 mol% for BCFZY, indicating that the high-entropy composition greatly enhances the water adsorption capacity of oxygen vacancies. Electrical conductivity relaxation (ECR) analysis further revealed its exceptional ion transport properties: at 600 °C, when switching the gas environment from dry to humid, BCFZSP exhibited a non-monotonic relaxation behaviour characterized by an initial decrease followed by an increase in conductivity, qualitatively proving that its proton migration rate is faster than its oxygen ion migration rate, contrary to the behaviour of BCFZY. Quantitative calculations showed that the proton chemical diffusion coefficient (*D*_H,chem_ = 3.05–22.37 × 10^−6^ cm^2^ s^−1^) and water exchange coefficient (*k*_water_ = 2.01–21.29 × 10^−5^ cm s^−1^) of BCFZSP in the 500–700 °C range were significantly superior to those of both BCFZY and the BZCYYb electrolyte, establishing the kinetic foundation for its excellent electrochemical performance. Benefiting from this optimized triple conductivity, the BCFZSP electrode exhibited an ASR of 0.448 Ω cm^2^ at 550 °C. In single-cell tests, a cell employing the BCFZSP electrode achieved a PPD of 677 mW cm^−2^ at 600 °C. More importantly, this cell showed no significant performance degradation after 120 h of operation at 600 °C. Despite the effectiveness of B-site high-entropy engineering in enhancing the structural stability of perovskite materials, the electrocatalytic activity can be compromised in some high-entropy materials. This is because the B-site in perovskites typically hosts transition metal cations with high catalytic activity, and different doping elements can significantly influence the electrochemical properties of the perovskite. Neglecting the inherent electrochemical characteristics of dopant elements might lead to a misunderstanding of the relationship between configurational entropy and electrochemical performance.

**Fig. 8:**
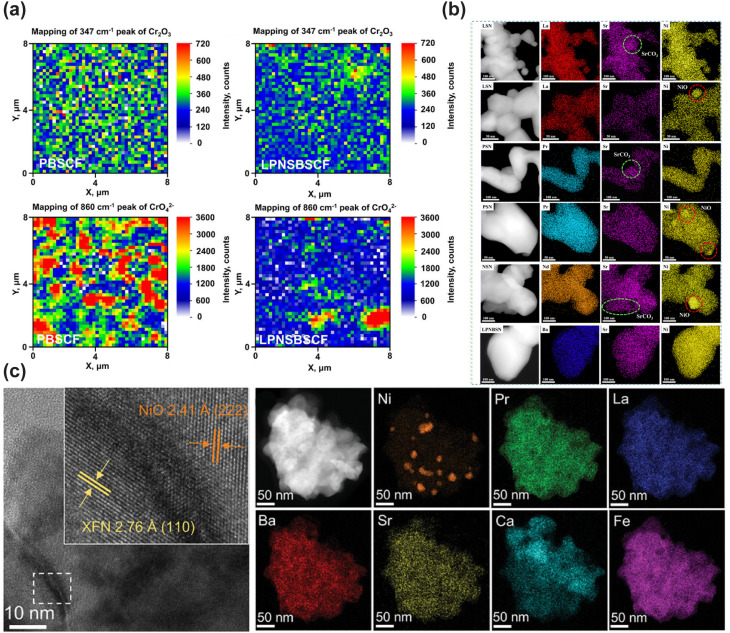
(a) Raman mapping of Cr_2_O_3_ (peak at 347 cm^−1^) and CrO_4_^2−^ (peak at 860 cm^−1^) on PBSCF and LPNSBSCF electrodes after Cr treatment. Reproduced with permission.^162^ Copyright 2025, Wiley-VCH GmbH. (b) Elemental mapping of LSN, PSN, NSN and LPNBSN after the thermal treatment. Reproduced with permission.^108^ Copyright 2025, The Royal Society of Chemistry. (c) HR-TEM image and EDX element mapping of N-XFN powder. Reproduced with permission.^167^ Copyright 2025, Wiley-VCH GmbH.

To address this issue, A-site HE oxides have been designed, showing great potential in enhancing the electrochemical performance and stability of oxygen electrodes for CFCs. Han *et al.* designed an A-site high-entropy layered perovskite material (La_0.25_Pr_0.25_Nd_0.25_Sm_0.25_)Ba_0.5_Sr_0.5_Co_1.5_Fe_0.5_O_5+*δ*_ (LPNSBSCF). The introduction of multiple rare-earth elements (La, Pr, Nd, and Sm) significantly increased the configurational entropy, effectively suppressing the surface segregation of alkaline earth metals Ba and Sr, thereby enhancing its Cr poisoning resistance and catalytic activity.^162^ Electrochemically, a single cell with LPNSBSCF achieved a PPD of 810 mW cm^−2^ at 600 °C, demonstrating its good application potential in low-temperature CFC systems. Theoretical calculations further revealed the advantages of the high-entropy structure. DFT indicated that LPNSBSCF exhibits lower oxygen vacancy formation energy and more negative oxygen adsorption energy, promoting ORR kinetics. Furthermore, the introduction of the high-entropy rare-earth layer significantly increased the segregation energy for Ba and Sr near the Ln–O layer surface, effectively inhibiting their surface enrichment at high temperatures and reducing the likelihood of forming detrimental phases like SrCrO_4_ and Cr_2_O_3_ with Cr vapour (Fig. 8a).^163,164^ He *et al.* designed and synthesized an A-site high-entropy perovskite material Pr_0.2_Ba_0.2_Sr_0.2_La_0.2_Ca_0.2_CoO_3−*δ*_ (HE-PBSLCC).^165^ This material incorporates five equimolar cations at the A-site, forming a stable single-phase cubic perovskite with the *Pm*3̄*m* space group. This A-site high-entropy design optimized the cathode material in several aspects. Firstly, it endowed the material with excellent structural stability, maintaining a pure phase structure without significant Sr/Ba segregation or secondary phase formation even after 100 h at 650 °C at a high steam concentration (20% H_2_O), whereas the low-entropy control material LE-PBSC exhibited severe segregation of the SrCoO_3−*δ*_ impurity phase. Secondly, the high-entropy effect effectively reduced the TEC to 23.8 × 10^−6^ K^−1^, superior to the 25.9 × 10^−6^ K^−1^ of LE-PBSC, resulting in better thermomechanical compatibility with the BZCYYb electrolyte. The HE-PBSLCC electrode also demonstrated exceptional ORR activity in practical application evaluations. A single cell utilizing it as the cathode achieved PPDs of 1.16 W cm^−2^ at 600 °C and 0.72 W cm^−2^ at 550 °C, respectively. Importantly, this high-entropy electrode demonstrated stable operation for over 270 h at 600 °C.

The A-site high-entropy strategy is not limited to perovskite structures but has been successfully extended to R–P oxides, offering a revolutionary solution to the long-standing activity–stability trade-off for such materials in CFCs. Although traditional R–P oxides like Ln_2_NiO_4+*δ*_ are noted for their excellent thermal stability and proton mobility, their electrochemical performance has consistently lagged behind that of top-tier perovskite electrodes. Yin *et al.* designed and synthesized an A-site high-entropy R–P oxide LPNBSN.^108^ By introducing five equimolar rare-earth and alkaline-earth metal cations at the Ln site, this material formed a unique high-entropy single phase with distinct advantages. Compared to traditional R–P oxides La_1.2_Sr_0.8_NiO_4+*x*_ (LSN), Pr_1.2_Sr_0.8_Ni_4+*x*_ (PSN), and Nd_1.2_Sr_0.8_NiO_4+*x*_ (NSN), the high-entropy design of LPNBSN brought multifaceted performance enhancements. Firstly, LPNBSN significantly reduced the interstitial oxygen formation energy to 0.27 eV, much lower than those of LSN (0.53 eV), PSN (0.74 eV), and NSN (0.46 eV), thereby greatly facilitating oxygen ion and proton transport. Secondly, LPNBSN lowered the energy barrier for *OOH formation during the ORR to 0.85 eV, significantly lower than the barriers for the comparison samples, and its O 2p-band center was closer to the Fermi level, indicating superior intrinsic ORR activity.^166^ More importantly, LPNBSN also exhibited a lower proton migration barrier, with the highest barrier being only 0.53 eV. Conductivity relaxation tests confirmed its faster bulk proton diffusion and surface exchange rates. These improvements in microscopic kinetics directly translated to exceptional macroscopic cell performance. A single cell with LPNBSN as the cathode achieved a remarkable PPD of 1872 mW cm^−2^ at 600 °C, significantly outperforming single cells based on traditional LSN, PSN, and NSN. Beyond exceptional activity, the high-entropy design also endowed LPNBSN with excellent structural stability. After annealing at 600 °C in air for 200 h, traditional LSN, PSN, and NSN all exhibited severe precipitation of SrCO_3_ and NiO impurity phases, whereas LPNBSN maintained its single-phase structure and elemental homogeneity (Fig. 8b). The segregation energy for Sr in LPNBSN was positive (11.85 meV), thermodynamically suppressing Sr segregation. Consequently, a single cell based on LPNBSN showed no significant degradation after continuous operation for over 500 h at 600 °C.

Notably, the high-entropy strategy also shows great potential in the design of multiphase composite air electrodes. Hu *et al.* reported an A-site high-entropy designed dual phase composite material, *x*NiO-Pr_0.2_La_0.2_Ba_0.2_Sr_0.2_Ca_0.2_Fe_0.8_Ni_0.2−*x*_O_3−*δ*_ (N-XFN), where high-entropy-induced strong lattice distortion promoted the exsolution of NiO nanoparticles from the perovskite B-site, forming a uniformly distributed dual phase structure that significantly increased the density of electrochemical active sites (Fig. 8c).^167^ Both the experiment and calculation confirmed that the A-site high-entropy design significantly reduced the separation energy of Ni, facilitating the spontaneous exsolution of NiO. This composite exhibited excellent electrochemical performance at 600 °C, achieving a PPD of 790 mW cm^−2^, which remained at 430 mW cm^−2^ even at 550 °C. Furthermore, the N-XFN electrode demonstrated exceptional long-term stability, operating steadily for 500 h and exhibiting excellent resistance to high-temperature and high-humidity environments.

The application of high-entropy engineering in the design and fabrication of air electrodes represents a highly promising strategy for developing moisture-tolerant, efficient, and long-lasting electrodes suitable for CFCs, promoting the advancement of CFCs towards lower operating temperatures and extended lifespan.

#### 3.3.2 Design and applications of electrolyte materials

**Fig. 9:**
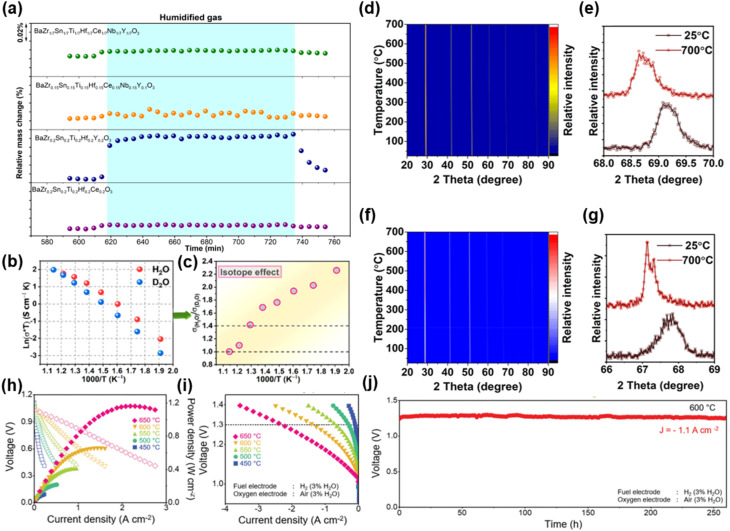
(a) Thermograms recorded at 300 °C upon isothermal switch between dry and humidified atmospheres. Reproduced with permission.^176^ Copyright 2020, American Chemical Society. (b and c) Hydration effects in H_2_O- *vs.* D_2_O-humidified wet air, derived from the ratios of total conductivity (*σ*_total_) in the two atmospheres. (d–g) Variable-temperature *in situ* XRD patterns and corresponding enlarged patterns. Reproduced with permission.^85^ Copyright 2022, American Chemical Society. (h) *I*–*V*–*P* curves measured at 450–650 °C. (i and j) Electrolysis (EC) mode performance of the single cell: (i) electrolysis performance when fed with humidified H_2_ (3% H_2_O) and humidified air (3% H_2_O) and (j) long-term durability test. Reproduced with permission.^181^ Copyright 2023, Wiley-VCH GmbH.

Low-temperature operation of CFCs demands electrolyte materials that exhibit both high ionic conductivity and long-term chemical stability, yet these two requirements are often mutually exclusive in conventional systems. Traditional proton conductors such as BaCeO_3_-based perovskites offer excellent conductivity but suffer from poor resistance against CO_2_ and H_2_O, while more stable materials like BaZrO_3_ require prohibitively high sintering temperatures.^168^ The high-entropy strategy has recently emerged as a transformative approach to overcome this performance-stability trade-off. By incorporating multiple principal cations into a single-phase lattice, high-entropy oxides leverage configurational entropy, severe lattice distortion, and sluggish diffusion kinetics to simultaneously enhance structural robustness and tailor defect chemistry for improved ion transport.^169–174^ In LT-CFCs, research on high-entropy electrolytes has progressed from initial explorations of entropy-driven stabilization toward more rational design paradigms. These designs combine multi-element doping with defect engineering to simultaneously optimize ionic transport and structural robustness, thereby improving low-temperature ionic conductivity while maintaining stability. This evolution marks a transition toward performance-oriented entropy engineering in advanced electrolyte development.^85,175–184^ High-entropy perovskite oxides (HEPOs) have garnered significant attention for proton conduction, with ongoing efforts to explore designs that enhance proton transport while preserving structural stability. Proton-conducting single-phase HEPOs were synthesized by Gazda *et al.*, with BaZr_0.2_Sn_0.2_Ti_0.2_Hf_0.2_Y_0.2_O_3−*δ*_ (BZSTHY) as the most representative example.^176^ In this material, Y-doping introduces oxygen vacancies, while the high configurational entropy endows it with high structural symmetry. Experimental results demonstrated that BZSTHY exhibited a proton conductivity of ∼10^−4^ S cm^−1^ at 400 °C in humid air with an activation energy of 0.53 eV, which was significantly lower than that of many classic low-entropy proton conductors. Furthermore, an isotope effect (*σ*_H_2_O_/*σ*_D_2_O_ = 1.8 at 400 °C) and humidity-induced conductivity enhancement further confirmed proton transport *via* the Grotthuss mechanism as the dominant conduction pathway (Fig. 9a). Although its conductivity still lagged behind that of state-of-the-art materials, the relatively low activation energy suggests considerable potential for this material. Further compositional optimization could lead to enhanced conductivity, warranting continued investigation. In 2022, our group conducted an essential milestone study by developing the HEPO BSZCYYbD in non-equimolar ratios that aimed to address key issues where traditional electrolytes lack stability and sinterability while existing HEPOs have low conductivity.^85^ Experimental results showed that this design significantly improved sinterability and grain size, synergistically regulated donor–acceptor oxygen vacancy content, and overcame limitations of prior HEOs such as high grain boundary resistance and defect clustering while achieving 94% densification. Inspiringly, BSZCYYbD exhibited the highest conductivity among HEPOs without sintering additives or transition metal oxides, reaching 8.3 mS cm^−1^ at 600 °C in humid air (Fig. 9b and c), which was linked to enhanced sintering properties and optimized oxygen vacancy concentration.

Theoretical calculations indicated that HEPO conductivity is independent of configurational entropy but correlates strongly with larger free volume and smaller tolerance factors. Notably, BSZCYYbD showed superior resistance to CO_2_/water corrosion, negligible electron conduction and a proton transport number above 0.93, which resolved the stability issues of traditional BaCeO_3_-based electrolytes (Fig. 9d–g). HEPO was first incorporated into a LT-CFC as an electrolyte (≈45 µm), achieving a competitive PPD of 318 mW cm^−2^ at 600 °C and demonstrating good operational stability with minimal voltage degradation over 100 hours, highlighting the potential of high-entropy design for robust PCEs.

Additionally, Oh and colleagues fabricated a series of novel HEPO electrolytes, including BaHf_1/6_Sn_1/6_Zr_1/6_Ce_1/6_Y_1/6_Yb_1/6_O_3−*δ*_ (BHSZCYYb). This cubic perovskite material exhibited improved chemical and structural stability compared to traditional PCEs, alongside enhanced conductivity relative to earlier high entropy materials (HEMs).^181^ In contrast to BSZCYYbD, the synthesis of BHSZCYYb incorporated 1 wt% NiO as a sintering aid. This approach yielded a record-high conductivity of 9.2 mS cm^−1^ at 600 °C in wet air, surpassing earlier HEPOs such as BZSTHY. Based on BHSZCYYb, an anode-supported LT-CFC was fabricated *via* an ultra-fast microwave-assisted sintering process, achieving a dense electrolyte layer with a thickness of approximately 5 µm. The cell demonstrated a PPD of 0.72 W cm^−2^ at 600 °C in fuel cell mode. In electrolysis cell mode, it exhibited a current density of 1.37 A cm^−2^ at 1.3 V and 600 °C, demonstrating stability over 250 hours (Fig. 9h–j). Through detailed analysis, we found that the superior performance of BHSZCYYb among these equimolar materials originated from its appropriate donor–acceptor element ratio, achieved by tailoring individual element concentrations to break the constraint of fixed stoichiometry and establish an optimal oxygen vacancy concentration. This carefully tuned vacancy profile enhanced proton transport while avoiding excessive vacancy clustering, a common issue in earlier HEPOs. These findings underscore that precise compositional design is essential for maximizing material performance, complementing the stability benefits derived from high-entropy effects. Furthermore, as mentioned above, sintering performance is equally critical to electrical transport properties and optimizing sintering processes indirectly through material design is a simple and effective approach. Recently, Xiang *et al.* presented a strategy for optimizing high-entropy electrolyte performance *via* A-site stoichiometry regulation.^183^ Combining synergistic defect engineering with entropy stabilization, the designed Ba_1.05_Ce_0.45_Zr_0.1_Y_0.1_Yb_0.1_Pr_0.10_Gd_0.15_O_3−*δ*_ (*S*_config_ = 1.565R) crystallized in a cubic perovskite structure. The approach integrated A-site stoichiometric compensation with 1.05 Ba excess to mitigate Ba loss during sintering and multi-cation doping of Pr^3+^ and Gd^3+^ to introduce oxygen vacancies. Incorporating 1 wt% NiO as a sintering aid further increased the densification to 95.53% and lowered the sintering temperature to 1400 °C. The material delivered a competitive proton conductivity of 8.95 mS cm^−1^ at 600 °C in wet air, while stability tests confirmed its resilience against CO_2_ and moisture. In fuel cell mode, the anode-supported cell employing a Ba_1.05_Ce_0.45_ZYYbPr_0.10_Gd_0.15_O_3−*δ*_ electrolyte (≈12 µm) achieved a PDD of 397 mW cm^−2^ at 600 °C. This work underscores how rational high-entropy design incorporating A-site compensation facilitates sintering optimization and advances HEPO performance.

The development of high-entropy electrolytes has progressed from initial definition-driven endeavors toward rational performance-oriented design. While non-equimolar compositions offer a broader design space than strictly equimolar systems, carefully engineered equimolar configurations have also achieved significant performance gains.^185–190^ Both strategies share a core objective that utilizes high configurational entropy to enhance structural stability while optimizing critical factors governing ionic transport including the crystal structure, sinterability and defect chemistry. Furthermore, HEMs intrinsically exhibit a cocktail effect, which expands design possibilities through multi-element incorporation. Future advances should prioritize this synergistic optimization that combines compositional flexibility of both equimolar and non-equimolar designs with targeted improvements in conductivity, stability and sinterability to propel next-generation electrolyte materials *via* purposeful entropy engineering.

#### 3.3.3 Design and applications of anode materials

In the development of CFC technologies, traditional nickel-based cermet anodes have long faced critical challenges under practical operating conditions, including susceptibility to catalytic degradation at high temperatures, limited resistance to carbon deposition, difficulties in balancing electrical conductivity with catalytic activity, insufficient chemical compatibility with common electrolyte materials, and inadequate long-term structural and performance stability. High-entropy alloys (HEAs), as a class of alloys formed by five or more equal or approximately equal amounts of metals, have tunable mechanical and catalytic properties.^191^ The high configurational entropy of HEAs endows them with excellent high-temperature structural stability; severe lattice distortion creates abundant active sites, directly optimizing the rate-limiting step of fuel gas adsorption/desorption, while slow diffusion effects significantly enhance resistance to coking and sintering. Meanwhile, the “cocktail effect” of multiple principal elements achieves functional complementarity, and the inherent high conductivity of the metallic-like material ensures efficient current transmission. Thus, the high-entropy strategy represents a comprehensive solution that simultaneously enhances anode catalytic activity, conductivity, and long-term stability through intrinsic material design.^192–194^

Given the aforementioned advantages of HEAs, numerous researchers have proposed their utilization as anode materials to enhance the long-term stability and performance of CFCs during the direct utilization of hydrocarbon fuels.^195–197^ The high configurational entropy effect facilitates the formation of homogeneous solid-solution structures comprising multiple metallic elements (*e.g.*, Ni, Co, Cu, Fe, and Mn). Through synergistic interactions among constituent elements, HEAs enable concurrent modulation of internal reforming reaction kinetics, enhancement of carbon deposition resistance, suppression of metallic sintering, and optimization of thermal distribution. This not only provides abundant active sites for improved catalytic performance but also exhibits excellent high-temperature oxidation resistance, corrosion tolerance, and structural stability. For example, Welander *et al.* developed a quinary HEA composed of Cu, Ni, Co, Fe, and Mn, which was combined with GDC to address two core challenges faced by traditional Ni-based anodes in direct internal reforming of methane for CFC applications: severe endothermic cooling effects and significant carbon deposition deactivation.^197^ Through its multi-principal-element synergistic effect, HEAs, on the one hand, provide moderate catalytic activity for methane steam reforming, avoiding the problem of local intense heat absorption and thermal stress caused by the overly rapid reaction of Ni-based catalysts; on the other hand, its unique alloy electronic structure significantly increases the activation energy for carbon formation, thereby endowing the anode with outstanding intrinsic anti-carbon deposition capability. In the fixed-bed reactor test at 600 °C, although the initial methane conversion rate of the HEA/GDC catalyst was lower than that of the highly active Ni/YSZ and Ni/GDC catalysts, its performance remained highly stable throughout a 30-hour isothermal test without any decline. In contrast, the two Ni-based catalysts experienced a continuous decrease in activity due to severe carbon deposition. Raman spectroscopy analysis of the post-test catalysts confirmed the presence of significant amorphous carbon and graphite carbon characteristic peaks on the surfaces of the Ni-based catalysts, while no carbon deposition signals were detected on the surface of the HEA/GDC. This fully demonstrates that at 600 °C, a temperature prone to carbon deposition, the HEA strategy sacrifices some extreme activity in exchange for crucial long-term catalytic stability and carbon tolerance, providing a promising alternative material for the direct use of hydrocarbon fuels in symmetric metal-supported SOFCs.

### 3.4 Defect engineering

Perfect crystals grow periodically in complete accordance with the Bravais lattice. However, in reality, people tend to artificially introduce point defects, line defects, and surface defects to control the electrical, thermal, magnetic and other properties of crystal materials, and thus control the catalytic and photoelectric conversion thermoelectric conversion and other properties of the materials. Cation defects and oxygen defects (sometimes called oxygen vacancies) not only play an important role in regulating the structure and ionic conductivity of fuel cell electrolyte materials, but are also crucial in improving the ionic conductivity and electrocatalytic activity of mixed ion-electron conductive electrode materials.

#### 3.4.1 Design and applications of cathode materials

**Fig. 10:**
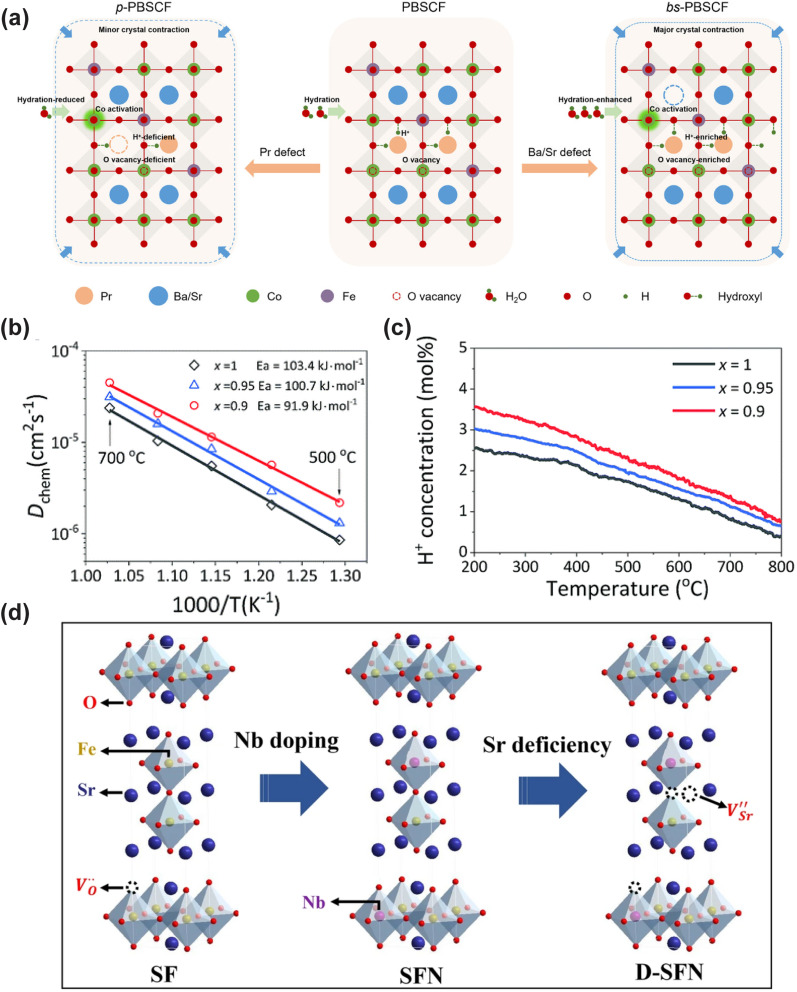
(a) Defect-mediated performance tuning in PBSCF perovskites. Reproduced with permission.^198^ Copyright 2025, Wiley-VCH GmbH. (b) Temperature-dependence of fitted *D*_chem_ values of A-site-deficient B_*x*_CFZY perovskites from 500 to 700 °C. (c) The calculated proton concentration of the B_*x*_CFZY at different temperatures. Reproduced with permission.^200^ Copyright 2019, The Royal Society of Chemistry. (d) Schematic diagram of Nb doping and Sr deficiency on S_3−*y*_FN_*x*_. Reproduced with permission.^40^ Copyright 2024, Springer Nature.

In recent years, defect engineering, particularly the selective manipulation of defects at the A-site cations of perovskites, has emerged as an effective strategy for enhancing the performance of cathode materials. Song *et al.* systematically investigated the effect of introducing different A-site cationic deficiencies in the A-site ordered layered perovskite PBSCF on its electrochemical properties, constructing Pr-deficient and Ba/Sr-deficient materials, denoted as p-PBSCF and bs-PBSCF, respectively.^198^ Their study revealed that Pr deficiencies activate Co sites by weakening the Co–O covalency, thereby enhancing oxygen catalytic activity. Concurrently, this reduction in oxygen vacancy concentration suppresses hydration-induced lattice expansion, strengthens the Ba–O/Sr–O bonds, inhibits Ba/Sr segregation, and consequently improves stability. However, the decreased oxygen vacancy concentration also limits the material's hydration capability and proton conductivity, thereby constraining the overall activity enhancement. In contrast, Ba/Sr deficiencies not only similarly activate Co sites but also significantly increase the oxygen vacancy concentration, facilitating the transport of oxygen ions and protons, which markedly enhances electrode activity (Fig. 10a). Furthermore, despite increased hydration, the larger-radius cationic deficiencies in bs-PBSCF result in a smaller unit cell compared to p-PBSCF, further strengthening the Ba–O/Sr–O bonds and suppressing Ba/Sr segregation, thus achieving superior stability. In performance evaluation, bs-PBSCF exhibited the most outstanding comprehensive performance. At 600 °C, a single cell based on bs-PBSCF demonstrated a PPD of 1.075 W cm^−2^, significantly higher than the 0.830 W cm^−2^ for p-PBSCF and 0.708 W cm^−2^ for pristine PBSCF. At 500 °C, the bs-PBSCF cell still delivered a PPD of 0.583 W cm^−2^, outperforming most reported advanced air electrode materials, such as BCFZY.^26^ Moreover, the single cell with bs-PBSCF showed no significant degradation over 900 h of operation, indicating excellent long-term operational stability.

Li *et al.* systematically studied the regulatory effects of A-site deficiencies on the structure, oxygen vacancy concentration, and hydration kinetics of Ba_1−*x*_Co_0.7_Fe_0.2_Zr_0.1_O_3−*δ*_ (B_1−*x*_CFZ, *x* = 0, 0.05, 0.1, and 0.15) cathode materials, finding that an appropriate Ba deficiency significantly enhances both ORR activity and proton conductivity.^199^ Their research demonstrated that with a 10% A-site deficiency (B_0.9_CFZ), the material maintains a pure perovskite phase structure, exhibits the highest catalytic activity, and shows good chemical compatibility with the BZCYYb electrolyte. The absence of A-site Ba^2+^ ions induces a significant increase in oxygen vacancy concentration *via* a charge compensation mechanism, thereby promoting the hydration reaction and enhancing the formation and migration of protonic defects. In single-cell application assessments, the cell employing the B_0.9_CFZ cathode achieved PPDs of 392 and 189 mW cm^−2^ at 600 and 500 °C, respectively, substantially higher than the 252 and 124 mW cm^−2^ for the pristine BCFZ cathode. This performance enhancement is primarily attributed to the A-site deficiency-induced increase in oxygen vacancy concentration, which enhances both bulk transport and surface exchange processes for oxygen ions and protons.

BCFZY has been confirmed as an excellent triple-conducting CFC cathode material. Modification engineering based on BCFZY has been widely reported. Ren *et al.* systematically investigated the influence of oxygen vacancy concentration on the triple-conducting properties of Ba_*x*_Co_0.4_Fe_0.4_Zr_0.1_Y_0.1_O_3−*δ*_ (BxCFZY, *x* = 1, 0.95, 0.9) by introducing A-site deficiencies.^200^ Their study indicated that A-site deficiencies primarily achieve charge compensation through the formation of oxygen vacancies rather than the oxidation of transition metal ions, thereby significantly enhancing oxygen ion and proton transport capabilities. Specifically, as the Ba content decreased (from *x* = 1 to *x* = 0.9), the oxygen vacancy concentration increased markedly, subsequently promoting both bulk diffusion and surface exchange of oxygen ions. For instance, the chemical bulk diffusion coefficient (*D*_chem_) and surface exchange coefficient (*K*_chem_) for B_0.9_CFZY reached 0.22 × 10^−5^ cm^2^ s^−1^ and 0.21 × 10^−4^ cm s^−1^ at 500 °C, respectively, significantly higher than those of the non-deficient BCFZY sample. Furthermore, the increase in oxygen vacancies also promoted the hydration reaction, increasing the proton concentration. Under conditions of 500 °C and 0.1 atm *p*(H_2_O), the proton concentration of B_0.9_CFZY reached 2.32 mol%, higher than the 1.74 mol% for the non-deficient BCFZY (Fig. 10b and c). These structural advantages directly translated into superior electrochemical performance. In symmetric cell tests, B_0.9_CFZY exhibited an ASR as low as 0.52 Ω cm^2^ at 500 °C in wet air, considerably lower than the 1.61 Ω cm^2^ for the non-deficient BCFZY. In single-cell tests, the cell utilizing the B_0.9_CFZY cathode achieved PPDs of 668.64, 548.07, and 376.27 mW cm^−2^ at 600, 550, and 500 °C, respectively, significantly outperforming the non-deficient BCFZY sample (481.84 mW cm^−2^ at 600 °C). Additionally, this cathode maintained stable output for over 120 h of continuous operation at 600 °C, demonstrating good durability.

Through a synergistic design combining A-site deficiency and B-site doping, Liang *et al.* systematically modulated the defect and Ni doping concentrations in BCFZY, thereby successfully fabricating a nanocomposite electrode material Ba_0.95_(Co_0.4_Fe_0.4_Zr_0.1_Y_0.1_)_0.9_Ni_0.1_O_3−*δ*_ (BCFZYN-095-01), which exhibits exceptional catalytic activity.^201^ After sintering, this material formed a composite structure consisting predominantly (97.6 wt%) of a perovskite phase with slight B-site deficiency, accompanied by surface-enriched NiO nanoparticles (2.4 wt%). The A-site deficiency promoted the exsolution of Ni, forming surface NiO nanoparticles that significantly enhanced oxygen surface exchange and steam adsorption capabilities. Concurrently, the B-site deficiency increased the oxygen vacancy concentration, improving the material's hydration capability and proton conductivity. This synergistic regulation of bulk and surface properties enabled BCFZYN-095-01 to exhibit very low ASR in symmetric cell tests and achieve an outstanding single-cell performance of 1100 mW cm^−2^ at 600 °C. Furthermore, a single cell based on this electrode demonstrated stable operation for 300 h at 550 °C without significant performance degradation, indicating exceptional durability. Similarly, employing a synergistic strategy of A-site deficiency and B-site doping, Yu *et al.* concurrently introduced Sr deficiency and Nb doping into the R–P perovskite Sr_3_Fe_2_O_7−*δ*_ (SF), designing D-SFN, which significantly enhanced electrocatalytic activity and durability (Fig. 10d).^40^ In this strategy, the incorporation of Nb^5+^ enhanced crystal structure stability, inhibiting the formation of the Sr_3_Fe_2_(OH)_12_ phase in wet air and thereby preventing structural degradation. Meanwhile, the introduction of A-site Sr deficiency effectively increased the oxygen vacancy concentration, promoting both bulk diffusion and surface exchange processes of oxygen ions. Electrochemical relaxation distribution analysis showed that the oxygen bulk diffusion coefficient and surface exchange coefficient of D-SFN were 1.9 times and 1.7 times higher than those of the non-deficient SF sample at 600 °C. Additionally, the introduction of Sr deficiencies enhanced the material's hydration capability, further promoting the kinetics of both the ORR and the water oxidation reaction. In symmetric cell tests, the D-SFN electrode exhibited an ASR of 1.209 Ω cm^2^ at 550 °C in wet air, significantly lower than that of the non-deficient SF and the solely Nb-doped SFN electrodes. In single-cell tests, the single cell employing D-SFN as the cathode achieved PPDs of 483 mW cm^−2^ and 361 mW cm^−2^ at 600 °C and 550 °C, respectively. Moreover, the single cell with the D-SFN cathode maintained stable operation for 142 h at 550 °C, demonstrating the good durability of D-SFN.

In summary, regulating oxygen vacancy concentration through cationic defect strategies can effectively enhance the triple-conducting properties of oxides, thereby significantly improving the ORR activity of CFC cathodes and the overall cell performance.

#### 3.4.2 Design and applications of electrolyte materials

**Fig. 11:**
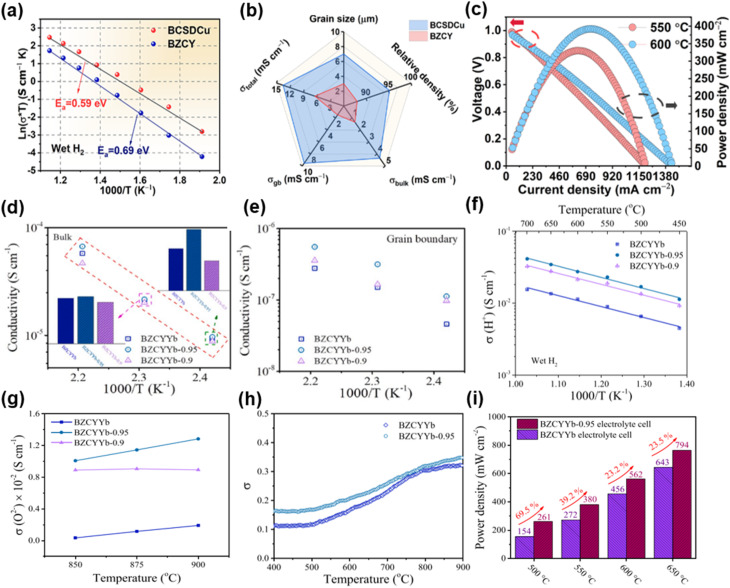
(a) Total conductivities of BZCY (1550 °C-sintered) and BCSDCu (1350 °C-sintered) in humid H_2_ at 250 °C. (b) Comprehensive comparisons of sinterability and electrical conductivities of the two electrolytes. (c) *I*–*V* and *I*–*P* curves of the BCSDCu-based single cell. Reproduced with permission.^206^ Copyright 2022, American Chemical Society. (d and e) Bulk and grain boundary conductivities of BZCYYb, BZCYYb-0.95 and BZCYYb-0.9 in H_2_. (f andg) Protonic and oxygen ionic conductivities of BZCYYb series electrolytes (humidified H_2_ ∼3% H_2_O and oxidizing atmosphere, respectively). (h) Oxygen non-stoichiometry of BZCYYb and BZCYYb-0.95. (i) PPD comparison of single cells with BZCYYb-0.95 and BZCYYb electrolytes. Reproduced with permission.^207^ Copyright 2020, Elsevier.

The ionic conductivity of electrolyte materials at reduced temperatures is fundamentally governed by the concentration and mobility of charge carriers, namely oxygen vacancies or protons. However, pristine oxide lattices often possess either insufficient or inappropriately distributed defect concentrations to support fast ion transport below 600 °C. Moreover, defect chemistry critically influences not only electrical performance but also sintering behavior, mechanical strength, and long-term operational stability. Defect engineering has therefore emerged as a direct and powerful strategy to address these challenges. By deliberately introducing or manipulating point defects, particularly through A-site or B-site cation nonstoichiometry, dopant incorporation, or oxygen vacancy tuning, researchers can precisely tailor the local coordination environment, optimize carrier concentration, and enhance transport kinetics without altering the parent crystal structure.^202–204^ For example, studies by our group demonstrated that an appropriate concentration of oxygen vacancies is critical to the overall performance of PCEs.^205^ Moreover, through Sn–Dy–Cu triple doping into BZCY (BCSDCu) to ensure a suitable oxygen vacancy concentration and carefully balancing the ionic radii and electronegativities of constituent elements, multiple improvements in proton electrolyte properties were achieved, including enhanced electrical transport performance, material stability and sintering performance, where the densification sintering temperature of BCSDCu was reduced by 200 °C compared with the parent material, the conductivity increased by two times and the cell with BCSDCu electrolyte (≈40 µm) delivered a PPD of 390 mW cm^−2^ at 600 °C (Fig. 11a–c).^206^

In a separate study, He *et al.* employed a defect engineering strategy by introducing 5 mol% B-site cation deficiency Ba(Zr_0.1_Ce_0.7_Y_0.1_Yb_0.1_)_0.95_O_3−*δ*_ (BZCYYb-0.95) and using sol–gel synthesis to generate oxygen vacancies, thereby optimizing grain boundary conductivity. This approach mitigated lattice distortion through balanced defect chemistry, which promoted grain growth from 0.38 µm in stoichiometric BZCYYb to 1.18 µm for BZCYYb-0.95. It also reduced grain boundary resistance (Fig. 11d and e) and increased oxygen vacancy concentration, facilitating dual-ion conduction. Mechanistically, this strategy increased protonic conductivity to 4.6 × 10^−2^ S cm^−1^, which was 2.5 times higher than before (Fig. 11f) and increased oxygen ionic conductivity by 6.3 times (Fig. 11g). The data in Fig. 11h reveal a consistently higher population of oxygen vacancies in BZCYYb-0.95 compared to pristine BZCYYb from 400 to 900 °C. One plausible explanation for the superior ionic conductivity of BZCYYb-0.95 is this significant difference in their oxygen defect chemistry. An anode-supported cell incorporating a BZCYYb-0.95 electrolyte (≈12 µm) achieved a PPD of 794 mW cm^−2^ at 650 °C. Moreover, it showed no significant degradation during a 300-hour durability test conducted at 550 °C (Fig. 11i). In conclusion, defect concentration significantly influences multiple material properties of the materials. Only an appropriate concentration of defects can improve their overall performance, thereby further promoting the development of LT-CFCs.^207–209^

#### 3.4.3 Design and applications of anode materials

To enhance the catalytic activity, structural stability, and anti-coking/nitriding capability of anode materials for CFCs, many researchers have introduced A-site defects in perovskite anode materials to generate additional oxygen vacancies. This approach not only reduces the reduction energy barrier of B-site metal ions (*e.g.*, Fe, Co, and Ni), promoting their *in situ* exsolution under reducing atmospheres to form highly dispersed and firmly anchored nanoparticles (*e.g.*, Co, Ni, FeNi, *etc.*), but also strengthens the valence compensation capacity and structural integrity of the perovskite framework during the reduction process.^210–212^ This strategy effectively improves the electrode's conductivity and triple-phase interface reaction activity. By anchoring the exsolved nanoparticles (NPs), it inhibits their high-temperature agglomeration and migration, significantly enhancing the cell's power output, fuel conversion efficiency, and long-term operational stability. Additionally, A-site defect regulation further optimizes the oxygen vacancy concentration and surface adsorption characteristics of the materials, enhancing their catalytic decomposition and electrooxidation kinetics for fuels such as ethane and ammonia, demonstrating universality and application potential across various fuel systems.^213^

**Fig. 12:**
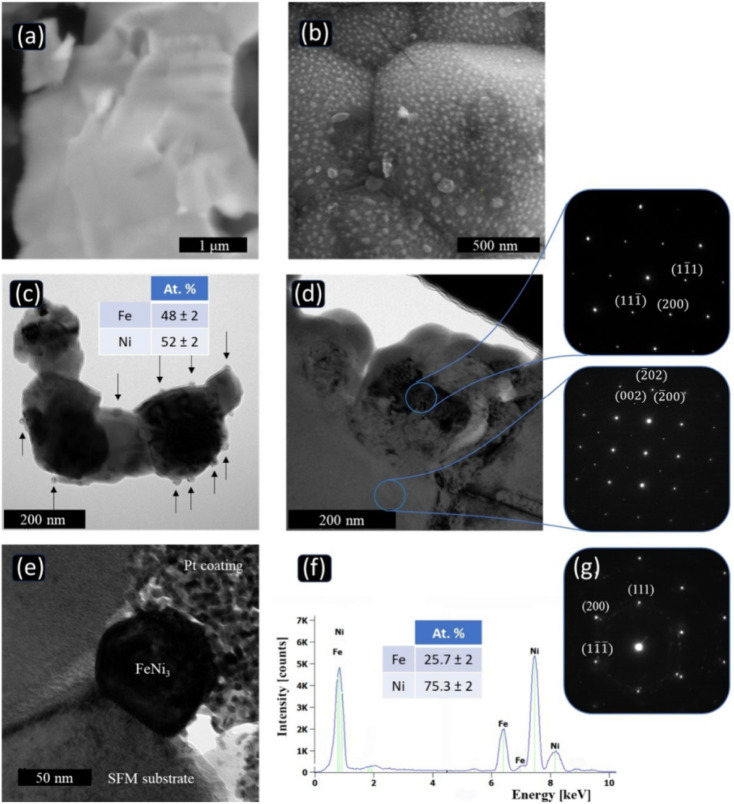
SEM images of the (a) air-sintered SFNM pellet. (b and c) SFNM pellet and powder after reduction at 800 °C under humidified H_2_ (3% H_2_O) and the corresponding TEM cross-sectional image. (d) TEM cross-sectional image with SAED patterns taken from the substrate and the exsolved NPs in the [011] and [010] zone axes, respectively. (e) Cross-sectional TEM image of the sample after exposure to NH_3_. (f) EDS spectrum and (g) SAED pattern taken from the NP shown in (e) in the [011] orientation. Additional spots originate from Pt coating added on top of the NP as part of the FIB sample preparation. Reproduced with permission.^214^ Copyright 2024, American Chemical Society.

To develop a high-performance and stable anode material suitable for symmetric DA-SOFCs, Rahumi *et al.* synthesized a novel Ni-doped double perovskite material, Sr_1.9_Fe_1.4_Ni_0.1_Mo_0.5_O_6−*δ*_ (SFNM).^214^ By *in situ* precipitating FeNi_3_ nanoparticles under anode conditions, the material enhanced electrocatalytic activity for ammonia decomposition and hydrogen oxidation reactions (Fig. 12). In terms of catalytic activity, although the ammonia conversion rate of SFNM at 600 °C is lower than that at elevated temperatures (*e.g.*, 89.5% at 700 °C), it still significantly surpasses that of the undoped SFM reference group. This enhancement is attributed to the *in situ* exsolved FeNi_3_ nanoparticles on the surface, which provide highly active sites for ammonia decomposition. Regarding long-term stability, the cell exhibits outstanding durability in high-temperature ranges (*e.g.*, 700–800 °C), with a degradation rate as low as 0.48% per 100 h. However, at 600 °C, the diminished ammonia decomposition rate leads to insufficient local hydrogen partial pressure, which may compromise stability. Impedance analysis further reveals that at 600 °C, the mass transport process constitutes the dominant contributor to the polarization resistance, accounting for 88–96.2%, indicating that ammonia diffusion and dissociative adsorption have become the rate-determining steps. Despite the performance attenuation at lower temperatures, the SFNM-based electrode maintains robust structural stability and catalytic activity, demonstrating its promising potential for application in intermediate- and low-temperature DA-SOFCs.

### 3.5 Mechanical mixing

Mechanical mixing, a pivotal aspect of the composite strategy, plays a vital role in the development of LT-CFCs. It provides distinct advantages in the design and application of materials for each cell component. Composites, which consist of two or more substances with different physical and chemical characteristics, demonstrate enhanced performance beyond the sum of their individual parts. In LT-CFCs, mechanical mixing enables the formation of composites that effectively utilize the synergistic effects among materials. For example, combining an ion-conducting electrolyte with an electrode material can significantly expand the three-phase reaction interface. This not only reduces interfacial resistance but also substantially improves the overall performance of LT-CFCs. Notably, this approach has been widely and successfully applied in manufacturing CFC materials, resulting in remarkable enhancements in their low-temperature performances.

#### 3.5.1 Design and applications of cathode materials

In CFCs, the sluggish kinetics of the ORR at the cathode is one of the key factors limiting their performance. To enhance the ORR activity and durability of CFC cathodes, the mechanical mixing strategy is widely adopted, which involves constructing composite cathodes by physically mixing the cathode material with a second-phase material possessing complementary properties. Li *et al.* systematically investigated the performance of a composite cathode formed by mechanically mixing slightly Mo-doped SrFe_0.93_Mo_0.07_O_3−*δ*_ (SFM0.07) with GDC (denoted as SFM0.07 + GDC) in CFCs.^215^ Their study revealed that the SFM0.07 cathode exhibited a PPD of 0.24 W cm^−2^ at 600 °C. In contrast, the PPD of the SFM0.07 + GDC cathode after mechanical mixing with GDC was significantly enhanced to 0.35 W cm^−2^ at the same temperature. This improvement was attributed to the extended TPBs formed within the composite cathode, which facilitated the processes of oxygen surface adsorption, dissociation, and bulk diffusion, thereby substantially reducing the cathode's polarization resistance. The SFM0.07+GDC composite cathode also demonstrated excellent stability during long-term operation and thermal cycling tests. A single cell based on this composite cathode showed no significant performance degradation after 270 h of continuous operation. More importantly, the PPD of the single cell remained stable after 20 rapid thermal cycles. These results fully demonstrate the effectiveness of the mechanical mixing strategy in enhancing cathode ORR activity, thermomechanical compatibility, and long-term operational stability.

**Fig. 13:**
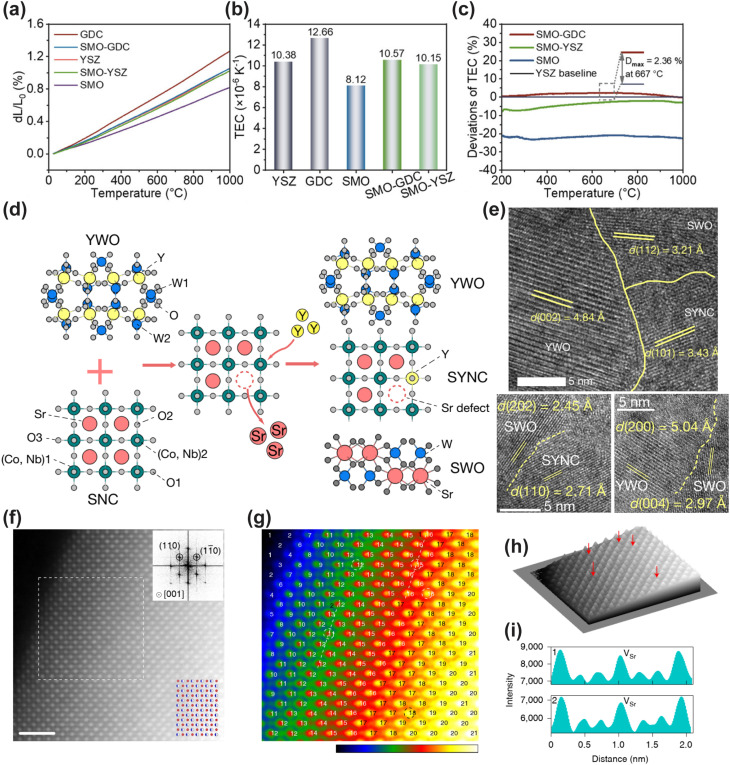
(a) Thermal expansion curves and (b) TECs of Mn-based mullites, GDC, YSZ, and their composites from RT to 1000 °C. (c) The TEC deviation between SMO and its composites relative to YSZ. Reproduced with permission.^216^ Copyright 2025, Wiley-VCH GmbH. (d) Schematic illustration of the formation mechanism of c-SYNC. (e) HRTEM images of interfaces of the YWO, SWO and SYNC phases. Reproduced with permission.^218^ Copyright 2021, Springer Nature. (f) HAADF-STEM image taken in the [001] viewing direction of Sr_vac_/LSCF095, enlarged view of the region in (f) marked with a dashed box (g), and the corresponding three-dimensional intensity surface plot (h). (i) Intensity line profiles extracted along the dashed lines (1 and 2) in (g). Reproduced with permission.^221^ Copyright 2022, Springer Nature.

Another application of the mechanical mixing strategy lies in reducing the TEC of composite cathodes, enabling good thermomechanical matching with the electrolyte while maintaining high catalytic activity. Duan *et al.* fabricated an SMO-GDC composite by mixing the Mn-based mullite material SmMn_2_O_5_ (SMO) with GDC.^216^ SMO itself exhibits an extremely low TEC (8.12 × 10^−6^ K^−1^), which is significantly lower than that of the conventional electrolyte YSZ (10.38 × 10^−6^ K^−1^). By mechanically mixing GDC (TEC = 12.66 × 10^−6^ K^−1^), the resulting SMO-GDC composite exhibited a TEC deviation of only 2.36% from that of YSZ over a wide temperature range, markedly superior to traditional cathode materials (Fig. 13a–c). The cell with the composite cathode achieved a PPD of 107.1 mW cm^−2^ at 600 °C. Furthermore, the single cell based on this cathode showed no degradation during a 300-h constant current discharge at 0.6 A cm^−2^, demonstrating exceptional long-term stability. Mechanistic studies indicated that the introduction of GDC not only provided ionic conduction pathways but also promoted interfacial charge transfer through the formation of heterojunctions with SMO. Liu *et al.* prepared PBSCF-*x*SZM composite cathodes with varying SZM contents (*x* = 0–30 wt%) by mechanically mixing the NTE material Sm_0.85_Zn_0.15_MnO_3_ (SZM) with the perovskite PBSCF.^217^ Electrochemical performance tests identified PBSCF-20SZM as the optimal composition, achieving a PPD of 0.534 W cm^−2^ at 600 °C, which represents an improvement of over 50% compared to the pure PBSCF cathode. The ASR of this composite cathode at 600 °C was as low as 0.15 Ω cm^2^, only 36.5% of that of pure PBSCF, indicating significantly enhanced ORR kinetics. Through various characterization techniques, including high-resolution transmission electron microscopy (HR-TEM), high-temperature X-ray diffraction (HT-XRD), distribution of relaxation times (DRT) analysis, electron paramagnetic resonance (EPR), oxygen temperature-programmed desorption (O_2_-TPD), and DFT calculations, it was found that the performance enhancement primarily originated from the compressive lattice strain introduced by SZM during high-temperature sintering. This strain reduced the oxygen vacancy formation energy and promoted oxygen adsorption, dissociation, and ion exchange processes. Moreover, the PBSCF-20SZM composite cathode remained stable after 800 h of continuous operation at 600 °C, with negligible performance degradation, attributed to its more compatible thermal expansion behaviour with the electrolyte. Zhang *et al.* proposed a mechanical mixing strategy based on NTE materials, successfully achieving effective matching of the thermal expansion behaviour between the cathode and electrolyte.^218^ Their study selected the cobalt-based perovskite SrNb_0.1_Co_0.9_O_3−*δ*_ (SNC), which exhibits high electrochemical activity but a large TEC (19–24 × 10^−6^ K^−1^), as the positive thermal expansion phase, and compounded it with the NTE material Y_2_W_3_O_12_ (YWO, TEC ≈ −7 × 10^−6^ K^−1^). After reactive sintering at 800 °C, an interfacial reaction occurred, forming a SrWO_4_ (SWO) interfacial phase and introducing A-site defects into SNC, resulting in the formation of a Sr_*x*_(Y_*y*_(Nb_0.1_Co_0.9_)_1−*y*_)O_3−*δ*_ (SYNC) phase with optimized oxygen vacancy concentration, ultimately yielding the composite electrode c-SYNC (Fig. 13d and e). The average TEC value of this composite electrode in the range of 100–800 °C was 12.9 × 10^−6^ K^−1^, highly compatible with the commonly used electrolyte SDC (12.3 × 10^−6^ K^−1^) and much lower than that of pure SNC (20.5 × 10^−6^ K^−1^). Electrochemically, the ASR of c-SYNC was only 0.063 Ω cm^2^ at 600 °C. In single-cell tests, a cell employing a 40 µm thick c-SYNC cathode achieved a PPD of approximately 525 mW cm^−2^ at 600 °C. Furthermore, the c-SYNC electrode exhibited excellent stability in thermal cycling tests, with the ASR increasing by only 8% after 40 cycles, compared to a 19% increase for the SNC electrode. Structural characterization revealed no significant cracks or delamination in the c-SYNC electrode, indicating good thermomechanical compatibility. Although mechanically mixing NTE materials with perovskite-based electrodes can mitigate thermal expansion mismatch between the electrode and electrolyte in CFCs, conventional NTE composites often introduce significant interparticle thermal stress, leading to microcracks and performance degradation. To address this issue, Zhang *et al.* proposed an innovative interfacial oxide “wedging” method *via* high-temperature reactive calcination.^219^ By mechanically mixing HfW_2_O_8_ as the NTE component with BSCF perovskite and calcining at 1100 °C, secondary phases such as Co_3_O_4_, Fe_3_O_4_, BaHfO_3_, and Sr_3_WO_6_ were *in situ* generated at the interfaces. These phases effectively “wedged” between the HfW_2_O_8_ and perovskite particles, acting as transitional buffer layers to alleviate thermal stress and enhance interfacial bonding. The TEC of the optimized composite (20 wt% HWO, denoted as NTE-BSCF) was significantly reduced from 22.4 × 10^−6^ K^−1^ for pure BSCF to 14.6 × 10^−6^ K^−1^, highly compatible with the TEC of the SDC electrolyte (12.3 × 10^−6^ K^−1^). Additionally, the mechanical properties of the composite were markedly improved: the elastic modulus increased by 102% and the hardness by 138%, attributed to the Hall–Petch strengthening effect induced by the interfacial oxides.^220^ Electrochemically, the ASR of NTE-BSCF was only 0.028 Ω cm^2^ at 600 °C, half that of pure BSCF (0.065 Ω cm^2^). In single-cell tests, an Ni-YSZ supported cell with an NTE-BSCF electrode achieved a PPD of approximately 600 mW cm^−2^ at 600 °C, while the corresponding large-area BZCYYb-based cell reached about 400 mW cm^−2^ at 600 °C. This composite electrode also demonstrated exceptional durability under harsh conditions. After 40 thermal cycles between 300 and 600 °C, the ASR of NTE-BSCF decreased by 22%, whereas that of BSCF increased by 47%. During a 600-h long-term stability test at 550 °C, the ASR of NTE-BSCF decayed by only 3.7%, significantly lower than the 23% decay for BSCF. Even after two years of exposure to the ambient atmosphere, the electrode performance remained stable, fully demonstrating the robustness imparted by the oxide wedging.

In recent years, the single-atom trapping/anchoring effect achieved *via* mechanical mixing has also been utilized to modify CFC cathodes. Zhuang *et al.* proposed a novel reverse atom trapping strategy, using a simple and scalable mechanical mixing method to precisely regulate the surface stoichiometry of the perovskite oxide (La_0.6_Sr_0.4_)_0.95_Co_0.2_Fe_0.8_O_3−*δ*_ (LSCF095) for use as a high-performance CFC cathode.^221^ This method involved co-calcining LSCF095 with acidic MoO_3_ at high temperature, triggering an acid–base reaction that selectively extracted Sr atoms from the LSCF095 surface. This process created Sr single-atom vacancies and concomitant oxygen vacancies, while the trapped Sr combined with MoO_3_ to form SrMoO_4_ (Fig. 13f–i). Although SrMoO_4_ is electrochemically inactive, it increased the stability of the electrode material. The modified composite (Sr_vac_/LSCF095) exhibited significantly enhanced lattice oxygen redox activity and suppressed Sr surface segregation, leading to improved ORR kinetics and electrochemical performance. A single cell based on Sr_vac_/LSCF095 achieved a PPD of approximately 350 mW cm^−2^ at 600 °C, higher than that of the pristine LSCF095-based cell. This mechanical mixing strategy not only enhanced the initial performance but also improved the long-term stability of the cathode. The Sr vacancies effectively inhibited the formation of insulating SrO islands, a common degradation mechanism in perovskite cathodes, enabling stable operation for over 1000 h at a constant voltage of 0.7 V. Similarly, Yu *et al.* employed a reverse atom trapping strategy based on mechanical mixing to modify the surface of the Pr_0.4_Sr_0.6_CoO_3−*δ*_ (PSC) perovskite cathode, enhancing its electrochemical performance and stability in LT-CFCs.^222^ This approach involved uniformly mixing PSCs with acidic WO_3_ powder *via* mechanical mixing followed by high-temperature calcination. The interfacial acid–base reaction drove the selective migration of Sr atoms from the PSC surface into WO_3_, resulting in PSCs with abundant Sr/O vacancies (V-PSC), while simultaneously forming SrWO_4_ (SWO) and a small amount of the Sr(Co/W)O_3_ (SCWO) perovskite phase. To investigate the effect of different WO_3_ amounts on reverse atom trapping, varying mass ratios (1%, 2%, and 3%) of WO_3_ were co-sintered with PSCs to obtain surfaces with different concentrations of Sr defects, denoted as V-PSC/1W, V-PSC/2W, and V-PSC/3W, respectively. The modified V-PSC cathodes exhibited significantly enhanced ORR activity due to increased surface oxygen vacancy concentration and enhanced lattice oxygen redox activity. In anode-supported single-cell tests, V-PSC/2W demonstrated an excellent output performance of 0.356 W cm^−2^ in the low-temperature range of 450 °C, representing a 106.8% improvement compared to pristine PSCs. The introduced Sr/O vacancies modulated the oxygen 2p band, lowered the energy barrier of the ORR rate-determining step, and significantly enhanced the reaction kinetics. Concurrently, the Sr/O vacancies effectively suppressed Sr segregation by increasing the thermodynamic energy barrier for Sr migration to the surface. During a 260-h stability test at 600 °C, the degradation rate of V-PSC/2W was only 1.82%, much lower than the 10.38% for pristine PSCs. Zhao *et al.* anchored single-atom Ru on the BaCe_0.125_Fe_0.875_O_3−*δ*_ (BCF) perovskite cathode to enhance CFC performance.^223^ This method involved ball-milling BCF powder with different mass percentages (1–3 wt%) of RuO_2_, followed by high-temperature calcination at 950 °C. This process enabled the thermal migration and atomic-level trapping of Ru species on the BCF surface, forming a unique four-coordinate Ru–O–Fe configuration. The optimized 2Ru-BCF (2 wt% Ru) cathode exhibited exceptional electrocatalytic activity for the proton-involved oxygen reduction reaction (P-ORR), attributed to the enhanced intrinsic activity of the single-atom Ru sites. In anode-supported single-cell tests, 2Ru-BCF demonstrated excellent power output in the low-temperature range. At 550 and 600 °C, its PPD reached 0.58 and 0.97 W cm^−2^, respectively, significantly higher than those of BCF at the corresponding temperatures. Furthermore, the strong metal–support interaction between the single-atom Ru and the BCF carrier ensured excellent operational stability. The 2Ru-BCF cathode remained stable without performance degradation or Ru atom aggregation after 200 h of continuous operation at 550 °C at a constant voltage of 0.7 V.

#### 3.5.2 Design and applications of electrolyte materials

A further mechanical mixing strategy has created unique opportunities for the development of electrolyte materials, enabling overall performance that far surpasses that of their individual parent materials.^224^ This advantage originates from the rich chemical and physical properties of the newly generated interfaces.^225^ For example, composite transition metal oxides have been adopted as sintering aids to address problems in which traditional electrolytes require high-temperature sintering for densification and suffer from issues such as Ba volatilization, dopant segregation and electrode structure damage due to high temperatures. This approach reduces sintering temperatures to minimize high-temperature side effects, while preserving high ionic conductivity through optimized additive content, ultimately enabling the device to demonstrate excellent electrochemical performance at low temperatures. However, this strategy still entails risks, including potential electronic leakage and complex effects of sintering aid distribution on grain boundary resistance.^226–229^ Meanwhile, mechanical mixing can achieve unexpected beneficial effects through the coupling of interface regulation and multiphase functional synergy. During mechanical mixing, for instance, potential element interdiffusion may cause lattice mismatch at heterogeneous interfaces that induces tensile strain, thereby increasing oxygen vacancy concentration and providing active sites for ionic migration.^230,231^ Moreover, the high defect density in interface regions offers fast migration channels for protons and oxygen ions, enhancing the conductivity of composite electrolytes by several times compared to pure-phase materials. Additionally, two-phase interfaces significantly promote ionic conduction through the formation of space charge layers, reduction of defect formation energy and lowering of diffusion barriers, thereby leading to remarkable improvements in the performance of composite electrolytes.^232,233^

**Fig. 14:**
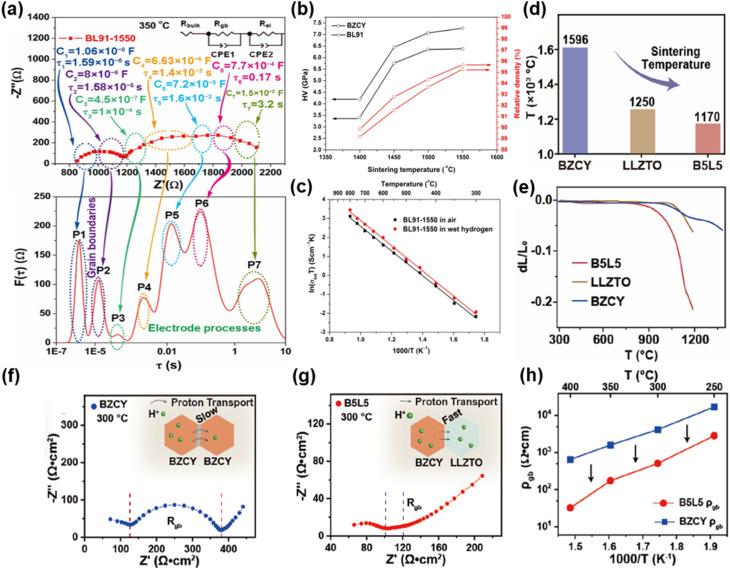
(a) Complex-plane impedance diagrams and the corresponding distribution of relaxation time plots of BL91 electrolyte sintered at 1550 °C measured under a wet hydrogen atmosphere at 350 °C. (b) Relative densities and Vickers hardness of BZCY and BL91 electrolytes as a function of sintering temperature. (c) Conductivity characteristics of BL91 sintered at 1550 °C measured in air and wet hydrogen atmospheres. Reproduced with permission.^234^ Copyright 2020, Wiley-VCH GmbH. (d and e) Comparison of sintering temperatures (d) and conventional heating sintering curves (e) of BZCY, LLZTO, and B5L5 samples. (f and g) EIS curves of Ni|B5L5|Ni (f) and Ni|BZCY|Ni (g) symmetric cells measured in wet H_2_ under OCV conditions at 300 °C. (h) Grain boundary resistance of BZCY and B5L5 in the temperature range of 250–400 °C. Reproduced with permission.^235^ Copyright 2024, American Chemical Society.

Specifically, our group addressed the challenges of poor sinterability and low electrical conductivity in BZCY PCE by introducing a composite approach through the addition of 10 wt% LSGM, resulting in the formation of the 90BZCY–10LSGM (BL91) (Fig. 14a–c).^234^ In this strategy, LSGM acted as a grain-boundary pinning phase to restrict grain-boundary mobility, thereby improving sintered density and conductivity. XRD results confirmed that no reaction occurred between BZCY and LSGM after sintering at 1400–1550 °C. Meanwhile, the BL91 composite exhibited a higher relative density of 95.7% when sintered at 1550 °C, along with a Vickers hardness of 7.27 GPa and a total conductivity of 8.4 × 10^−3^ S cm^−1^ at 600 °C in wet H_2_, all of which are substantially improved compared to pure BZCY (Fig. 14b and c).

Impedance analysis revealed that BL91 substantially reduced both ohmic and interfacial polarization resistances, demonstrating that the composite formation optimizes the microstructure and defect chemistry *via* grain-boundary pinning to overcome the limitations of PCEs. It is important to note that for the first time, we utilized the combination of DRT and EIS as an *in situ* characterization tool to distinguish the different grain boundary regions of the two electrolyte materials, providing a means for the next more detailed work, as depicted in Fig. 14a. The anode-supported cell with a BL91 electrolyte (≈50 µm) delivered a PPD of 220 mW cm^−2^ at 600 °C, with an OCV of 1.10 V and an ohmic resistance of 0.62 Ω cm^2^. This study provides a theoretical basis for understanding the enhancement mechanisms of composite electrolytes system and supports the rational design of high-performance materials.

Researchers have already improved the performance of electrolyte materials *via* mechanical mixing strategies that utilize the synergistic effect of dual-ion conduction and interface regulation. Pan *et al.* addressed the critical challenge of high sintering temperatures (up to 1596 °C) and poor grain-boundary conductivity in BZCY proton conductors, which hinder their applicability in LT-CFCs. The team developed a mechanical mixing approach, combined with ultrafast high-temperature sintering (UHS), to fabricate a new series of composite proton conductors. These composites, denoted as B_10−*x*_L_*x*_ (where 0 < *x* < 10 represents the mass percentage of the lithium-ion conductor Li_4.4_La_3_Zr_1.4_Ta_0.6_O_12_, LLZTO), consist of the proton conductor BZCY and LLZTO. Using this method, a specific composite with an equal mass ratio (*x* = 5), namely 50% BZCY – 50% LLZTO (referred to as B5L5), was successfully synthesized. This strategy took advantage of the low melting point of LLZTO to form a continuous conformal framework around BZCY particles. The design mechanistically reduced grain-boundary resistance, as the soft LLZTO phase smoothed the sharp interfaces of BZCY, thereby minimizing energy barriers for proton migration (Fig. 14d–h). First-principles calculations further confirmed that LLZTO facilitates proton-lithium ion exchange, enhancing conductivity through defect mediation. Experimentally, the B5L5 composite sintered at 1170 °C, which is 426 °C lower than pure BZCY (Fig. 14d and e), achieved a proton conductivity of 0.028 S cm^−1^ at 600 °C. This value is 4.5 times higher than that of BZCY, with grain-boundary resistance reduced by 70% (Fig. 14f–h).^235^ This work demonstrates how mechanical mixing optimizes the microstructure and defect chemistry by utilizing LLZTO both as a sintering aid and a proton-conductive phase, thereby advancing the design of PCEs.

Beyond the proton-conducting composites discussed above, another important class of electrolyte materials for LT-CFCs is based on semiconductor-ionic conductor heterostructures. These composites strategically combine a semiconducting phase with an ionic conducting phase to form heterojunctions at their interfaces. The unique properties of these semiconductor-ionic heterojunctions, including energy band alignment, space-charge layer formation, and localized defect accumulation, can dramatically enhance ionic transport while effectively suppressing electronic short-circuiting, enabling superionic conductivity at reduced temperatures.^87,236–241^ Among these, ceria-based semiconductor-ionic composites represent a particularly promising family of electrolyte materials. For instance, Ma *et al.* developed a novel synthetic route to produce SDC nanowires using citric acid as a complexing agent, followed by the incorporation of 20 wt% Na_2_CO_3_ to form a nanocomposite electrolyte.^239^ This template- and surfactant-free method enabled gram-scale production of uniform SDC nanowires with high aspect ratios (length >10 µm; diameter 100–200 nm) and a polycrystalline structure with an average crystallite size of approximately 7 nm. To address the thermal stability concern of nanomaterials at CFC operating temperatures, the authors employed a “nanocomposite” strategy where amorphous Na_2_CO_3_ served as a secondary phase to inhibit grain growth, preserving the nanowire architecture even after sintering at 700 °C. A single cell with the configuration NiO-SDC|SDC-Na_2_CO_3_|lithiated NiO was fabricated by co-pressing and subsequent sintering at 700 °C, resulting in an electrolyte layer thickness of approximately 200 µm. The cell was tested with humidified hydrogen (3% H_2_O) as the fuel and static air as the oxidant. Under these conditions, the SDC nanowire/Na_2_CO_3_ composite electrolyte achieved PPDs of 160, 316, 417, and 522 mW cm^−2^ at 450, 500, 550, and 600 °C, respectively. This performance significantly outperformed conventional GDC electrolytes of similar thickness (*e.g.*, 130 mW cm^−2^ at 550 °C and 220 mW cm^−2^ at 600 °C). The enhanced performance was attributed to the long continuous grain-boundary conduction paths provided by the one-dimensional nanowire structure and the stabilizing effect of the carbonate phase, which also aided densification under operating conditions.

As a versatile and scalable materials design strategy, mechanical compounding shows significant potential for advancing high-performance electrolytes capable of supporting efficient and durable LT-CFC operation. Achieving more uniform mixing of the two phases through cost-effective methods will be a key focus of future research.^242–244^

#### 3.5.3 Design and applications of anode materials

Mechanical mixing (such as ball milling) is the most common method for preparing Ni-based metal ceramic anode materials for CFCs. Researchers usually prepare NiO and electrolyte (BaCe_0.7_Zr_0.1_Y_0.1_Yb_0.1_O_3−*δ*_, BaZr_0.4_Ce_0.4_Y_0.1_Yb_0.1_O_3_, *etc.*) powders separately, and then mechanically mix the two in a certain proportion.^26,32^ In actual operation, a single cell with a mixture of NiO and electrolyte as the anode generally needs to be reduced in a hydrogen atmosphere at a given temperature for a period of time to reduce NiO to metallic Ni, and finally obtain a Ni-based metal ceramic anode.

**Fig. 15:**
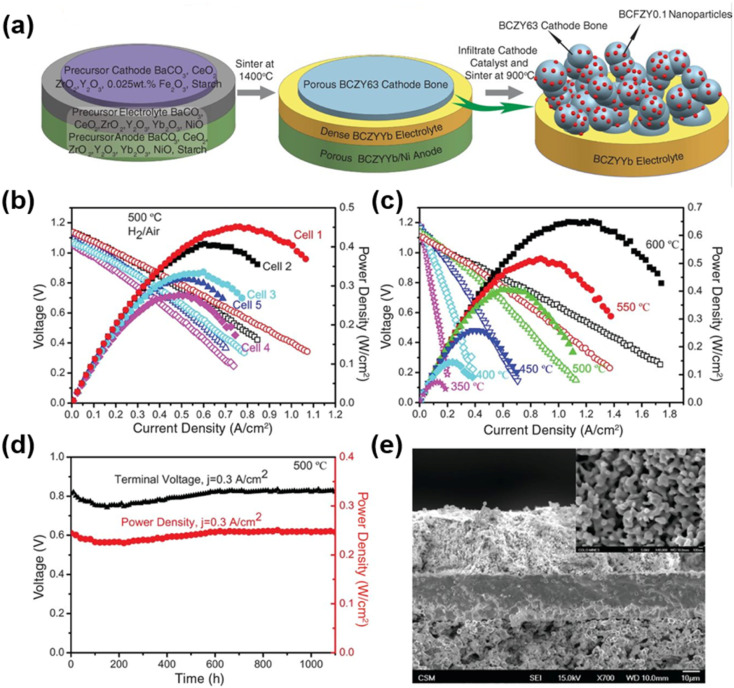
(a) Schematic illustration of the fabrication and structure of PCFC button cells. (b–e) Performance and microstructure of selected cells under H_2_ and air operation. Reproduced with permission.^26^ Copyright 2015, The American Association for the Advancement of Science.

Duan *et al.* prepared various anode materials using the mechanical mixing method, including 40 wt% BCZYYb + 60 wt% NiO (BCZYYb = BaCe_0.7_Zr_0.1_Y_0.1_Yb_0.1_O_3−*δ*_), 45 wt% BZY20 + 55 wt% NiO (BZY20 = BaZr_0.8_Zr_0.2_O_3−*δ*_), and 40 wt% BCZY63 + 60 wt% NiO (BaCe_0.6_Zr_0.3_Y_0.1_O_3−*δ*_).^26^ Cells based on these anode materials demonstrated excellent electrochemical performance and long-term stability below 600 °C. Under hydrogen fuel conditions, the cell with BCZYYb as the electrolyte achieved a PPD of 455 mW cm^−2^ at 500 °C, while maintaining approximately 100 mW cm^2^ even at a lower temperature of 350 °C. When methane was directly used as fuel, the BZY20-based cell exhibited peak power densities of 142, 215, and 290 mW cm^2^ at 500, 550, and 600 °C, respectively, with no significant degradation observed after continuous operation for over 1400 hours at 500 °C. The cells maintained stable OCV and output performance during prolonged testing, and microstructural analysis confirmed intact electrode/electrolyte interfaces without delamination or carbon deposition (Fig. 15). This study significantly enhanced the output capability and fuel adaptability of PCFCs in the low-temperature range through optimized anode composition and the solid-state reaction sintering process, providing crucial technical support for their application in low-temperature direct hydrocarbon fuel cells.

### 3.6 Impregnation strategy

Impregnation is a method currently used to improve the electrocatalytic performance of CFC electrodes. Researchers generally prepare a metal precursor salt solution in a certain proportion, then impregnate the salt solution on a porous electrode substrate, and then conduct a certain heat treatment to finally obtain a multiphase electrode with the original porous electrode material as the carrier and the impregnated metal or metal oxide particles evenly distributed on it.

#### 3.6.1 Design and applications of cathode materials

**Fig. 16:**
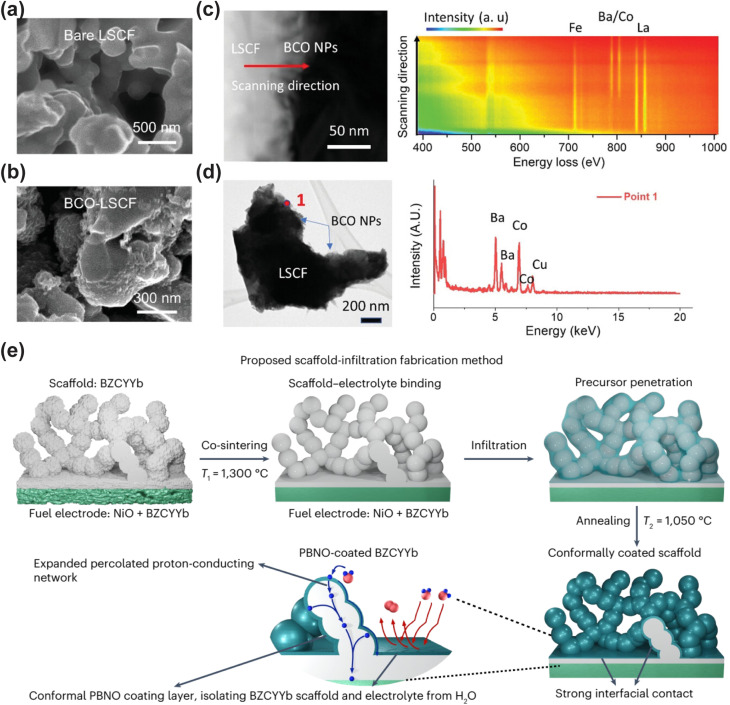
(a) SEM image of the bare LSCF air electrode. (b) SEM image of the BCO-LSCF air electrode. (c) A cross-sectional STEM image of BCO nanoparticles on a dense LSCF pellet and EELS spectra along the red line. (d) TEM image of an LSCF grain covered by BCO nanoparticles and the EDX profile of surface particles (point 1). Reproduced with permission.^245^ Copyright 2021, Wiley-VCH GmbH. (e) Schematic illustration of the proposed fabrication method of PCECs with the CCS-based air electrode design that can improve stability against steam. The PCEC comprises a dense electrolyte and scaffold structure based on doped barium cerates BZCYYb, BaZr_0.4_Ce_0.4_Y_0.1_Yb_0.1_O_3−*δ*_ (BZCYYb4411) or BaCe_0.8_Y_0.2_O_3−*δ*_ (BCY20), an air electrode comprising Pr_1.8_Ba_0.2_NiO_4.1_ (PBNO) and a fuel electrode consisting of a NiO and electrolyte composite. Reproduced with permission.^249^ Copyright 2025, Springer Nature.

In recent years, impregnation has been widely adopted as an effective electrode modification strategy to enhance the catalytic activity, durability, and anti-contamination capability of CFC cathodes. Zhou *et al.* constructed a BCO nanoparticle coating approximately 30 nm thick on the surface of a LSCF cathode *via* an impregnation method, which significantly improved the ORR activity of the cathode material (Fig. 16a–d).^245^ EIS revealed that the ASR of the BCO-coated LSCF (BCO-LSCF) electrode was only 0.16 Ω cm^2^ at 600 °C, approximately 30% that of the bare LSCF electrode. At 500 °C, the ASR of BCO-LSCF was also markedly reduced to 0.84 Ω cm^2^ from 3.86 Ω cm^2^ for bare LSCF. Single-cell tests further confirmed the superior performance of the BCO-LSCF cathode: the PPDs at 600 and 500 °C reached 1.16 and 0.41 W cm^−2^, respectively, substantially higher than the 0.65 and 0.21 W cm^−2^ achieved with the bare LSCF cathode. Under high-temperature and high-humidity conditions, the BCO coating significantly enhanced the stability of the LSCF electrode. After a 500-h test at 600 °C with 30% steam, the degradation rate of the BCO-LSCF electrode was only 4.1 × 10^−4^ Ω cm^2^ h^−1^, much lower than the 1.3 × 10^−3^ Ω cm^2^ h^−1^ observed for the bare LSCF electrode. ECR measurements and associated analysis indicated that the BCO coating not only accelerated the surface oxygen exchange kinetics but also effectively suppressed Sr segregation, thereby enhancing electrode durability. Similarly, Niu *et al.* applied an impregnation method to deposit a multiphase (MP) catalyst coating composed of Ba_1−*x*_Co_0.7_Fe_0.2_Nb_0.1_O_3−*δ*_ (BCFN) and BaCO_3_ on a conventional LSCF cathode, significantly improving its ORR activity and resistance to Cr poisoning.^246^ This MP catalyst coating uniformly covered the LSCF surface with a thickness of approximately 20–50 nm. Electrochemical tests demonstrated that the ASR of the MP catalyst-coated LSCF cathode at 600 °C was 0.175 Ω cm^2^, considerably lower than that of the bare LSCF. A single cell employing the MP catalyst-coated LSCF cathode achieved a PPD of approximately 0.3 W cm^−2^ at 600 °C. Mechanistic studies revealed that the BCFN phase provided highly active sites for the ORR and facilitated oxygen adsorption and dissociation, while the BaCO_3_ phase acted as a physical barrier, effectively inhibiting Sr segregation and the deposition of Cr-containing species. Zhang *et al.* employed a double perovskite material PBSCF as the substrate and constructed a PFC-coated PBSCF (PFC-PBSCF) composite cathode by impregnating a Ba/Sr-free Pr_0.9_Fe_0.7_Co_0.3_O_3_ (PFC) nanocatalyst onto its surface.^247^ This strategy not only significantly enhanced the ORR activity of the electrode but also substantially improved its durability in environments containing Cr and water vapor. In symmetric cell tests, the PFC-PBSCF electrode exhibited an ASR of only 0.81 Ω cm^2^ at 600 °C, markedly lower than the 1.04 Ω cm^2^ of bare PBSCF. In single cell tests, the cell with the PFC-PBSCF cathode achieved a PPD of approximately 0.85 W cm^−2^ at 600 °C, even when exposed to air containing 3% steam and a Cr contamination source, significantly outperforming the bare PBSCF cathode (∼0.58 W cm^−2^). Raman spectroscopy and computational analysis further elucidated the protective mechanism of the PFC coating: it effectively suppressed the formation of insulating (Ba_1−*x*_Sr_*x*_)CrO_4_ phases resulting from reactions between Ba/Sr and Cr species, and reduced the deposition of Cr_2_O_3_ on the electrode surface. Moreover, the PFC coating maintained high catalytic activity and structural stability in humid atmospheres, significantly mitigating performance degradation caused by steam-enhanced BaO/SrO segregation. Zheng *et al.* introduced oxygen-vacancy-rich PrO_*x*_ nanoparticles into a PrNi_0.7_Co_0.3_O_3−*δ*_ (PNC) scaffold *via* the impregnation method, constructing a PNC-PrO_*x*_ composite oxygen electrode with a synergistic surface and bulk phase catalysis.^248^ A single cell based on this composite electrode demonstrated excellent electrochemical performance, achieving a PPD of 1.56 W cm^−2^ at 600 °C, an 83% improvement compared to the unmodified PNC electrode (0.85 W cm^−2^). In terms of durability, the PNC–PrO_*x*_ electrode exhibited an extremely low degradation rate during an 80 h potentiostatic test and maintained structural stability without interfacial delamination or active layer degradation during thermal and potential cycling tests. Structural characterization and mechanistic analysis indicated that the heterogeneous structure formed by PrO_*x*_ nanoparticles and nanowires significantly increased the density of active sites and optimized pathways for oxygen adsorption and proton conduction. XPS and EPR analyses confirmed that the introduction of PrO_*x*_ substantially increased the surface oxygen vacancy concentration.

Conventional planar contact electrode structures often suffer from severe performance degradation during long-term operation at high steam concentrations and large current densities, primarily due to the hydrothermal instability of electrolyte and electrode materials and poor interfacial contact. To address this issue, Tian *et al.* proposed an innovative impregnation strategy to construct a conformally coated scaffold (CCS) architecture, which significantly enhanced the durability and electrochemical performance of CFCs under harsh conditions.^249^ This approach first involved fabricating a porous proton-conducting scaffold (*e.g.*, BZCYYb) on a dense electrolyte surface *via* a co-sintering process, followed by uniformly coating the scaffold with Pr_1.8_Ba_0.2_NiO_4.1_ (PBNO), through wet chemical impregnation, forming a continuous and defect-free protective layer, as illustrated in Fig. 16e. This coating not only effectively shielded the electrolyte from steam corrosion but also provided excellent pathways for proton, oxygen ion, and electron transport, thereby enhancing active sites for electrode reactions and proton transport kinetics. A single cell employing the PBNO-CCS cathode achieved PPDs of 181, 360, 629, and 1160 mW cm^−2^ at 450 °C, 500 °C, 550 °C, and 600 °C, respectively. Notably, at 600 °C, the cell power density surpassed that of most cobalt-containing CFC electrodes, highlighting the significant advantages of the CCS structure in improving interfacial contact, promoting proton diffusion, and expanding the electrochemically active area. Furthermore, the CCS-structured single cell demonstrated exceptional stability, operating for 1018 h without performance degradation in an atmosphere containing 10% H_2_O/air, showcasing unprecedented long-term durability.

In summary, the impregnation strategy, by introducing highly catalytically active nanoparticle coatings without altering the bulk electrode structure, has achieved remarkable enhancements in the performance and durability of conventional cathode materials in both oxygen-ion and proton-conducting systems.

#### 3.6.2 Design and applications of electrolyte materials

While bulk composition determines the intrinsic transport properties of electrolyte materials, their practical performance in LT-CFCs is often dictated by surface and interfacial characteristics. High interfacial resistance, inadequate surface exchange kinetics, and poor sinterability frequently limit the effectiveness of otherwise promising electrolyte compositions. Conventional bulk modification approaches, such as doping or composite formation, cannot always address these localized limitations without compromising other desirable properties. The impregnation strategy offers a complementary and highly targeted solution.^250–252^ When infiltration solutions penetrate porous scaffolds, previously disconnected particles become interconnected, promoting densification of the material at lower temperatures. Studies on acid-treated electrolyte interfaces have profoundly revealed the core influence of interface states on CFC performance.^253^ To this end, it is essential to explore material design strategies that optimize interfacial properties by regulating intrinsic characteristics such as vacancy concentrations, thereby achieving comprehensive performance enhancement.^254^

**Fig. 17:**
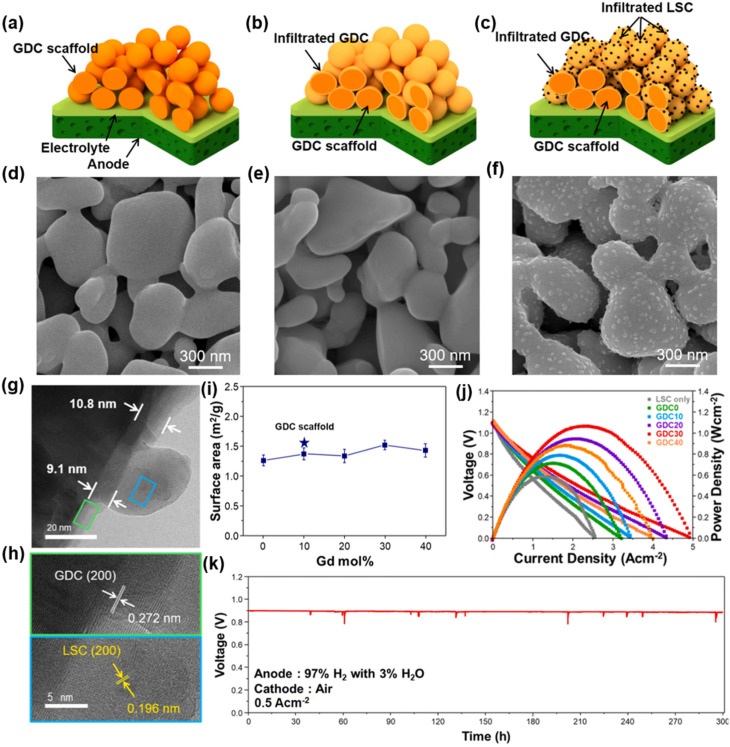
(a–c) Schematics of GDC scaffold fabrication: (a) pristine GDC scaffold, (b) GDC30-infiltrated, and (c) LSC/GDC30-infiltrated. (d–f) SEM images of the corresponding scaffolds in (a–c). (g and h) HR-TEM images of the LSC/GDC30-infiltrated GDC scaffold. (i) Specific surface area comparison of the pristine GDC scaffold and GDC-infiltrated scaffolds with different Gd doping ratios. (j) *I*–*V*–*P* curves of LSC/GDC-infiltrated cells with Gd doping ratios ranging from 0 to 40 mol%. (k) 300-h galvanostatic stability test curve of the LSC/GDC30-infiltrated cell. Reproduced with permission.^251^ Copyright 2021, Elsevier.

Kim *et al.* tackled the issue of inadequate surface defect chemistry in GDC electrolytes, which limits ion transport and interfacial reactivity in LT-CFCs.^251^ They employed an infiltration strategy to deposit conformal GDC layers with a thickness of 7–11 nm and controlled Gd^3+^ doping (0–40 mol%) onto porous GDC scaffolds *via* a wet-chemical processes, as shown in Fig. 17a–h. This method allowed precise tuning of surface oxygen vacancies without disrupting the scaffold structure and proved more scalable than complex vacuum-based deposition techniques.

The process involved infiltrating Gd(NO_3_)_3_/Ce(NO_3_)_3_ solutions into the scaffold, followed by drying and calcination, resulting in defect-engineered layers where Gd^3+^ substitution for Ce^4+^ systematically increased oxygen vacancy concentrations and enhanced electrolyte surface reactivity. The formation of fine grains increased grain boundary density, which further enriched oxygen vacancies and thereby improved surface activity (Fig. 17i). Mechanistically, these tailored vacancies promoted ion incorporation and charge transfer at the electrolyte–cathode interface. On employing a GDC electrolyte (≈10 µm) scaffold modified with an infiltrated 7–11 nm GDC layer (30 mol% Gd), the anode-supported cell achieved a PPD of 1.07 W cm^−2^ at 650 °C, which is 1.81 times higher than that of the untreated cell, along with a low polarization resistance of 0.39 Ω cm^2^. The cell also demonstrated excellent stability during a 300-h galvanostatic test at 650 °C and a current density of 0.5 A cm^−2^ (Fig. 17j and k). This study successfully realized the customized design of oxygen vacancy concentration at the electrolyte interface through an infiltration strategy, significantly reducing interfacial resistance with electrode materials. It is reasonable to believe that further promotion of this work will contribute to overall performance improvement of LT-CFCs.^255,256^

#### 3.6.3 Design and applications of anode materials

In order to overcome the inherent defects of sintering, nitriding, and activity degradation of traditional nickel based ceramic metal anodes under an ammonia atmosphere and at high temperature, many researchers have accurately impregnated the precursor solution of the active component into the pre formed porous electrolyte skeleton (such as SDC or BZCYYb), avoiding the high-temperature process of electrode and electrolyte co-sintering, and effectively suppressing the aggregation and coarsening of active metal nanoparticles. More importantly, by combining with the *in situ* precipitation strategy, the impregnation method can construct alloy nanoparticles (*e.g.*, NiCo and Fe, Ru–Cu–Ni) that are strongly coupled with the substrate.^112,257,258^ This not only optimizes the intrinsic activity of NH_3_ decomposition through multi-component synergistic effects, but also significantly enhances the microstructure stability and sintering resistance of the anode through strong metal carrier interactions, ultimately achieving a synergistic improvement in cell performance and durability.

**Fig. 18:**
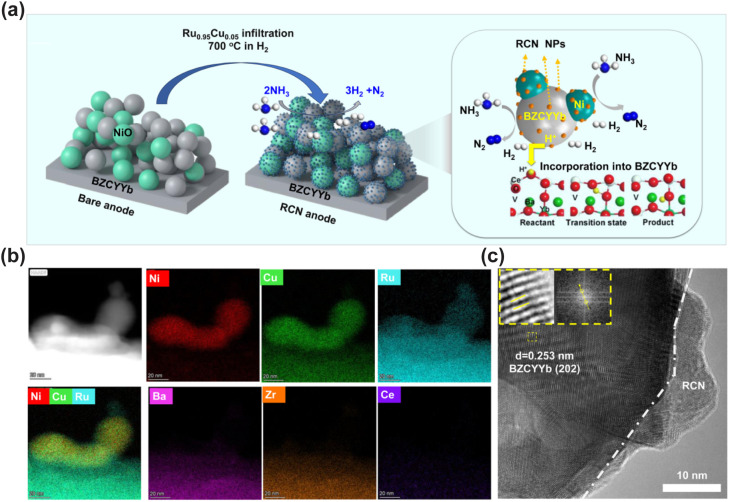
(a) Schematic of a fuel electrode modified with Ru_0.95_Cu_0.05_Ni_*x*_ nanoparticles when operated with NH_3_. (b) STEM-EDX mapping of an RCN anode and (c) HRTEM images of the RCN anode and the corresponding FFT pattern. Reproduced with permission.^258^ Copyright 2024, The Royal Society of Chemistry.

The impregnation method, as an efficient strategy for electrode interface modification, can *in situ* form highly active and stable catalytic structures on the surface and near-surface regions of electrodes by introducing trace amounts of precursor solutions into the porous electrode framework and subsequent thermal treatment. Zhang *et al.* employed a one-step impregnation method to introduce Ru_0.95_Cu_0.05_ nitrate solution onto the surface of Ni-BZCYYb anodes, which, after reduction at 700 °C, reacted with the Ni nanoparticles leached from the anode to self-assemble into Ru–Cu–Ni (RCN) heterostructure catalysts (Fig. 18).^258^ This RCN structure significantly optimized the interface properties of the anode: in terms of catalytic activity, due to the strong activation ability of Ru sites towards NH_3_ and the synergistic effect of Cu sites in promoting N_2_ desorption, the NH_3_ conversion rate of the anode at 550 °C increased from 46% for the bare anode to 98%, while the polarization resistance of the symmetrical cell was significantly reduced to 8.97 Ω cm^2^ (62.02 Ω cm^2^ for the bare anode). At the single-cell level, this modification led to a PPD of 0.732 W cm^−2^ at 550 °C. In terms of durability, the RCN structure effectively suppressed the coarsening, nitridation of Ni particles, and the formation of micro-pores, resulting in a voltage decay rate of only 0.016 V per 100 h for the cell under a constant current discharge of 0.2 A cm^−2^ at 550 °C for 100 hours, which was far superior to the 0.095 V per 100 h of the bare anode. Regarding thermal stability, after 31 thermal cycles in the range of 550–700 °C, the PPD of the RCN anode only slightly decreased from 1.542 W cm^−2^ to 1.411 W cm^−2^, and both the polarization resistance and ohmic resistance remained stable, demonstrating excellent thermal shock resistance. DFT calculation results further revealed that the adsorption energy of nitrogen atoms on the RCN (111) surface (−5.15 eV) was lower than that on the Ni (111) surface (−5.27 eV), and the energy barrier for associative desorption of nitrogen on RCN was 1.67 eV, lower than 1.84 eV for Ni, effectively alleviating the poisoning of the anode by nitrogen species.

In summary, the RCN heterostructure constructed by the impregnation method regulated the surface reaction pathways at the atomic scale, optimized the catalytically active sites at the microscale, and significantly enhanced the comprehensive electrochemical performance and thermal-mechanical stability of the electrode below 600 °C, providing a key material basis for the development of low-temperature, high-durability ammonia PCFCs.

### 3.7 Self-assembly

Self-assembly refers to the process in which a disordered system spontaneously forms an ordered structure through the interactions between its basic components, and this process does not require external intervention. This strategy enables precise design of material components to construct multifunctional nanocomposites. Traditional mechanical mixing methods often result in larger particle sizes, uneven distribution, and weaker interfacial interactions in composite materials, thereby limiting the output performance and longevity of CFCs. The interaction between different phases synthesized by the self-assembly strategy can enhance interfacial adhesion and generate composite materials with a more uniform distribution and smaller particle size.

#### 3.7.1 Design and applications of cathode materials

**Fig. 19:**
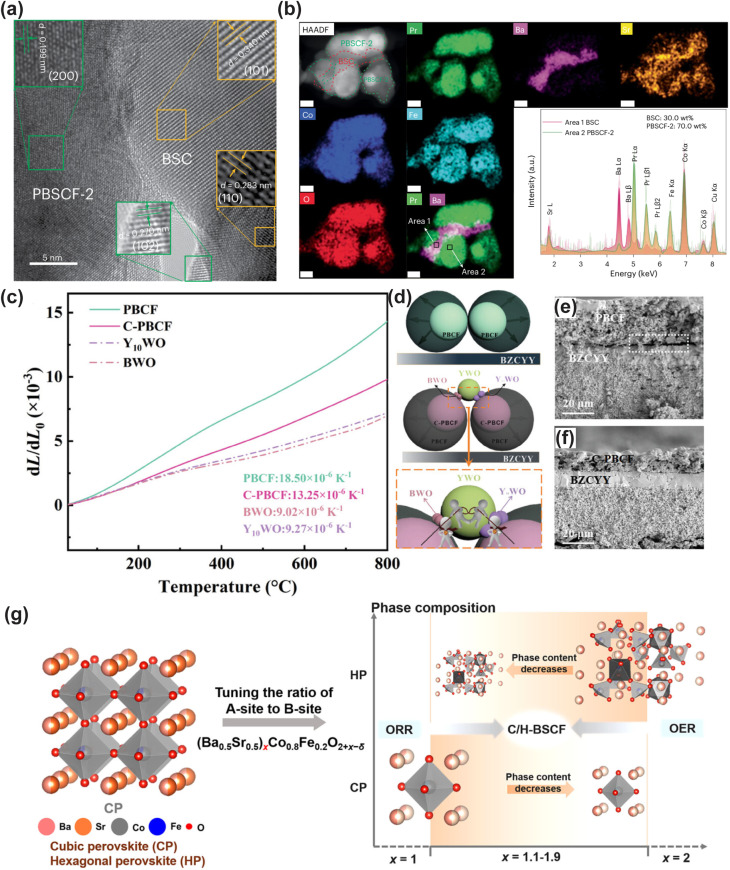
(a) HR-TEM image of BSC + PBSCF-2, which confirms that BSC + PBSCF-2 is composed of PBSCF-2 and BSC. (b) HAADF image and corresponding EDS mapping images, which further affirm that BSC + PBSCF-2 is composed of PBSCF-2 and BSC. The scale bar is 20 nm. Reproduced with permission.^259^ Copyright 2023, Springer Nature. (c) Thermal expansion curves of dense C-PBCF, PBCF, BWO, and Y_10_WO bar specimens from 30 to 800 °C in air. (d) Mechanism of thermo mechanical enhancement of TEC deflection in C-PBCF realized by the collaborative efforts of trilateral (BWO, Y_10_WO, and YWO). The SEM images of the cell using the PBCF cathode (e) and the C-PBCF cathode (f) after a single test. Reproduced with permission.^264^ Copyright 2024, Wiley-VCH GmbH. (g) The phase content-controlled hybrid electrode composed of cubic and hexagonal perovskites induced by tuning the ratio of A-sites to B-sites of cubic perovskite. Reproduced with permission.^265^ Copyright 2024, Springer Nature.

*In situ* self-assembly engineering of cathode materials represents a widely reported strategy for electrode modification. During cathode synthesis, all components are simultaneously formed within the same reaction environment without requiring additional processing. The interaction between different phases leads to enhanced contact and reduced particle size, ensuring intrinsic chemical synergy among the components. This facilitates a homogeneous distribution, expanded heterogeneous interfaces, enhanced TPBs, and robust interfacial bonding strength. Liu *et al.* reported a composite air electrode material, Ba_0.62_Sr_0.38_CoO_3−*δ*_-Pr_1.44_Ba_0.11_Sr_0.45_Co_1.32_Fe_0.68_O_6−*δ*_ (BSC + PBSCF-2), constructed *via* an *in situ* self-assembly strategy.^259^ This self-assembled composite electrode, comprising a double perovskite PBSCF-2 phase and a hexagonal BSC nanoparticle phase, was synthesized *via* a wet chemical method and crystallized at 765 °C. XRD Rietveld refinement and TEM/EDS analysis confirmed its chemical homogeneity and dual-phase coexistence. The uniform distribution of BSC nanoparticles within the PBSCF-2 matrix significantly enhanced the surface oxygen exchange rate and bulk oxygen ion diffusion capability (Fig. 19a and b). ECR analysis demonstrated that both the surface oxygen exchange and bulk oxygen diffusion coefficients for BSC + PBSCF-2 were substantially higher than those of the conventional PBSCF electrode. In terms of electrochemical performance, a single cell employing the BSC + PBSCF-2 cathode achieved PPDs of 1.64 W cm^−2^ in air and ∼2.0 W cm^−2^ in oxygen at 600 °C. At 450 °C, the power density remained as high as 0.77 W cm^−2^, significantly outperforming the conventional PBSCF-2 cathode (0.29 W cm^−2^). Furthermore, the cell delivered practical power densities of 0.34 W cm^−2^ and 0.10 W cm^−2^ at extremely low temperatures of 400 °C and 275 °C, respectively, demonstrating excellent low-temperature adaptability. The self-assembled electrode also exhibited superior interfacial adhesion strength and thermal expansion compatibility with the electrolyte. Peel strength tests revealed a 42% improvement in the adhesion strength between BSC + PBSCF-2 and the electrolyte compared to PBSCF-2. The TEC decreased from 23.4 × 10^−6^ K^−1^ for PBSCF-2 to 18.0 × 10^−6^ K^−1^ for the composite, effectively reducing the interfacial contact resistance and enhancing long-term stability. The single cell demonstrated exceptional operational stability, with a performance degradation rate below 0.07 mV h^−1^ during 100-h continuous operation at 400 °C. Our group successfully constructed BaFe_0.6_Ce_0.2_Sc_0.2_O_3−*δ*_ (BFCS) with a nano-heterostructure *via* a self-assembly strategy, achieving effective modulation of lattice oxygen activity.^260^ This strategy spontaneously forms a nanocomposite structure through a one-step high-temperature sintering process, consisting of approximately 80 wt% Fe-rich cubic phase BaFe_0.8_(Ce_0.3_Sc_0.7_)_0.2_O_3−*δ*_ and approximately 20 wt% Ce-rich orthorhombic BaCe_0.6_(Fe_0.5_Sc_0.5_)_0.4_O_3−*δ*_. HR-TEM and EDS confirmed the heterogeneous distribution of Fe and Ce elements at the nanoscale, forming intimately connected hetero-interfaces that significantly enhanced oxygen species migration and surface reaction kinetics. Electrochemically, a symmetric cell with the self-assembled BFCS electrode exhibited a very low ASR of 0.175 Ω cm^2^ at 600 °C, approximately one-third that of the parent BaFe_0.6_Ce_0.4_O_3−*δ*_ (BFC) electrode. A single cell assembled with the BFCS cathode delivered PPDs of 0.61 and 0.33 W cm^−2^ at 600 and 550 °C, respectively, and demonstrated good short-term operational stability over 70 h at 600 °C and 0.8 V. Song *et al.* developed a cobalt-free multiphase nanocomposite, Sr_0.9_Ce_0.1_Fe_0.8_Ni_0.2_O_3−*δ*_ (SCFN2), as a near-ideal cathode for CFCs *via* a high-temperature-induced self-assembly strategy.^261^ This material comprises four nanoscale phases: a primary tetragonal perovskite SCFN phase (T-SCFN, 77.2 wt%) and a secondary R–P SCFN phase (RP-SCFN, 13.3 wt%), along with surface-decorated NiO (5.8 wt%) and CeO_2_ (3.7 wt%) nanoparticles. This multiphase structure features intimate nanoscale contact that significantly increases the number of active sites for the ORR. The R–P phase promotes bulk oxygen diffusion; NiO nanoparticles enhance oxygen surface adsorption and dissociation, while CeO_2_ facilitates the migration of oxygen ions from the surface into the bulk. A single cell based on the SCFN2 cathode exhibited PPDs of 977, 702, 451, and 219 mW cm^−2^ at 600, 550, 500, and 450 °C, respectively. Furthermore, the symmetric cell ASR was only 0.072 Ω cm^2^ at 600 °C, indicating outstanding ORR catalytic activity. The material also demonstrated excellent thermal stability, good chemical compatibility with the SDC electrolyte, a moderate TEC (16.8 × 10^−6^ K^−1^), and operational stability for 560 h, attributable to the multiphase synergistic effects and nanoscale interface engineering achieved during self-assembly. Bai *et al.* proposed a Pr-induced *in situ* self-assembly strategy to successfully construct a composite cathode material with La_0.6_Sr_0.4_Co_*x*_Fe_*y*_O_3−*δ*_/PrCoO_3_/Co_3_O_4_/PrO_2_ (LSCFC-PI) multiphase heterojunctions, significantly enhancing its electrochemical performance and stability.^262^ This was achieved by impregnating the LSCFC precursor (a composite of LSCF and CoFe_2_O_4_) with a Pr(NO_3_)_3_ solution, followed by high-temperature calcination, which induced the decomposition of the secondary CoFe_2_O_4_ phase and promoted the reorganization of Pr, Co, and Fe elements, ultimately forming a uniformly distributed multiphase nanostructure. This structure creates tight hetero-interfaces at the nanoscale, not only significantly increasing the specific surface area (from 3.34 m^2^ g^−1^ to 12.16 m^2^ g^−1^) but also effectively suppressing Sr surface segregation and enhancing stability under a CO_2_ atmosphere. The LSCFC-PI cathode exhibited an ASR as low as 0.124 Ω cm^2^ at 600 °C. A single cell based on this cathode achieved a PPD of approximately 850 mW cm^−2^ at 600 °C. Moreover, operated at a constant voltage of 0.8 V for 100 h, the current density degradation rate was only 0.09% h^−1^, demonstrating exceptional long-term stability. The material also possessed superior CO_2_ tolerance, with an ASR degradation rate of only 6.5% in a CO_2_ atmosphere, far lower than the 21.0% for unmodified LSCFC. This is attributed to the Pr-induced core–shell structure and the provided acidic surface environment, effectively inhibiting the formation of the insulating SrCO_3_ phase. Huang *et al.* reported an Nb and Y co-doped cobalt-based double perovskite material, PrBaCo_1.8_Nb_0.1_Y_0.1_O_5+*δ*_ (PBCNY), which undergoes *in situ* self-assembly during high-temperature operation into a primary A-site deficient PrBa_1−*x*_Co_1.8_Nb_0.1−*x*_Y_0.1−*x*_O_5+*δ*_ parental phase and a secondary Ba_2_YNbO_6_ phase.^263^ This composite cathode exhibited an exceptionally low ASR of 0.24 Ω cm^2^ at 600 °C, along with significantly enhanced oxygen vacancy concentration, surface exchange, and bulk diffusion capabilities. In single-cell tests, a cell employing the PBCNY cathode achieved a PPD of 0.97 W cm^−2^ at 600 °C and stable operation for over 100 h, demonstrating remarkable structural and electrochemical stability. These improvements are ascribed to the *in situ* formed Ba_2_YNbO_6_ phase, which not only stabilizes the perovskite structure but also provides additional active sites, promoting the co-transport of oxygen ions and protons. Gao *et al.* constructed a PBCF + YWO composite cathode (C-PBCF) with a TEC gradient by co-sintering high-TEC PrBa(Co_0.7_Fe_0.3_)_2_O_5+*δ*_ (PBCF) and NTE Y_2_W_3_O_12_ (YWO), inducing the *in situ* formation of BaWO_4_ and Y_10_W_8_O_21_ transition phases at the interface (Fig. 19c–f).^264^ This structure not only effectively mitigates thermal stress but also optimizes ORR kinetics through the introduction of A-site deficiencies. This self-assembled composite cathode demonstrated outstanding electrochemical performance at 600 °C, achieving a PPD of 0.88 W cm^−2^, significantly superior to conventionally mixed cathodes. A single cell with the C-PBCF cathode maintained stable operation for 100 h at 600 °C and 0.7 V. This self-assembly strategy achieved graded regulation of the thermal expansion behaviour between the cathode and electrolyte, while the formation of transition phases suppressed the hygroscopicity of YWO, enhancing the chemical stability under realistic operating conditions. The cell performance showed no significant degradation after 65 rigorous thermal cycles, further validating the excellent durability of this structure under thermomechanical stress. Liu *et al.* successfully constructed a dual-phase composite electrode material C/H-BSCF, composed of cubic BSCF (C-BSCF) and hexagonal Ba_4_Sr_4_(Co_0.8_Fe_0.2_)_4_O_16−*δ*_ (H-BSCF).^265^ Unlike traditional methods involving physical mixing or tolerance factor mismatch-induced phase separation, this strategy enabled controllable adjustment of the two-phase content and promoted intimate interfacial contact and synergy between the phases by precisely tuning the A-site and B-site stoichiometry (Fig. 19g). In symmetric cell tests, the C/H-BSCF electrode exhibited a very low ASR of 0.26 Ω cm^2^ in humidified air at 600 °C, significantly superior to single-phase C-BSCF (0.48 Ω cm^2^) and H-BSCF (0.34 Ω cm^2^). DRT analysis indicated lower polarization resistance in the medium-frequency region for C/H-BSCF, suggesting significantly enhanced surface oxygen exchange and bulk ion migration rates. Characterization studies including X-ray Absorption Near Edge Structure (XANES), XPS, and TGA further revealed that the cubic phase dominates the oxygen activation process, while the hexagonal phase contributes abundant oxygen vacancies and excellent hydration capability, synergistically enhancing the electrode's triple conductivity and reaction kinetics. In single-cell tests, a cell with the C/H-BSCF cathode demonstrated a remarkable PPD of 1.67 W cm^−2^ at 600 °C, far exceeding those of single-phase C-BSCF (0.68 W cm^−2^) and H-BSCF (0.77 W cm^−2^). Furthermore, the PPDs remained high at 1.37 and 1.00 W cm^−2^ at 550 and 500 °C, respectively, indicating excellent low-temperature activity. Concurrently, the cell demonstrated excellent durability, operating stably for 200 h at a current density of 0.4 A cm^−2^.

In summary, the self-assembly strategy, leveraging differences in ionic radii and valence states among constituent elements, spontaneously forms dual-phase or multiphase composite air electrodes with high activity, stability, and multi-ion conduction capability. This provides a viable technological pathway for achieving efficient low-temperature energy conversion.

#### 3.7.2 Design and applications of electrolyte materials

Mechanical mixing, while widely used to fabricate composite electrolytes, often suffers from limited phase homogeneity, large domain sizes, and weak interfacial bonding between components. These drawbacks restrict the full utilization of hetero-interface synergistic effects and may even introduce undesired secondary phases or thermal expansion mismatch. The self-assembly strategy offers a transformative alternative by enabling the spontaneous formation of multiphase nanocomposites with intimately connected and uniformly distributed phases directly during synthesis. Through one-pot processes such as sol–gel or combustion synthesis, different phases crystallize simultaneously from molecular-level mixed precursors, yielding coherent hetero-interfaces, continuous three-dimensional conduction networks, and enhanced chemical compatibility between components.^266,267^

**Fig. 20:**
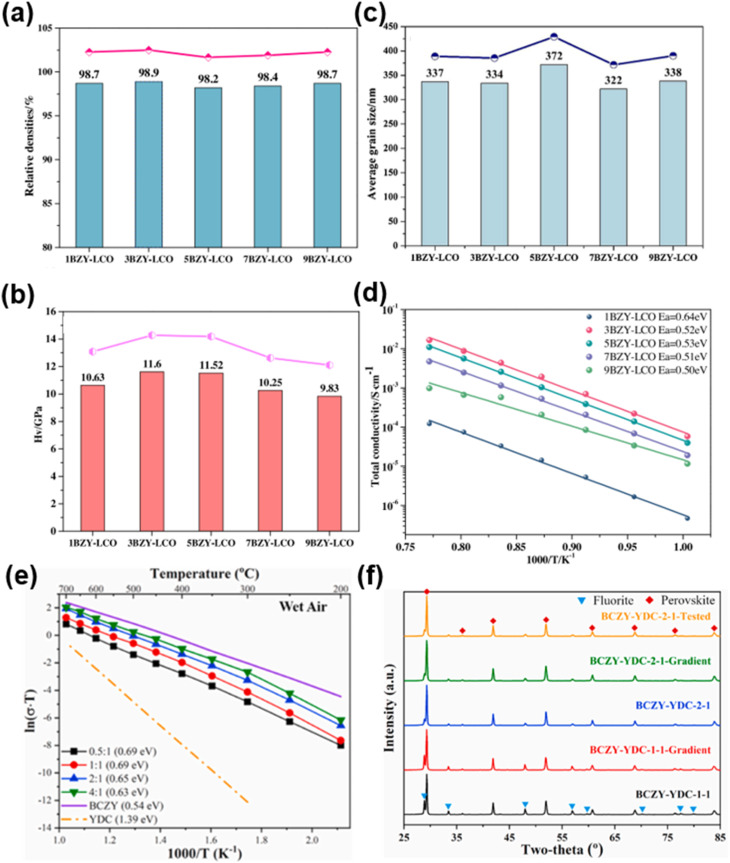
(a) Relative densities. (b) Vickers hardness. (c) Average grain sizes (*x*BZY-LCO composite powders). (d) Conductivity curves and activation energies (xBZY-LCO pellets sintered at 1450 °C, dry air). Reproduced with permission.^266^ Copyright 2021, Elsevier. (e) Conductivity-temperature dependence (BCZY-YDC pellets, wet air). (f) XRD patterns of tested BCZY-YDC-2-1 and BCZY-YDC-1-1/2-1 after gradient treatment (*vs.* fresh samples). Reproduced with permission.^267^ Copyright 2021, Elsevier.

Bao *et al.* addressed the critical challenges of pure BaZr_0.8_Y_0.2_O_3−*δ*_ (BZY) requiring a sintering temperature as high as 1700 °C and La_2_Ce_2_O_7_ (LCO) suffering from electron leakage under reducing atmospheres by adopting a one-step co-firing strategy to fabricate BZY-LCO composite electrolytes for LT-CFCs.^266^ This approach utilized the self-organization of BZY and LCO during a one-pot citric acid-nitrate combustion synthesis. Owing to its high sintering activity, LCO promoted densification through a dissolution–precipitation mechanism at 1450 °C, which is approximately 250 °C lower than that required for pure BZY. Meanwhile, the stable perovskite framework of BZY physically blocked the electron conduction pathways within LCO. The one-pot synthesis enabled the *in situ* formation of a homogeneous dual-phase network, in which the cubic perovskite BZY and cubic fluorite LCO phases demonstrated excellent chemical compatibility. This led to a synergistic interface that facilitated proton transport. The composite reached a densification level of up to 98.9% in the 3BZY–7LCO system (Fig. 20a–c) while EIS confirmed a reduction in grain boundary resistance. The high proton conductivity of BZY and the mixed ion conductivity of LCO synergistically enhanced the overall conductivity (Fig. 20d). Furthermore, such self-assembled composite electrolyte strategies help avoid phase separation and the formation of interfacial impurities, which are common issues in conventional mechanical mixing processes. The anode-supported cell with the 3BZY-7LCO composite electrolyte (≈20 µm) exhibited a PPD of 135 mW cm^−2^ and an OCV of 0.889 V at 600 °C. The cell also demonstrated stable operation during a 100-h test under constant current at 700 °C.

Zhao *et al.* developed a one-pot solid-state reactive sintering method to synthesize BaCe_0.5_Zr_0.4_Y_0.1_O_3−*δ*_-Ce_0.5_Y_0.5_O_2−*δ*_ (BCZY-YDC-2-1) composites for LT-CFCs. This novel technique successfully mitigated the poor homogeneity, large phase domains and interface impurities that often degrade performance in composites made by mechanical mixing.^267^ This strategy utilized *in situ* phase formation during one-pot solid-state reactive sintering at 1450 °C to achieve a homogeneous distribution of both perovskite and fluorite phases. These phases self-organized into an optimal percolation structure that enhanced ion transport by establishing continuous conduction pathways for each phase. In contrast to mechanical mixing, the one-pot approach suppressed slow cation diffusion and prevented the formation of secondary phases. This method ensured excellent chemical compatibility and a stable microstructure, thereby facilitating densification. Relative densities exceeding 95% were attained, alongside tunable co-ionic conductivity. As a result, the BCZY-YDC-2-1 composite exhibited a total conductivity of 3.91 × 10^−3^ S cm^−1^ at 600 °C in wet air (Fig. 20e) and activation energies as low as 0.56–0.65 eV, attributed to optimized interfacial ion transport. The composite also demonstrated excellent long-term stability, with no new phases detected after 100 hours of conductivity testing and 150 hours under gradient thermal treatment (Fig. 20f). The anode-supported cell with a 13 µm thick BCZY-YDC-2-1 composite electrolyte delivered a PPD of 198 mW cm^−2^ and an OCV of 1.07 V at 600 °C under a dry H_2_/air gradient. In conclusion, self-assembly strategies represent a promising alternative to mechanical mixing for the preparation of electrolyte materials, although potential side reactions between phases still require careful avoidance.^268^

#### 3.7.3 Design and applications of anode materials

Self-assembly strategies enable the uniform integration and precise regulation of multiphase nanostructures at the atomic scale. Typically, researchers employ one-step synthesis to achieve molecular-level mixing of metal ions in sol–gel precursors. After heat treatment, composite oxides with well-defined heterointerfaces are formed. Under a reducing atmosphere, this structure can further undergo *in situ* reconstruction, precipitating highly dispersed and strongly anchored multicomponent alloy nanoparticles (NiCoFe, Ru, FeNiRu, CoFeNi, *etc.*), and forming metal/oxide multiphase heterostructures.^100,269–271^ The advantages of this structure lie in significantly increasing the electrochemically active surface area and enhancing electron and ion transport at interfaces; improving the nanoparticles' resistance to sintering and carbon deposition through strong metal–support interactions; and simultaneously regulating surface oxygen vacancy concentration and adsorption behaviours, thereby enhancing the anode's electrocatalytic activity and long-term operational stability for various fuels including hydrogen, methanol, methane, and even ammonia. Therefore, self-assembly methods provide an effective approach for constructing high-performance, durable fuel cell anodes with controllable structures and simplified processes.

**Fig. 21:**
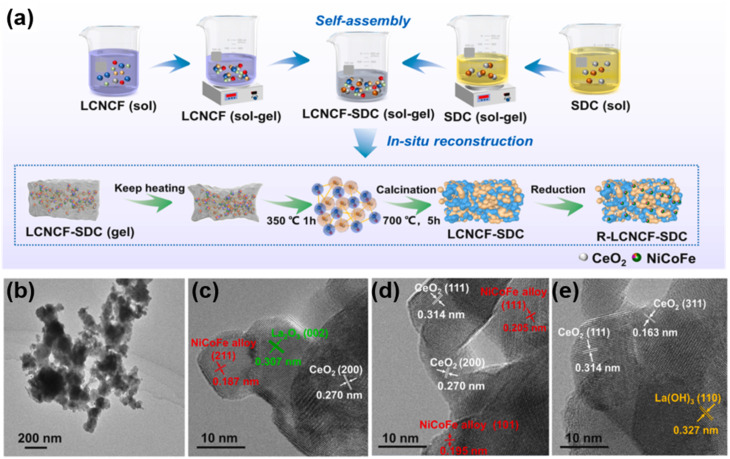
(a) Schematic diagram of the preparation process of R-LCNCF-SDC. (b) TEM micrographs of R-LCNCF-SDC powder. (c–e) HRTEM images of R-LCNCF-SDC powder after reduction. Reproduced with permission.^100^ Copyright 2024, Elsevier.

Gan *et al.* synthesized La_0.9_Ce_0.1_Ni_0.7_Co_0.15_Fe_0.15_O_3−*δ*_-Sm_0.2_Ce_0.8_O_2_ (LCNCF-SDC) composite anode materials using a self-assembly strategy.^100^ This approach enables the uniform distribution of multi-phase components at the nanoscale and constructs a NiCoFe/CeO_2_/La_2_O_3_ multi-phase heterostructure with rich heterointerfaces through *in situ* reduction, as shown in Fig. 21. The self-assembly process is achieved *via* a sol–gel method combined with one-step calcination, effectively enhancing the interaction between phases and improving the chemical and thermal compatibility of the material. This leads to the induction of high-density and highly dispersed active sites in a reducing atmosphere, promoting the formation of oxygen vacancies and the reconstruction of the electronic structure. The self-assembly strategy significantly enhances the electrochemical performance and structural stability of the anode. At 600 °C, when methanol is used as the fuel, the PPD of the single cell with the R-LCNCF-SDC anode reaches 0.94 W cm^−2^, demonstrating excellent low-temperature activity. EIS analysis indicates that the polarization resistance of this anode at 600 °C is significantly lower than that of the comparison samples, attributed to its enhanced hydrogen adsorption/desorption ability and oxygen ion transport kinetics. Further analysis of the DRT reveals that the resistance in both the low-frequency region (gas diffusion and surface processes) and the mid-frequency region (ion migration) is significantly reduced, indicating that the multi-phase heterostructure induced by self-assembly effectively promotes the synergistic optimization of reaction pathways.

Zhu *et al.* constructed a BaCo_0.43_Fe_0.45_Ni_0.17_O_3−*δ*_/BaCe_0.8_Y_0.2_O_3−*δ*_ (BMO7/BCY3) anode catalytic layer using a self-assembly strategy.^271^ The core advantage of this approach lies in the uniform self-assembly of two phases (cubic BMO and orthorhombic BCY) at the nanoscale in a single step, which in turn induces the *in situ* dissolution of CoFeNi ternary alloy nanoparticles in a reducing atmosphere. This strategy not only precisely constructs highly dispersed and small-sized (about 50 nm) alloy catalysts but also enhances the surface alkalinity through the precipitation of BaO, which, in synergy with the proton conduction ability of the BCY phase, promotes the adsorption–desorption kinetics of the ammonia decomposition reaction. Research shows that the formation of the CFN alloy significantly enhances ammonia adsorption capacity and reduces the energy barrier for nitrogen desorption, thereby achieving efficient ammonia decomposition at low temperatures, a synergistic effect that traditional single-metal catalysts lack. The cell with the BMO7/BCY3 catalytic layer achieved a PPD of 448 mW cm^−2^ when using ammonia as fuel at 550 °C, significantly higher than the 402 mW cm^−2^ of the cell without a catalytic layer. EIS revealed that the total polarization resistance of the cell in ammonia was significantly reduced, and the DRT analysis further confirmed that the low-frequency impedance increase was small, indicating that the catalytic layer effectively alleviated the gas diffusion limitation at the anode in an ammonia environment. In terms of stability, the cell without a catalytic layer failed after 43 h of operation at 550 °C and 200 mA cm^−2^, while the cell with the BMO7/BCY3 catalytic layer stably operated for over 60 h under the same conditions, with negligible voltage decay. SEM characterization indicated that the catalytic layer effectively inhibited the coarsening and agglomeration of Ni particles, maintaining the integrity of the anode microstructure. These results fully demonstrate that the self-assembled CFN/BCY composite catalytic layer has significant advantages in enhancing the low-temperature output performance, reducing polarization resistance, and improving long-term stability of direct ammonia proton ceramic fuel cells.

### 3.8 Surface reconstruction

The B-site cations of perovskites are usually transition metal elements, and they can precipitate from the lattice when the energy reaches the cation diffusion barrier. These nanoparticles serve as active sites for surface catalytic reactions and can improve the electrochemical performance of materials. Many strategies have been developed to induce cation segregation, such as defect induction and external atmosphere induction. Since the perovskite material used as an anode is in a reducing atmosphere, cations can precipitate from the main phase lattice to modify the surface reaction sites. For the cathode operating in an oxidizing atmosphere, the precipitation of B-site cations can also be promoted by constructing A-site defects, and the formed oxide nanoparticles have a positive effect on the ORR.

#### 3.8.1 Design and applications of cathode materials

In recent years, surface reconstruction has been proposed as an effective strategy for material modification, which significantly enhances the electrocatalytic performance and durability of electrodes by *in situ* formation of highly active nanostructures on their surfaces. Based on the typical perovskite material BCFZY, Chen *et al.* designed a novel composite material Ba(Co_0.4_Fe_0.4_Zr_0.1_Y_0.1_)_0.95_Ni_0.05_F_0.1_O_2.9−*δ*_ (N-BCFZYNF), by introducing Ni at the B-site and F at the oxygen site.^272^ During high-temperature treatment, this material undergoes surface reconstruction, leading to the *in situ* exsolution of NiO nanoparticles and the substitution of lattice oxygen by fluorine. This process significantly weakens the metal–oxygen bonds and enhances the lattice oxygen activity as well as proton migration capability. Single cells employing N-BCFZYNF as the cathode exhibited PPDs of 779, 545, and 374 mW cm^−2^ at operating temperatures of 600, 550, and 500 °C, respectively, which are markedly higher than those of the unmodified BCFZY electrode. To evaluate the operational stability of the N-BCFZYNF electrode, a durability test was conducted at 550 °C at a constant current density of 0.42 A cm^−2^ for 60 h. The cell voltage remained nearly stable, remaining approximately 0.79–0.75 V. Furthermore, EIS and DRT analysis revealed that surface reconstruction significantly improves the oxygen surface exchange rate and proton migration capability, maintaining stable electrocatalytic performance even in humidified air. Liang *et al.* engineered a precursor with a nominal composition of Ba_0.95_(Co_0.4_Fe_0.4_Zr_0.1_Y_0.1_)_0.95_Ni_0.05_O_3−*δ*_ (BCFZYN-095) *via* selective cation exsolution in an oxidizing atmosphere.^273^ After calcination, this material spontaneously formed a nanocomposite structure consisting of a major perovskite phase (m-BCFZYN-095) and secondary NiO surface nanoparticles. This unique structure, achieved through A-site deficiency-driven selective Ni leaching, was confirmed by XRD Rietveld refinement and HR-TEM/EDS elemental mapping. The exsolved NiO nanoparticles remained stable after prolonged annealing at 550 °C in air, demonstrating excellent structural and phase stability under oxidizing conditions. Electrochemical performance tests indicated that the BCFZYN-095 cathode exhibits outstanding ORR activity and triple conductivity. In symmetric cell tests, it achieved an ASR as low as 0.36 Ω cm^2^ at 550 °C. When assembled into single cells with a SDC electrolyte, a PPD of 1170 mW cm^−2^ was achieved at 550 °C; with a BZCYYb electrolyte, the PPD reached 540 mW cm^−2^ at the same temperature. The performance in both configurations surpassed that of the non-deficient BCFZYN cathode. Moreover, the single cell operated continuously for over 400 h at 550 °C without significant performance degradation, demonstrating exceptional operational stability. Using a perovskite material with the nominal composition Ba_0.8_Gd_0.8_Pr_0.4_Co_2_O_5+*δ*_ as an example, Zhu *et al.* systematically elucidated its spontaneous reconstruction at high temperature into a double perovskite Ba_0.8_Gd_0.8−*x*_Pr_0.4_Co_2−*y*_O_5+*δ*_ (BGPC) framework decorated with surface Gd_*x*_Co_*y*_O_3−*δ*_ (GCO) nanoparticles.^274^ This composite structure, with uniformly distributed GCO nanoparticles, formed naturally after calcination at 950 °C without requiring additional steps. The *in situ* surface reconstruction not only extended the TPBs but also enhanced the transport of protons and oxygen ions. In symmetric cell tests, the GCO-BGPC electrode exhibited an ASR of only 0.270 Ω cm^2^ at 600 °C in humidified air, indicating excellent ORR catalytic activity. When applied in an anode-supported single cell with a BZCYYb electrolyte, a PPD of 0.589 W cm^−2^ was achieved at 600 °C, and stable operation was maintained for 210 h at a constant current density of −0.5 A cm^−2^.

**Fig. 22:**
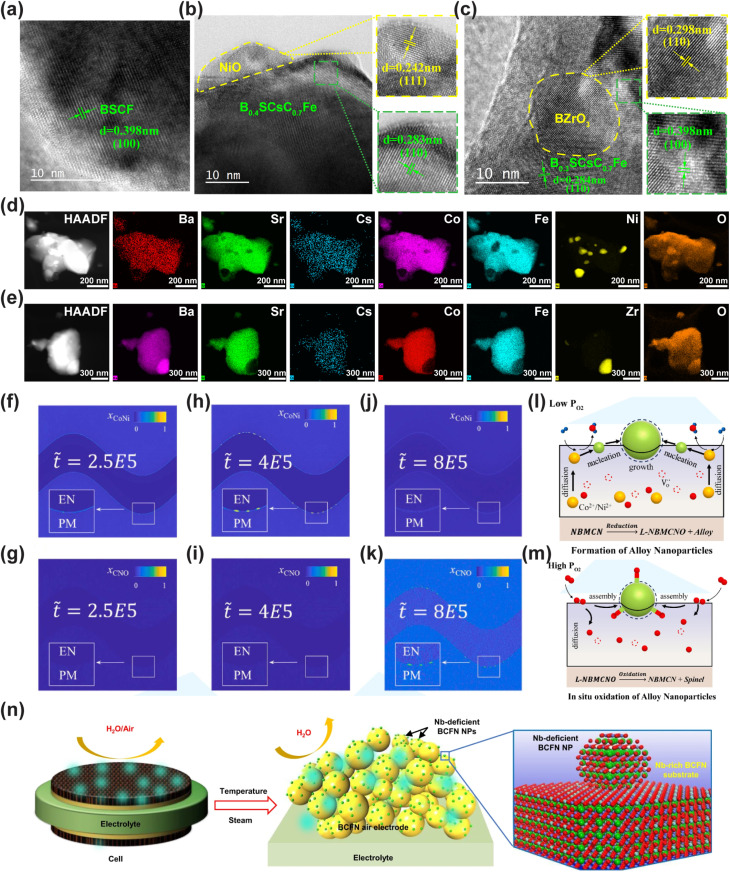
HR-TEM images of (a) BSCF, (b) BSCsCFNi, and (c) BSCsCFZr samples. HAADF-STEM images and corresponding EDS mapping of (d) BSCsCFNi and (e) BSCsCFZr samples. Reproduced with permission.^275^ Copyright 2025, Wiley-VCH GmbH. Contour plots of the molar fraction of CoNi (*x*_CoNi_) and CNO (*x*_CNO_) derived from phase field simulations under varying atmospheric conditions. (f and g) 2.5 × 10^5^th time step, (h and i) 4 × 10^5^th time step, and (j and k) 8 × 10^5^th time step. The CoNi alloy oxidized to form CNO oxide on the NBMCN perovskite surface is simulated by decreasing the *L* value from 4.5 to 2.5 at the 4 × 10^5^th time step (PM-perovskite matrix phase and EN-environmental atmosphere phase). (l) Illustration of CoNi alloy nanoparticles exsolved from NBMCN in a wet hydrogen atmosphere. (m) Illustration of *in situ* oxidation of CoNi alloy nanoparticles to CNO spinel in an air atmosphere. Reproduced with permission.^111^ Copyright 2024, Elsevier. (n) A schematic illustration of the formation of Nb-deficient BCFN NPs on the BCFN air electrode. Reproduced with permission.^276^ Copyright 2022, Springer Nature.

Our group developed two types of nanocomposites based on BSCF *via* an innovative surface reconstruction approach under an oxidizing atmosphere: Ba_0.4_Sr_0.5_Cs_0.1_Co_0.7_Fe_0.2_Ni_0.1_O_3−*δ*_ (BSCsCFNi) and Ba_0.4_Sr_0.5_Cs_0.1_Co_0.7_Fe_0.2_Zr_0.1_O_3−*δ*_ (BSCsCFZr).^275^ These materials were applied in O–CFCs and PCFCs, respectively, achieving synergistic optimization of bulk and surface properties. In BSCsCFNi, Ni exsolved and enriched on the surface as NiO nanoparticles, while in BSCsCFZr, Zr formed BaZrO_3_ (BZO) nanoparticles, as illustrated in Fig. 22a–e. This surface reconstruction enhanced the electrode's oxygen surface exchange capability, hydration ability, and bulk conduction of oxygen ions and protons. Furthermore, A-site doping with Cs^+^ promoted the formation of oxygen vacancies and reduced the energy barrier for proton migration, thereby optimizing the overall electrochemical performance. In terms of electrochemical performance, the CFC employing BSCsCFNi as the air electrode achieved PPDs of 1.047, 0.652, and 0.340 W cm^−2^ at 600, 550, and 500 °C, respectively. The PCFC using BSCsCFZr delivered PPDs of 0.936, 0.638, and 0.338 W cm^−2^ at the same temperatures. Moreover, both electrodes demonstrated excellent long-term durability. Symmetric cells fabricated with BSCsCFNi and BSCsCFZr electrodes showed only a slight increase in ASR after 1000 h of operation in air. Computational and experimental results indicated that Cs^+^ doping, along with surface reconstruction involving NiO and BZO nanoparticles, significantly improved ORR kinetics, extended the TPB, and provided new material design insights for highly efficient and stable operation of CFCs. In another study from our group, a reversible phase transformation-induced exsolution strategy was employed to uniformly reconstruct (Co,Ni)_3_O_4_ (CNO) nanoparticles on the surface of the perovskite oxide (Nd_0.5_Ba_0.5_)_0.95_Mn_0.7_Co_0.15_Ni_0.15_O_3−*δ*_ (NBMCN), forming a CNO@NBMCN heterostructured electrode.^111^ This surface reconstruction process not only enhanced the electrode's oxygen adsorption and dissociation capabilities but also significantly increased the oxygen vacancy concentration by inducing Mn–O octahedral distortion and weakening the covalency of metal–oxygen bonds. Phase-field simulations further revealed that under high-temperature oxidizing conditions, the CNO nanoparticles remained firmly anchored on the perovskite substrate, avoiding redissolution or coarsening and thereby ensuring structural stability during long-term operation (Fig. 22f–m). Electrochemically, the CNO@NBMCN electrode exhibited a very low ASR of 0.38 Ω cm^2^ at 600 °C, substantially superior to that of the unmodified NBMCN electrode (0.66 Ω cm^2^). Single cells based on this heterostructured electrode delivered PPDs of 0.73 and 0.46 W cm^−2^ at 600 and 550 °C, respectively. Additionally, a symmetric cell using this electrode showed a degradation rate of only 0.01% h^−1^ after 1000 h of operation in dry air at 600 °C, demonstrating outstanding durability.

A H_2_O-induced surface reconstruction strategy, which forms heterogeneous nanostructures under operating conditions, can also significantly enhance the catalytic activity of electrodes. Pei *et al.* designed an A-site deficient perovskite material, Ba_0.9_Co_0.7_Fe_0.2_Nb_0.1_O_3−*δ*_ (BCFN), and systematically elucidated its microscopic transformation under steam into an Nb-rich BCFN substrate covered with Nb-deficient BCFN nanoparticles.^276^ Under typical operating conditions, H_2_O-induced surface reconstruction led to the formation of a composite electrode with a heterogeneous structure (Fig. 22n). The *in situ* formed nanoparticles not only provided more active sites but also significantly enhanced proton and oxygen ion conduction. In symmetric cell tests, the BCFN electrode exhibited an ASR as low as 0.197 Ω cm^2^ at 600 °C in humidified air, indicating excellent ORR catalytic activity. When applied in an anode-supported single cell with a BZCYYb electrolyte, PPDs of 1.207, 0.82, and 0.55 W cm^−2^ were achieved at 600, 550, and 500 °C, respectively, demonstrating superior cell performance. The cell also operated stably for over 100 h at 600 °C and a current density of 0.5 A cm^−2^. Calculation results indicated that the segregation tendency of B-site cations in BCFN follows the order Co > Fe > Nb, meaning Co and Fe preferentially segregate to the surface, forming Nb-deficient nanoparticles, while the bulk becomes Nb-enriched. This results in the formation of Nb-deficient nanoparticles with enhanced surface activity. The *in situ* formed Nb-deficient nanoparticles not only improved electrocatalytic activity but also enhanced thermal and structural stability by reducing the risk of particle agglomeration. Chang *et al.* significantly improved the electrocatalytic performance and durability of the composite air electrode Sr_2_Fe_1.5_Mo_0.5_O_6−*δ*_-0.05SnO_2_ (SFMS50) by dynamically regulating its composition and structure.^277^ The composite electrode material is based on Sr_2_Fe_1.5_MoO_6−*δ*_ (SFM) perovskite, and SrMoO_4_ (SMO) and SrSnO_3_ (SSO) are introduced as the second phases. The core innovation lies in the introduction of SnO_2_ as a phase regulator, which reacts with Sr^2+^ dissolved from the SFM lattice under humid conditions, *in situ* forming SSO and SMO phases. This reconstructs the TPB and enhances the transport of protons, oxygen ions, and electrons. Structural characterization by XRD, TEM, and XPS confirmed that after treatment at 600 °C in a 30% H_2_O atmosphere, the contents of the SSO and SMO phases increased from 0.7 wt% and 2.0 wt% to 3.2 wt% and 5.8 wt%, respectively, while that of the main SFM phase decreased to approximately 91.2 wt%. This reconstruction effectively suppressed detrimental Sr segregation and increased oxygen vacancy concentration and surface hydration capability, as further supported by H_2_O-TPD and EPR results. Electrochemical performance tests demonstrated that the reconstructed SFMS50 electrode exhibits excellent activity. Single cells based on this electrode achieved PPDs of 0.71 and 0.38 W cm^−2^ at 600 and 550 °C, respectively, significantly outperforming the pristine SFM electrode. The electrode also showed remarkable durability, operating for 450 h at 600 °C under 400 mA cm^−2^ without significant degradation. Theoretical calculations further revealed that the SSO/SFM heterojunction reduces the energy barrier for the ORR and promotes proton transport through optimized hydration energy, thereby enhancing the overall reaction kinetics.

In summary, the surface reconstruction strategy, *via* the *in situ* formation of nanoparticle-decorated composite structures, significantly enhances the low-temperature electrochemical performance and durability of cathodes, providing a feasible materials design pathway for developing highly efficient and stable CFCs.

#### 3.8.2 Design and applications of anode materials

A direct ammonia proton ceramic fuel cell (DA-PCFC) is an environmentally friendly technology that efficiently converts ammonia chemical energy into electrical energy, but its development is limited by the insufficiency of catalytic activity and stability of ammonia electrodes.^278^

**Fig. 23:**
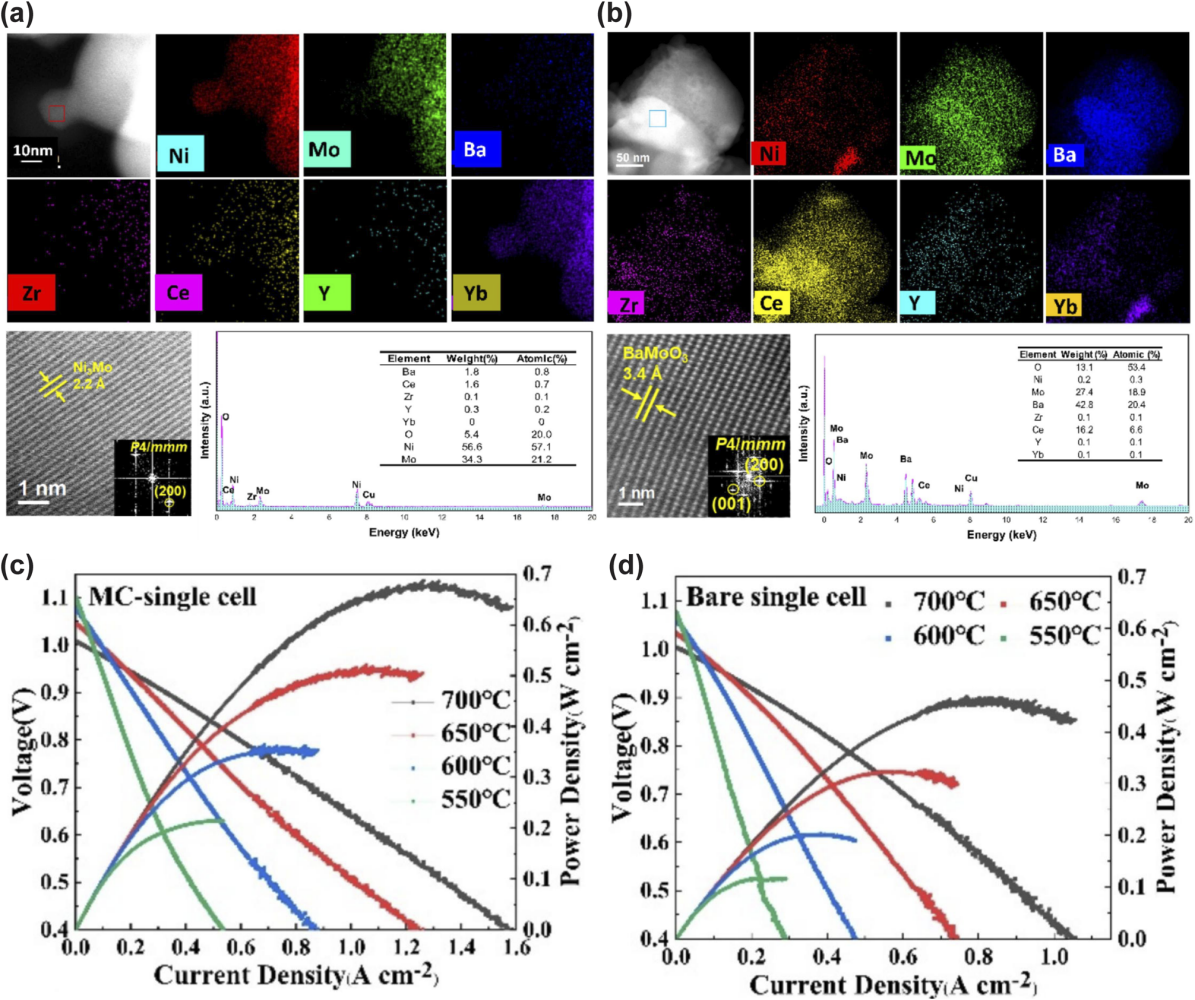
(a) HAADF-STEM image of Ni_3_Mo and the corresponding elemental mappings of Ni, Mo, Ba, Zr, Ce, Y, and Yb, and the zoomed-in HAADF-STEM image of the red-boxed area and representative EDS spectrum acquired from the region. (b) HAADF-STEM image of BaMoO_3−*δ*_ and the corresponding elemental mappings of Ni, Mo, Ba, Zr, Ce, Y, and Yb, and the zoomed-in HAADF-STEM image of the blue-boxed area and representative EDS spectrum acquired from the region. (c and d) *I*–*V*–*P* curves of a bare single cell and MC-single cell fed with NH_3_ in the anode and air in the cathode at 700–550 °C. Reproduced with permission.^279^ Copyright 2025, Elsevier.

Lan *et al.* developed an *in situ* generated heterogeneous catalyst to enhance the performance and durability of DA-PCFCs by optimizing the anode design.^279^ The heterogeneous catalyst is composed of Mo–Ni alloy nanoparticles (Ni_3_Mo) and the BaMoO_3−*δ*_ perovskite oxide phase. It is *in situ* generated through the interaction between the MoO_3−*δ*_ catalytic layer and the Ni-BZCYYb anode under operating conditions. XRD and HAADF-STEM analysis confirmed the formation of Ni_3_Mo and BaMoO_3−*δ*_. EDS quantitative analysis showed that the Ni : Mo atomic ratio was close to 3 : 1, and the Ba : Mo : O atomic ratio was 1 : 1 : 3 (Fig. 23a and b). The heterogeneous catalyst reduced the polarization resistance of the cell by 68% (from 13.98 to 4.44 Ω cm^2^ at 600 °C). The activation energy decreased from 1.351 eV to 0.848 eV, indicating an enhanced potential for low-temperature operation. After operating in an ammonia atmosphere for 1000 h at 650 °C, the degradation rate of the symmetrical cell was only 1.5 × 10^−4^ Ω cm^2^ h^−1^, which was two orders of magnitude lower than that of the unmodified cell (3.7 × 10^−2^ Ω cm^2^ h^−1^). The PPD values of a single cell reached 0.69, 0.52, 0.36, and 0.21 W cm^−2^ at 700, 650, 600, and 550 °C (Fig. 23c), respectively, an increase of 48.20%, 61.42%, 80.42%, and 82.07% compared to that of the unmodified cells (the lower the temperature, the more significant the increase) (Fig. 23d). At 650 °C, the degradation rate of the single cell was only 0.12% per 100 h after operation under a constant current of 0.32 A cm^−2^ for 478 h, far exceeding the stability record of DA-PCFC reported in the literature.^280^ A breakthrough in the activity and durability of DA-PCFCs was achieved through the *in situ* generation of Mo–Ni and BaMoO_3−*δ*_ heterogeneous catalysts. This work provides a new paradigm for enhancing the performance of fuel cells through innovative anode design and holds significant application prospects in the field of energy conversion technology.

To allow readers to directly compare the relative performance of different materials, we summarize the relevant data in Table 1, including the ASR of symmetric cells and the PPD of single cells for various materials covered in this review.

**Table 1:** ASR and PPD values of LT-CFCs at temperatures below 600 °C^a^

Design strategies	Cathode	Electrolyte	Anode		Temperature			Refs
					600 °C	550 °C	500 °C	
Machine learning	SCCN	SDC	Ni + SDC	ASRs (Ω cm^2^)	0.035	0.075	0.25	106
				PPDs (W cm^−2^)	1.52	1.19	∼0.70	
	SCTV	GDC	Ni + GDC	ASRs (Ω cm^2^)	0.019	0.033	0.066	113
				PPDs (W cm^−2^)	—	—	1.36	
	BLFC15	BZCYYb	Ni + BZCYYb	ASRs (Ω cm^2^)	—	—	∼1.25	116
				PPDs (W cm^−2^)	∼0.64	∼0.35	∼0.20	
	LBC	BZCYYb 4411	Ni + BZCYYb 4411	ASRs (Ω cm^2^)	0.03	∼0.07	∼0.21	118
				PPDs (W cm^−2^)	1.0	0.79	0.56	
	PLNSY	BZCYYb	Ni + BZCYYb	ASRs (Ω cm^2^)	∼0.10	0.26	∼0.85	119
				PPDs (W cm^−2^)	∼1.08	∼0.72	∼0.45	
DFT	D-BFZ	BZCYYb	Ni + BZCYYb	ASRs (Ω cm^2^)	∼0.20	∼0.4	∼1.10	135
				PPDs (W cm^−2^)	1.28	∼0.9	0.67	
	BSCCFN	SDC	Ni + SDC	ASRs (Ω cm^2^)	0.059	0.146	0.402	136
				PPDs (W cm^−2^)	1.12	0.83	0.48	
	BSCCFN	BZCYYb	Ni + BZCYYb	ASRs (Ω cm^2^)	0.22	0.495	1.021	
				PPDs (W cm^−2^)	0.84	0.57	0.29	
	BCFZYN	BZCYYb	Ni + BZCYYb	ASRs (Ω cm^2^)	∼0.15	∼0.30	∼0.70	137
				PPDs (W cm^−2^)	0.936	0.663	0.427	
	(BSCFW)@PBSCF	BZCYYb	Ni + BZCYYb	ASRs (Ω cm^2^)	0.36	0.80	1.39	139
				PPDs (W cm^−2^)	0.90	0.57		
	BCFN	BHCYb172	Ni + BHCYb172	ASRs (Ω cm^2^)				148
				PPDs (W cm^−2^)	1.74	1.33		
	PBCC–BNCYb (7 : 3)	BNCYb	Ni + BNCYb	ASRs (Ω cm^2^)				149
				PPDs (W cm^−2^)	1.12	0.73	0.42	
	PBSCF	BHCYYb-3511	Ni + BHCYYb-3511	ASRs (Ω cm^2^)				151
				PPDs (W cm^−2^)	1.1			
	BPHYC	BSCYb172	Ni + BSCYb172	ASRs (Ω cm^2^)				152
				PPDs (W cm^−2^)	1.57	1.21	0.82	
High-entropy strategy	PNMCFC-PBC	BZCYYb	Ni + BZCYYb	ASRs (Ω cm^2^)	∼0.30	0.72	∼2.10	160
				PPDs (W cm^−2^)	1.72	1.30	0.94	
	BCFZSP	BZCYYb	Ni + BZCYYb	ASRs (Ω cm^2^)	∼0.18	0.448	∼0.60	161
				PPDs (W cm^−2^)	0.67	0.48	0.33	
	LPNSBSCF	BZCYYb	Ni + BZCYYb	ASRs (Ω cm^2^)				162
				PPDs (W cm^−2^)	0.81	0.54	0.33	
	HE-PBSLCC	BZCYYb	Ni + BZCYYb	ASRs (Ω cm^2^)	0.26	0.75	2.13	165
				PPDs (W cm^−2^)	1.16	0.72	0.40	
	LPNBSN	BCZY	Ni + BCZY	ASRs (Ω cm^2^)	∼0.08			108
				PPDs (W cm^−2^)	1.87			
	N-XFN	BZCYYb	Ni + BZCYYb	ASRs (Ω cm^2^)	0.85	1.96		167
				PPDs (W cm^−2^)	0.79	0.43		
	BCFZY	BSZCYYbD	Ni + BZCYYb	ASRs (Ω cm^2^)				85
				PPDs (W cm^−2^)	0.318	0.246	0.166	
	BCFZY	BHSZCYYb	Ni + BHSZCYYb	ASRs (Ω cm^2^)				181
				PPDs (W cm^−2^)	0.720	0.43	0.25	
	BCFZY	Ba_1.05_Ce_0.45_ZYYbPr_0.10_Gd_0.15_	Ni + BZCYYb	ASRs (Ω cm^2^)				183
				PPDs (W cm^−2^)	0.397	0.284		
Defect engineering	bs-PBSCF	BZCYYb	Ni + BZCYYb	ASRs (Ω cm^2^)	0.27	∼0.50	1.45	198
				PPDs (W cm^−2^)	1.075	0.798	0.583	
	B_0.9_CFZ	BZCYYb	Ni + BZCYYb	ASRs (Ω cm^2^)	0.55	1.13		199
				PPDs (W cm^−2^)	0.39		0.19	
	B_0.9_CFZY	BZCY	Ni + BZCY	ASRs (Ω cm^2^)	∼0.14	∼0.25	0.52	200
				PPDs (W cm^−2^)	0.669	0.548	0.376	
	BCFZYN-095-01	BZCYYb	Ni + BZCYYb	ASRs (Ω cm^2^)	∼0.14	0.25	∼0.51	201
				PPDs (W cm^−2^)	1.1	∼0.90	∼0.65	
	D-SFN	BZCYYb	Ni + BZCYYb	ASRs (Ω cm^2^)	0.404	1.209	4.81	40
				PPDs (W cm^−2^)	0.48	0.36	0.24	
	BCFZY	BCSDCu	Ni + BCSDCu	ASRs (Ω cm^2^)				206
				PPDs (W cm^−2^)	0.39	0.33		
	BCFZY	BZCYYb-0.95	Ni + BZCYYb-0.95	ASRs (Ω cm^2^)				207
				PPDs (W cm^−2^)	0.56	0.38		
	SFNM-GDC	GDC	SFNM + GDC	ASRs (Ω cm^2^)	7.52 (H_2_)			214
					9.37 (NH_3_)			
				PPDs (W cm^−2^)				
Mechanical mixing	SFM0.07+GDC	YSZ	Ni + YSZ	ASRs (Ω cm^2^)	1.647			215
				PPDs (W cm^−2^)	0.35			
	SMO-GDC	YSZ	Ni + YSZ	ASRs (Ω cm^2^)	2.38			216
				PPDs (W cm^−2^)	0.107			
	PBSCF-20SZM	YSZ/GDC	Ni + YSZ	ASRs (Ω cm^2^)	0.15	0.40	1.5	217
				PPDs (W cm^−2^)	0.534	0.291	0.155	
	c-SYNC	YSZ/SDC	Ni + YSZ	ASRs (Ω cm^2^)	0.063	0.175		218
				PPDs (W cm^−2^)	∼0.53			
	NTE-BSCF	YSZ/GDC	Ni + YSZ	ASRs (Ω cm^2^)	0.028	0.065		219
				PPDs (W cm^−2^)	∼0.60	∼0.30		
	Sr_vac_/LSCF095	YSZ/GDC	Ni + YSZ	ASRs (Ω cm^2^)	∼1.3			221
				PPDs (W cm^−2^)	0.35			
	V-PSC/2W	GDC	Ni + GDC	ASRs (Ω cm^2^)	∼0.5	∼0.95	∼2.25	222
				PPDs (W cm^−2^)	0.958	0.749	0.536	
	2Ru-BCF	BZCYYb	Ni + BZCYYb	ASRs (Ω cm^2^)	0.48	∼1.1	∼3.0	223
				PPDs (W cm^−2^)	0.97	0.58		
	BCFZY	BL91	Ni + BZCY	ASRs (Ω cm^2^)				234
				PPDs (W cm^−2^)	0.22			
	BCZY63+BCFZY	BZCYYb + 1 wt% NiO	Ni + BZCYYb	ASRs (Ω cm^2^)				26
				PPDs (W cm^−2^)			0.455	
	BCFZY	BZY20 + 1.4 wt% CuO	Ni + BZY20	ASRs (Ω cm^2^)				
				PPDs (W cm^−2^)	0.29	0.215	0.142	
Impregnation strategy	BCO-LSCF	BZCYYb	Ni + BZCYYb	ASRs (Ω cm^2^)	0.17		0.84	245
				PPDs (W cm^−2^)	1.16	0.78	0.41	
	PFC-PBSCF	BZCYYb	Ni + BZCYYb	ASRs (Ω cm^2^)	0.81	1.88		247
				PPDs (W cm^−2^)	∼0.85			
	PNC–PrO_*x*_	BZCYYb	Ni + BZCYYb	ASRs (Ω cm^2^)	0.08			248
				PPDs (W cm^−2^)	1.56	1.31	1.04	
	PBNO-CCS	BZCYYb	Ni + BZCYYb	ASRs (Ω cm^2^)				249
				PPDs (W cm^−2^)	1.16	0.63	0.36	
	PBSCF	BZCYYb	RCN-Ni + BZCYYb	ASRs (Ω cm^2^)	3.08	8.97		258
				PPDs (W cm^−2^)	0.942	0.32	0.41	
Self-assembly	BFCS	BZCYYb	Ni + BZCYYb	ASRs (Ω cm^2^)	0.17			260
				PPDs (W cm^−2^)	0.61	0.33		
	SCFN2	SDC	Ni + SDC	ASRs (Ω cm^2^)	0.072	0.29	1.28	261
				PPDs (W cm^−2^)	0.977	0.702	0.451	
	LSCFC-PI	YSZ/GDC	Ni + YSZ	ASRs (Ω cm^2^)	0.124			262
				PPDs (W cm^−2^)	∼0.85	∼0.55		
	PBCNY	BZCYYb	Ni + BZCYYb	ASRs (Ω cm^2^)	0.24	0.547	1.585	263
				PPDs (W cm^−2^)	0.97			
	C-PBCF	BZCYYb	Ni + BZCYYb	ASRs (Ω cm^2^)	∼0.075			264
				PPDs (W cm^−2^)	0.88			
	C/H-BSCF	BZCYYb	Ni + BZCYYb	ASRs (Ω cm^2^)	0.26	0.68	2.13	265
				PPDs (W cm^−2^)	1.67	1.37	1.00	
	LSCF-BZCY	BZY-LCO	Ni-3BZY-7LCO	ASRs (Ω cm^2^)				266
				PPDs (W cm^−2^)	0.135			
	Ba–Ce–Fe–Co–O	BCZY-YDC-2-1	Ni + BCZY-YDC-2-1	ASRs (Ω cm^2^)				267
				PPDs (W cm^−2^)	0.198	0.146		
	70 wt% SDC-(Li_0.67_Na_0.33_)_2_CO_3_ + 30 wt% lithiated NiO	SDC-(Li_0.67_Na_0.33_)_2_CO_3_	LCNCF-SDC	ASRs (Ω cm^2^)				100
				PPDs (W cm^−2^)	0.94	0.58		
	BCFZY	BZCYYb	BMO7/BCY3	PPDs (W cm^−2^)	0.716 (H_2_)	0.514 (H_2_)	0.335 (H_2_)	271
				PPDs (W cm^−2^)	0.68 (NH_3_)	0.448 (NH_3_)	0.273 (NH_3_)	
Surface reconstruction	N-BCFZYNF	BZCYYb	Ni + BZCYYb	ASRs (Ω cm^2^)	0.165	0.407	1.338	272
				PPDs (W cm^−2^)	0.779	0.545	0.374	
	BCFZYN-095	SDC	Ni + SDC	ASRs (Ω cm^2^)		0.36		273
				PPDs (W cm^−2^)	∼1.58	1.17	∼0.70	
	GCO-BGPC	BZCYYb	Ni + BZCYYb	ASRs (Ω cm^2^)	0.270	0.667	1.881	274
				PPDs (W cm^−2^)	0.589			
	BSCsCFNi	SDC	Ni + SDC	ASRs (Ω cm^2^)	0.061	0.155	0.502	275
				PPDs (W cm^−2^)	1.047	0.652	0.340	
	BSCsCFZr	BZCYYb	Ni + BZCYYb	ASRs (Ω cm^2^)	0.279	0.588	1.285	
				PPDs (W cm^−2^)	0.936	0.638	0.338	
	CNO@NBMCN	BZCYYb	Ni + BZCYYb	ASRs (Ω cm^2^)	0.38	0.95	1.90	111
				PPDs (W cm^−2^)	0.73	0.46		
	BCFN	BZCYYb	Ni + BZCYYb	ASRs (Ω cm^2^)	0.197	0.471	1.197	276
				PPDs (W cm^−2^)	1.207	0.82	0.55	
	SFMS50	BZCYYb	Ni + BZCYYb	ASRs (Ω cm^2^)	0.205	0.404		277
				PPDs (W cm^−2^)	0.71	0.38		
	LSCF	BZCYYb	Ni_3_Mo + BaMoO_3−*δ*_+ Ni + BZCYYb	ASRs (Ω cm^2^)	4.44 (NH_3_)	10.33 (NH_3_)		279
				PPDs (W cm^−2^)	0.36 (NH_3_)	0.21 (NH_3_)		

a LSCF: La_0.6_Sr_0.4_Co_0.2_Fe_0.8_O_3−*δ*_, SCCN: Sr_0.9_Cs_0.1_Co_0.9_Nb_0.1_O_3_, SCTV: SrCo_0.8_Ta_0.15_V_0.05_O_3−*δ*_, BLFC15: Ba_0.95_La_0.05_Fe_0.85_Co_0.15_O_3−*δ*_, LBC: La_0.8_Ba_0.2_CoO_3_, PLNSY: (Pr_0.05_La_0.4_Nd_0.2_Sm_0.1_Y_0.25_)BaCo_2_O_5+*δ*_, D-BFZ: Ba_0.875_Fe_0.875_Zr_0.125_O_3−*δ*_, BSCCFN: Ba_0.4_Sr_0.5_Cs_0.1_Co_0.7_Fe_0.2_Nb_0.1_O_3−*δ*_, BCFZYN: Ba_0.95_(Co_0.4_Fe_0.4_Zr_0.1_Y_0.1_)_0.95_Ni_0.05_O_3−*δ*_, BSCFW@PBSCF: (Ba/Sr)(Co/Fe/W)O_3−*δ*_@PrBa_0.5_Sr_0.5_Co_1.5_Fe_0.5_O_5+*δ*_, BCFN: Ba_0.9_Co_0.7_Fe_0.2_Nb_0.1_O_3−*δ*_, BHCYb172: BaHf_0.1_Ce_0.7_Yb_0.2_O_3−*δ*_, PBCC: PrBa_0.8_Ca_0.2_Co_2_O_5+*δ*_, BNCYb: BaNb_0.05_Ce_0.7_Yb_0.25_O_3−*δ*_, BHCYYb-3511: BaHf_0.3_Ce_0.5_Y_0.1_Yb_0.1_O_3−*δ*_, BPHYC: Ba_0.9_Pr_0.1_Hf_0.1_Y_0.1_Co_0.8_O_3−*δ*_, BSCYb172: BaSn_0.1_Ce_0.7_Yb_0.2_O_3−*δ*_, PNMCFC: PrNi_0.2_Mn_0.2_Co_0.2_Fe_0.2_Cu_0.2_O_3−*δ*_, BCFZSP: BaCo_0.2_Fe_0.2_Zr_0.2_Sn_0.2_Pr_0.2_O_3−*δ*_, LPNSBSCF: (La_0.25_Pr_0.25_Nd_0.25_Sm_0.25_)Ba_0.5_Sr_0.5_Co_1.5_Fe_0.5_O_5+*δ*_, HE-PBSLCC: Pr_0.2_Ba_0.2_Sr_0.2_La_0.2_Ca_0.2_CoO_3−*δ*_, LPNBSN: La_0.4_Pr_0.4_Nd_0.4_Ba_0.4_Sr_0.4_NiO_4+*x*_, N-XFN: *x*NiO-Pr_0.2_La_0.2_Ba_0.2_Sr_0.2_Ca_0.2_Fe_0.8_Ni_0.2−*x*_O_3−*δ*_, BCFZY: BaCo_0.4_Fe_0.4_Zr_0.1_Y_0.1_O_3−*δ*_, BSZCYYbD: BaSn_0.16_Zr_0.24_Ce_0.35_Y_0.1_Yb_0.1_Dy_0.05_O_3−*δ*_, BHSZCYYb: BaHf_1/6_Sn_1/6_Zr_1/6_Ce_1/6_Y_1/6_Yb_1/6_O_3−*δ*_, B_0.9_CFZ: Ba_0.9_Co_0.7_Fe_0.2_Zr_0.1_O_3−*δ*_, B_0.9_CFZY: Ba_0.9_Co_0.4_Fe_0.4_Zr_0.1_Y_0.1_O_3−*δ*_, BCFZYN-095-01: Ba_0.95_(Co_0.4_Fe_0.4_Zr_0.1_Y_0.1_)_0.9_Ni_0.1_O_3−*δ*_, D-SFN: Sr_2.8_Fe_1.8_Nb_0.2_O_7−*δ*_, BCSDCu: BaCe_0.7_Sn_0.1_Dy_0.15_Cu_0.05_O_3−*δ*_, BZCYYb-0.95: Ba(Zr_0.1_Ce_0.7_Y_0.1_Yb_0.1_)_0.95_O_3−*δ*_, SFNM-GDC: Sr_1.9_Fe_1.4_Ni_0.1_Mo_0.5_O_6_-Gd_0.2_Ce_0.8_O_2_, SFM0.07: SrFe_0.93_Mo_0.07_O_3−*δ*_, SMO: SmMn_2_O_5_, SZM: Sm_0.85_Zn_0.15_MnO_3_, SYNC: Sr_*x*_(Y_*y*_(Nb_0.1_Co_0.9_)_1 *y*_)O_3−*δ*_, NTE-BSCF: HfW_2_O_8_+ Ba_0.5_Sr_0.5_Co_0.8_Fe_0.2_O_3−*δ*_, LSCF095: (La_0.6_Sr_0.4_)_0.95_Co_0.2_Fe_0.8_O_3−*δ*_, PSC: Pr_0.4_Sr_0.6_CoO_3−*δ*_, BCF: BaCe_0.125_Fe_0.875_O_3−*δ*_, BL91: 90 BaZr_0.1_Ce_0.7_Y_0.2_O_3−*δ*_-10 La_0.9_Sr_0.1_Ga_0.8_Mg_0.2_O_3−*δ*_, BCZY63: BaCe_0.6_Zr_0.3_Y_0.1_O_3−*δ*_, PFC: Pr_0.9_Fe_0.7_Co_0.3_O_3_, PNC: PrNi_0.7_Co_0.3_O_3−*δ*_, PBNO: Pr_1.8_Ba_0.2_NiO_4.1_, RCN: Ru–Cu–Ni, BFCS: BaFe_0.6_Ce_0.2_Sc_0.2_O_3−*δ*_, SCFN2: Sr_0.9_Ce_0.1_Fe_0.8_Ni_0.2_O_3−*δ*_, LSCFC-PI: La_0.6_Sr_0.4_Co_*x*_Fe_*y*_O_3−*δ*_/PrCoO_3_/Co_3_O_4_/PrO_2_, PBCNY: PrBaCo_1.8_Nb_0.1_Y_0.1_O_5+*δ*_, C-PBCF: cubic-BSCF, H-BSCF: Ba_4_Sr_4_(Co_0.8_Fe_0.2_)_4_O_16−*δ*_, BZY-LCO: BaZr_0.8_Y_0.2_O_3−*δ*_-La_2_Ce_2_O_7_, BCZY-YDC-2-1: BaCe_0.5_Zr_0.4_Y_0.1_O_3−*δ*_-Ce_0.5_Y_0.5_O_2−*δ*_, LCNCF: La_0.9_Ce_0.1_Ni_0.7_Co_0.15_Fe_0.15_O_3−*δ*_, BMO7/BCY3: BaCo_0.43_Fe_0.45_Ni_0.17_O_3−*δ*_/BaCe_0.8_Y_0.2_O_3−*δ*_, N-BCFZYNF: Ba(Co_0.4_Fe_0.4_Zr_0.1_Y_0.1_)_0.95_Ni_0.05_F_0.1_O_2.9−*δ*_, BCFZYN-095: Ba_0.95_(Co_0.4_Fe_0.4_Zr_0.1_Y_0.1_)_0.95_Ni_0.05_O_3−*δ*_, GCO: Gd_*x*_Co_*y*_O_3−*δ*_, BGPC: Ba_0.8_Gd_0.8−*x*_Pr_0.4_Co_2−*y*_O_5+*δ*_, BSCsCFNi: Ba_0.4_Sr_0.5_Cs_0.1_Co_0.7_Fe_0.2_Ni_0.1_O_3−*δ*_, BSCsCFZr: Ba_0.4_Sr_0.5_Cs_0.1_Co_0.7_Fe_0.2_Zr_0.1_O_3−*δ*_, CNO@NBMCN: (Co,Ni)_3_O_4_@(Nd_0.5_Ba_0.5_)_0.95_Mn_0.7_Co_0.15_Ni_0.15_O_3−*δ*_, BCFN: Ba_0.9_Co_0.7_Fe_0.2_Nb_0.1_O_3−*δ*_, and SFMS50: Sr_2_Fe_1.5_Mo_0.5_O_6−*δ*_-0.05SnO_2_.

## 4. Challenges and prospects

This review describes the progress on studying key materials of LT-CFCs in the past decade based on new types of material designs and end-use applications, and an outlook on the future development of these materials was also provided. Great progress has been made in the design and application of new LT-CFC materials by adopting new methods and strategies. LT-CFC technology is gradually moving towards application through material innovation and structural design. However, there are still many challenges in terms of materials before commercialization. We look forward to more methods and strategies being applied to the design of new materials to promote their application and development.

### 4.1 Electrolyte materials

It remains a challenging task to continuously design and develop new electrolyte materials with high ionic conductivity at low temperatures in order to reduce ohmic loss and improve electrochemical performance at low temperatures. The combination and optimization of existing electrolyte systems are still an alternative approach to improve the ionic conductivity at low temperatures and stability. The problem of thermal stress caused by the mismatch of thermal expansion coefficients between the electrolyte and electrode should be addressed, either by regulating the elements in the cell components or by using composite materials or their combination, in order to enhance the long-term stability of the LT-CFCs.

### 4.2 Electrode materials

Low ionic conductivity, insufficient reaction kinetics and poor stability remain common problems faced by electrode materials for LT-CFCs, and these issues need to be continuously addressed in the future. From this perspective, developing low-temperature CFCs with symmetrical electrodes is also an important research topic, in order to reduce the manufacturing cost of CFCs and simplify the preparation process. In addition, anode materials need to enhance the adaptability to hydrocarbon fuels, and solve issues such as carbon deposition and sulfur poisoning problems, and improve the thermal shock resistance. In direct ammonia ceramic fuel cells (DA-CFCs), the ammonia electrode (anode) not only needs to have high ionic and electronic conductivity, but also good catalytic activity for ammonia decomposition. However, ammonia can cause nitriding on most anode materials. This effect directly reduces the electrochemical performance of anode materials, leading to a decline in ammonia decomposition catalytic activity and deterioration in stability, which seriously restricts the development of AD-CFCs. Therefore, solving the problems of slow kinetics of the ammonia electrode oxidation reaction, easy poisoning and poor stability are the key issues faced in the development of AD-CFCs. In reversible CFCs, the problem of the performance attenuation of electrode materials in the oxidation–reduction cycles needs to be solved.

ML methods have demonstrated obvious advantages and potential capabilities in the design and screening of CFC materials. Many examples of combining ML with the experimental results have verified the effectiveness and feasibility of ML methods. However, we should also note that although these designed materials (such as cathodes) exhibit good electrochemical performance; their TECs are generally high. Therefore, there is still a lack of sufficient evidence for the long-term stability of CFC materials. The corresponding solution is to take the TEC into account when designing and screening CFC materials through ML techniques. In addition, although the ML techniques offer a solution for predicting the TEC of oxides, breaking through the computational limitations of traditional physics-based simulations and balancing accuracy and temperature coverage, the model performance is highly dependent on the quantity and diversity of training data and is unable to handle phase transitions and dynamic structural changes. Therefore, the descriptor system needs to be further expanded to break through the existing framework.

High-entropy oxide materials, with their unique structure and properties, have demonstrated significant potential in the electrolytes and electrodes (cathodes/anodes) of medium-to-low-temperature CFCs. However, they still face key challenges such as the optimization of ionic conductivity and the unclear correlation between the composition, structure, and performance. The composition space of CFC materials is vast, and it is difficult to screen efficient materials by traditional methods. Data-driven precise material design and automated synthesis that combines high-throughput computing with ML is a desirable approach. It can optimize element combinations, accelerate CFC material screening, and enhance the performance of electrolyte and electrode materials. Deepen mechanism research: utilizing *in situ* characterization techniques (such as *in situ* XRD and Raman spectroscopy) to reveal the structural evolution and performance degradation mechanisms of high-entropy oxide materials under CFC operating conditions, analyse the correlation system of composition-lattice distortion-ion transport/catalytic activity, and clarify the influence of defect chemistry on performance. For key materials (electrolyte and electrode) in CFCs, the challenges are not limited to the development of new compositions, but also include the understanding of new electrochemical and transport mechanisms. At present, the understanding of the electrochemical and transport mechanisms of the newly emerged high-entropy CFC materials is still insufficient: the specific action path of the multi-element “cocktail effect” and the quantitative relationship between configurational entropy and the above-mentioned performances/mechanisms remain unclear. Therefore, elucidating the precise tuning mechanism remains a persistent challenge.

Exsolution of nanoparticles is a promising technique for the design of nanostructured catalysts in energy conversion and storage devices, which plays an important role in improving electrochemical performance of electrodes. The nanoparticle-exsolution numbers are strongly dependent on the changing atmospheres. Taking the cathode as an example, the nanoparticle exsolution from the matrix is often obtained in a reducing atmosphere or under low oxygen partial pressure conditions. However, when the atmosphere is shifted into an oxidizing environment, the exsolved nanoparticles may convert back into the matrix due to the potential difference, thus resulting in a decrease in the exsolved nanoparticle numbers with operating times. In addition, the growth of exsolved nanoparticles over time is also a problem that cannot be ignored. Another issue that cannot be ignored also exists in the exsolution of multiple components in designed electrodes after treating and the decomposition of the original parent components. When cathode materials with exsolved nanoparticles are prepared in an oxidizing atmosphere, long-term operation of the cell can also lead to changes in the morphology of the nanoparticles. As a consequence, the stability of nanoparticle electrodes at high operating temperatures is a severe challenge. Therefore, in-depth study and experimental validation on the electrocatalytic performance and stability of electrodes are needed, in order to gain insight into the real impact of the growth kinetics of nanoparticles on catalytic performance and stability, although the literature has shown that CFCs with exsolved nanoparticle electrodes have good catalytic activity and stability within a limited time.

The characterization methods of materials play a crucial role in better understanding the mechanisms of material improvement and degradation (including electrode reaction mechanisms), providing new and in-depth insights for enhancing material performance. The electrochemical performance of CFCs is closely related to the transport and reaction of electrons and ions at the bulk phase and interface. Due to the fact that CFCs operate in a complex environment and the understanding of the dynamic structural evolution of materials is limited, it is difficult to identify the root cause of the decline in cell performance. *Ex situ* characterization techniques cannot provide real-time and accurate information. In process analysis and mechanism study, it is necessary to combine a variety of *in situ* characterization techniques and novel testing methods to analyze the evolution of structural compositions, bonding information, microscopic morphology, chemical properties, and physical properties of materials at the atomic scale during the reaction process of CFCs, to achieve the purpose of effectively analyzing the reaction mechanism of CFCs. Therefore, the application of *in situ* characterization techniques, such as *in situ* Fourier transform infrared spectroscopy, Raman spectroscopy, and electrochemical impedance spectroscopy, will provide new insights and support for the improvement of material properties and effective evaluation and analysis/understanding of reaction mechanisms and performance variation.

The cell components need to operate in different environments, so the stability of the cell components directly affects the reliability and lifespan of the CFCs. Currently, the structural (chemical) stability tests of electrode materials are all conducted before CFC testing, while the stability tests of materials after CFC testing are relatively rare (uncommon). After long-term operation of the CFCs, the composition of materials will change due to diffusion and reactions at the interfaces between two components or being exposed to the working atmosphere. This will lead to the performance degradation of the catalyst and electrolyte and a decrease in its long-term stability. This is not visible in the material performance characterization before CFC testing. To provide a comprehensive understanding of material failure and long-term stability, it is necessary to conduct post-death characterization of the tested CFCs. For instance, by observing the XRD, Raman spectra, SEM (EDS) surfaces and cross-sections before and after CFC testing, the changes in materials can be clearly compared, thereby providing targeted judgments. Therefore, the stability of the electrode material after CFC testing may be an important criterion for evaluating the stability of the electrode materials and understanding catalyst failure.

In CFCs, each cell component (electrolyte and electrode) plays multiple roles and must satisfy certain requirements, including some physical and chemical properties and functions in oxidizing and/or reducing atmospheres. The existing design methods for cell components target relatively single goals and it is difficult for them to meet the requirements of cell materials in different aspects, namely cell materials developed only through a single method still cannot fundamentally meet their multiple functional requirements. Therefore, it is necessary to research and design methods that can adapt to different targets in order to enhance their pertinence and adaptability. The multi-strategy combination methodology is expected to comprehensively enhance the performance of materials and overcome the limitations and one-sidedness of the traditional single method. The high-entropy design strategy based on multi-element synergy, the entropy stability effect and lattice and electronic structure regulation is expected to systematically solve the core contradiction of traditional electrode materials that “activity, stability and compatibility cannot be achieved simultaneously”. Combining machine learning to assist in optimizing the complex components of high-entropy oxides can accelerate the process of screening and optimizing components. This not only enables the design of the composition and proportion of high-entropy electrode materials based on specific performance, but also allows for the regulation of the performance of high-entropy electrode materials through multi-objective optimization, providing guidance for the rational design of high-performance electrode materials.

PCFCs, in addition to having the advantages of oxygen-ion conducting CFCs (O–SOFCs), have lower temperature dependence, longer service life and greater potential for commercial applications. With the continuous increase in research achievements and work accumulation related to PCFCs, the performance of PCFCs is constantly improving, which is conducive to its future commercial development. Therefore, conducting timely application research on PCFC stacks can be regarded as a research or effort direction for the future.

Ammonia, as a promising carbon-free fuel carrier, has the advantages of convenient storage and transportation, high energy density, and environmental friendliness. At temperatures above 600 °C, ammonia can be rapidly decomposed into nitrogen and hydrogen, and thus is suitable for DA-CFCs without external reforming. The coupled application of DA-CFCs and renewable energy will become a new growth point. The number of integrated wind-solar-ammonia projects will continue to increase, achieving sustainable energy supply and comprehensive utilization. Especially in the fields of energy storage and grid peak shaving, its application has also begun to enter a substantive promotion stage, and its application scenarios will continue to expand. Therefore, promoting the research on the commercial application of DA-CFCs has become an urgent task in the near future.

## Author contributions

Ying Zhang: visualization, investigation & writing – original draft & editing. Rui Guo: visualization, investigation & writing – original draft. Yu Shen: visualization, investigation & writing – original draft. Tianmin He: conceptualization, supervision, writing – original draft, writing – review & editing.

## Conflicts of interest

The authors declare no conflicts of interest.

## Data Availability

No primary research results, software or code have been included and no new data were generated or analysed as part of this review.
